# A Mixture of Endocrine Disrupting Chemicals Associated with Lower Birth Weight in Children Induces Adipogenesis and DNA Methylation Changes in Human Mesenchymal Stem Cells

**DOI:** 10.3390/ijms23042320

**Published:** 2022-02-19

**Authors:** Polina Lizunkova, Elin Engdahl, Gábor Borbély, Chris Gennings, Christian Lindh, Carl-Gustaf Bornehag, Joëlle Rüegg

**Affiliations:** 1 Department of Organismal Biology, Uppsala University, 75236 Uppsala, Sweden; polina.lizunkova@ebc.uu.se (P.L.); elin.engdahl@ebc.uu.se (E.E.); 2 The Swedish Toxicology Sciences Research Center (Swetox), 15257 Södertälje, Sweden; gab.borbely@gmail.com; 3 Department of Environmental Medicine and Public Health, Icahn School of Medicine at Mount Sinai, New York, NY 10029, USA; chris.gennings@mssm.edu (C.G.); carl-gustaf.bornehag@kau.se (C.-G.B.); 4 Occupational and Environmental Medicine, Lund University, 22363 Lund, Sweden; christian.lindh@med.lu.se; 5 Department of Health Sciences, Karlstad University, 65188 Karlstad, Sweden

**Keywords:** Endocrine Disrupting Chemicals, EDC, chemical mixtures, mesenchymal stem cells, adipogenesis, DNA methylation, low birth weight

## Abstract

Endocrine Disrupting Chemicals (EDCs) are man-made compounds that alter functions of the endocrine system. Environmental mixtures of EDCs might have adverse effects on human health, even though their individual concentrations are below regulatory levels of concerns. However, studies identifying and experimentally testing adverse effects of real-life mixtures are scarce. In this study, we aimed at evaluating an epidemiologically identified EDC mixture in an experimental setting to delineate its cellular and epigenetic effects. The mixture was established using data from the Swedish Environmental Longitudinal Mother and child Asthma and allergy (SELMA) study where it was associated with lower birth weight, an early marker for prenatal metabolic programming. This mixture was then tested for its ability to change metabolic programming of human mesenchymal stem cells. In these cells, we assessed if the mixture induced adipogenesis and genome-wide DNA methylation changes. The mixture increased lipid droplet accumulation already at concentrations corresponding to levels measured in the pregnant women of the SELMA study. Furthermore, we identified differentially methylated regions in genes important for adipogenesis and thermogenesis. This study shows that a mixture reflecting human real-life exposure can induce molecular and cellular changes during development that could underlie adverse outcomes.

## 1. Introduction

Human exposure is omnipresent to chemicals that interfere with the endocrine system; for example, via a variety of consumer products such as clothing, electronics, personal care products, and building material, as well as foods and food-contact materials [1,2]. Numerous studies worldwide have shown the presence of chemicals with proven or suspected endocrine disrupting properties, i.e., Endocrine Disrupting Chemicals (EDCs), in human blood and urine [3,4]. Exposure to EDCs has been associated with, among others, interference with sexual, neurological, and metabolic development in humans and animals [5].

EDC exposure during critical periods of fetal life is of particular concern for growth and metabolic development, as hormonal signaling plays a vital part in, e.g., stem cell differentiation and adipose tissue development [5,6,7,8,9,10]. The first neonatal measurement of the quality of the foetal growth is birth weight, where low birth weight can indicate foetal growth retardation in the intrauterine environment [11,12,13]. Numerous epidemiological studies have observed an association between low birth weight—when controlling for gestational age—and several diseases that manifest later in life such as obesity, type II diabetes, and cardiovascular diseases [14,15,16,17]. We recently reported an inverse association between prenatal exposure to a mixture of five perfluorinated alkyl substances (PFAS) and birth weight of children in the Swedish Environmental Longitudinal, Mother and Child, Asthma and allergy (SELMA) study [18]. This is supported by other studies that have found an inverse association between prenatal exposure to all of the chemicals from this PFAS mixture, as well as between the phthalate mono(2-ethyl-5-hydroxyhexyl) (MEHHP), the organochlorine dichlorodiphenyldichloroethylene (*p*,*p*′-DDE) and birth weight [19,20,21]. Additionally, a meta-analysis of 13 European mother-child cohorts found that a maternal occupational exposure to one or more EDC groups, including pesticides, phthalates and organic solvents, resulted in a higher risk of low birth weight of the child [22].

Lower birth weight is indeed an indicator for metabolic changes later in life and is supported by studies demonstrating associations between developmental EDC exposure and metabolic outcomes in children (reviewed, e.g., by Ghassabian et al. [23]). In particular, PFAS have been associated with child adiposity in several cohort studies [24,25,26,27]. In the SELMA study, changes in weight trajectories were associated with prenatal exposure to a mixture of EDCs, including PFAS, Triclosan, phthalates, non-phthalate plasticizers, bisphenols, polycyclic aromatic hydrocarbons, pesticides, and polychlorinated biphenyls (PCBs). Huselmman data is supported by ample experimental evidence for changes in metabolic programming induced by developmental exposure to EDCs (reviewed, e.g., by Howard [28]). This combined evidence implicates a potential for later life adverse health effects as a result of adverse changes to metabolic programming induced by EDC exposure during critical periods of development [29,30,31].

Epigenetic processes play an important role during development, where they direct cell differentiation, as well as tissue and organ development [32,33,34]. Epigenetics refers to heritable gene expression traits that do not entail DNA sequence alterations [35]. One of the key epigenetic processes is DNA methylation. DNA methylation refers to the addition of a methyl group to the 5-cytosine residue, which can alter, for example, the accessibility of transcription factor binding sites, thereby regulating gene expression. Environmental insults, such as EDC exposure, have been shown to induce epigenetic changes [36]. As epigenetic alterations can be mitotically heritable, and thus persistent over a long period, they might, at least partly, underlie the link between developmental EDC exposure and an individual’s susceptibility to metabolic disorders later in life [37,38,39,40].

While evidence for harmful effects of single EDCs is accumulating, the fact that human real-life exposures entail complex mixtures of chemicals is largely ignored. Most research studies, as well as risk assessment and chemical regulations, focus on single EDCs in isolation or groups of EDCs from the same chemical class, not reflecting the real-life situation. As a result, effects on human health may be underestimated if the chemical in question is present with several other chemicals that may contribute to the same adverse outcome. Thus, even when individual concentrations of chemicals are below their regulatory thresholds, their simultaneous exposure effects may produce long-lasting health adversities [4,41,42,43].

One challenge with regard to chemical mixtures is to identify the truly harmful ones among the infinite number of possible mixtures which humans are exposed to. To address this issue, we have previously, in a population-based epidemiological setting, established a whole mixture approach to define human relevant chemical mixtures in pregnant women associated with health outcomes in their children [44].

These mixtures have been further studied in experimental settings [45,46,47]. In the present study, we evaluated the effects of an EDC mixture associated with lower birth weight (Mix G1) on adipogenesis and DNA methylation patterns in human mesenchymal stem cells (hMSCs). The aim of this approach was to establish correlative evidence for the associations observed in the human data, and to delineate molecular events underlying these effects.

## 2. Results

### 2.1. Exposure to Human Relevant Concentrations of Mix G1 Induces Adipogenesis in hMSCs

**Figure 1:**
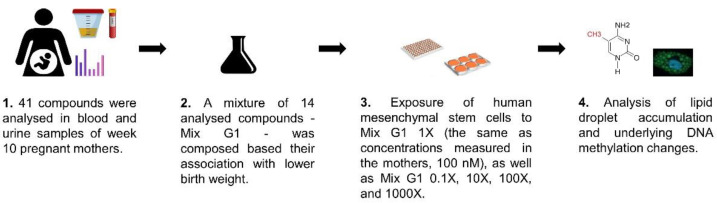
Overview of the steps in the study. **1**. In the SELMA pregnancy cohort blood and urine samples were collected around pregnancy week 10 for analysis of 41 compounds. A mixture of 14 analysed compounds was associated with lower birth weight. **2**. A 1 M stock of this epidemiologically identified EDC mixture (Mix G1), based on the levels and ratios measured in the SELMA samples, was composed in dimethyl sulfoxide (DMSO). **3**. In vitro studies were conducted where human mesenchymal stem cells were exposed to Mix G1 at 10 nM (Mix G1 0.1X), 100 nM (Mix G1 1X, corresponding to a typical human level based on SELMA data), 1 µM (Mix G1 10X), 10 µM (Mix G1 100X), 100 µM (Mix G1 1000X) in a 3-week long differentiation protocol. **4**. Morphological endpoints (adipogenesis measured as lipid droplet accumulation using high content imaging) and underlying epigenetic regulation (Illumina EPIC DNA methylation analysis) were assessed.

An epidemiologically defined chemical mixture associated with lower birth weight (Mix G1, composition shown in Table 1) was investigated for its effect on adipogenesis of hMSCs (Figure 1). hMSC differentiation into adipocytes captures an essential part of human adipose tissue development occurring during gestational weeks 14–16 [48,49]. To investigate if Mix G1 induces changes to the adipogenesis potential of hMSCs, bone-marrow derived hMSCs from two donors were exposed to five concentrations of Mix G1 for 3 weeks. Adipogenesis was then measured as lipid droplet accumulation by high content imaging.

**Figure 2:**
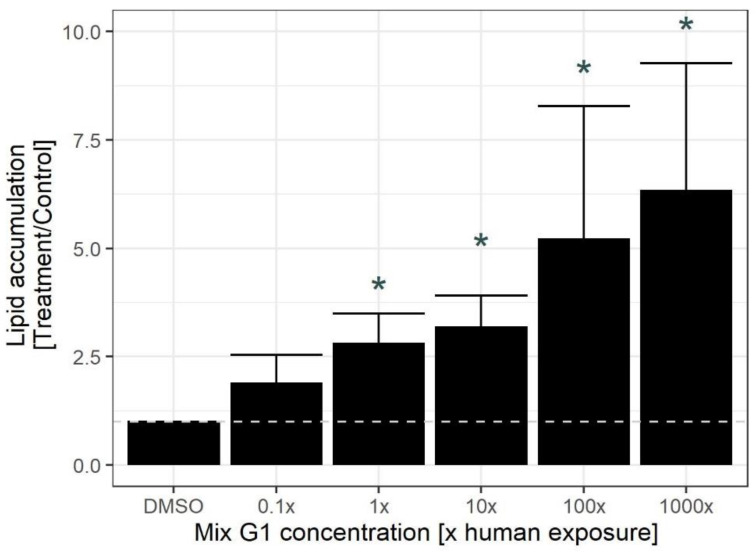
Mix G1-induced lipid droplet accumulation in hMSCs. hMSCs were exposed to 0.1X, 1X, 10X, 100X, 1000X Mix G1 or DMSO for three weeks. Lipids and nuclei were then stained using Bodipy 493/503 and Hoechst 33342, respectively, which were quantified using high content imaging. For each treatment, the lipid accumulation per cell is presented normalized to the lipid accumulation per cell of the DMSO control on the same plate, where mean and standard error per treatment is shown in the graph. * = *p* < 0.05 obtained by Kruskal–Wallis rank sum test followed by Dunns post hoc test adjusted with the Benjamini-Hochberg method when comparing with DMSO as a control.

As seen in Figure 2, Mix G1 increased lipid droplet accumulation in hMSCs. This increase was significant at the 1X concentration (i.e., the same levels as the serum mean concentration measured in the SELMA women) and at higher concentrations of Mix G1 (Figure 2). These results suggest that Mix G1, identified to be associated with lower birth weight in epidemiological data, induces changes to the adipogenic potential of hMSCs already at relevant human levels.

### 2.2. Exposure to Human Relevant Concentrations of Mix G1 Induces Altered DNA Methylation Profiles

**Figure 3:**
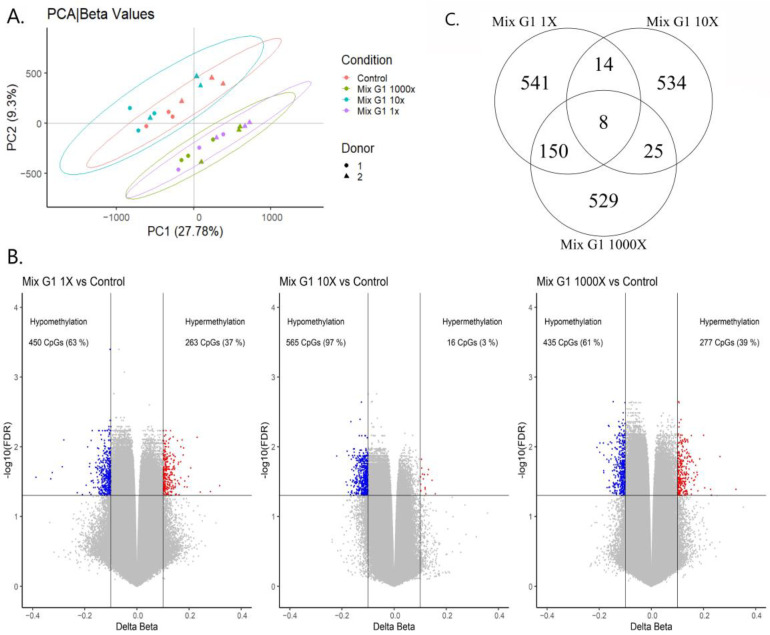
Characteristics of DMPs in hMSCs exposed to three different contractions of Mix G1 (1X, 10X, 1000X) and control (DMSO). (**A**) Principal component analysis of methylation states of over 850,000 CpGs. hMSCs from Donor 1 are represented as circles; hMSCs from Donor 2 are represented as triangles. Color indicates exposure where purple = Mix G1 1X (i.e., the same as concentrations measured in the SELMA study, 100 nM); blue = 10X; green = 1000X; and red = control (1:1000 DMSO). (**B**) Volcano plot showing −log10 (FDR) and beta difference compared to the control. DMPs are highlighted in red and blue colors based on horizontal lines indicating threshold for significance (FDR = 0.05) and vertical lines indicate threshold for change in methylation (−/+ 10% Δβ compared to DMSO control). (**C**) Venn Diagram showing overlapping DMPs among the treatments.

To investigate if Mix G1 induces epigenetic changes in hMSCs that could underlie the observed alterations in differentiation, genome-wide DNA methylation patterns were assessed after three weeks’ exposure to 1X, 10X, 1000X Mix G1 and DMSO using Illumina Infinium MethylationEPIC BeadChip array. Principal component analysis (PCA) showed that methylation patterns of hMSCs exposed to the lowest (1X) and the highest (1000X) concentrations were similar, and clearly different from the control and 10X Mix G1, indicating a possible non-monotonic effect on methylation (Figure 3A).

The largest number of differentially methylated positions (DMPs, FDR < 0.05, Δβ > 10%) were identified in hMSCs exposed to 1X Mix G1 (*n* = 713) (Table S3), followed by the exposure to 1000X (*n* = 712) (Table S3), and the least number of DMPs after exposure to 10X (*n* = 581) (Table S3) (Figure 3B). For all three treatments, the majority of DMPs were hypomethylated. The DMP with the largest methylation change was hypomethylated (−38.7%; FDR = 0.027) in comparison to the control and located in the *PUM1* gene after exposure to 1X concentration. The *PUM1* gene is coding for a protein which is known to be involved in hMSCs self-maintenance and proliferation.

As shown in Figure 3C, only 8 identified DMPs were shared among the three treatments. These 8 DMPs were hypomethylated for all treatments, and 4 of them were located in gene regions (*CAPN8*, *NR6A1*, *SERINC2*, *C5orf66*). Methylation levels of these overlapping DMPs were not affected in a non-monotonic fashion by the mixture but showed rather a threshold response (Figure S1).

Most DMPs (*n* = 158) were shared between the hMSCs exposed to the 1X and the 1000X Mix G1 (Table S4). These included hypermethylated DMP annotated to the *FGF9*, *MYOF*, *ZNRF3*, *ACSM3*, *AMN*, *APBB2*, *EGFL6*, *RBMS1*, *GNAQ*, *PTPRJ*, and *TOMM7* genes known to be important for osteogenesis and thermogenesis [50,51], adipogenesis [52], body fat distribution [53], fatty acid metabolism [54,55], obesity [56,57,58], glucose metabolism [59], type 2 diabetes and insulin signaling [60,61], respectively. Several DMPs were also hypomethylated in the *FFAR2*, *IRS-1*, *MAF*, and *PGM1* genes known to be important for lipid accumulation [62], adipocyte differentiation [63,64], and glycogen metabolism and adipogenesis [65,66], respectively.

To evaluate the distribution of DMPs induced by exposures to Mix G1, their location in relation to gene regions and CpG islands was plotted (Figure S2). Genomic distribution of the hypermethylated and hypomethylated DMPs in comparison to all the CpGs on the Illumina EPIC BeadChip showed enrichment mainly in intergenic regions (IGR), open sea, and shelf regions, while depletion was mainly in CpG island, shore, and 1st Exon regions.

In summary, exposure to all three concentrations of Mix G1 induced significant DNA methylation changes with more hypo- than hypermethylated DMPs. Most overlap was found to have the lowest and highest concentrations.

### 2.3. Differentially Methylated Regions Induced by Mix G1 Treatment Are Linked to Adipogenesis and Metabolic Functions

**Figure 4:**
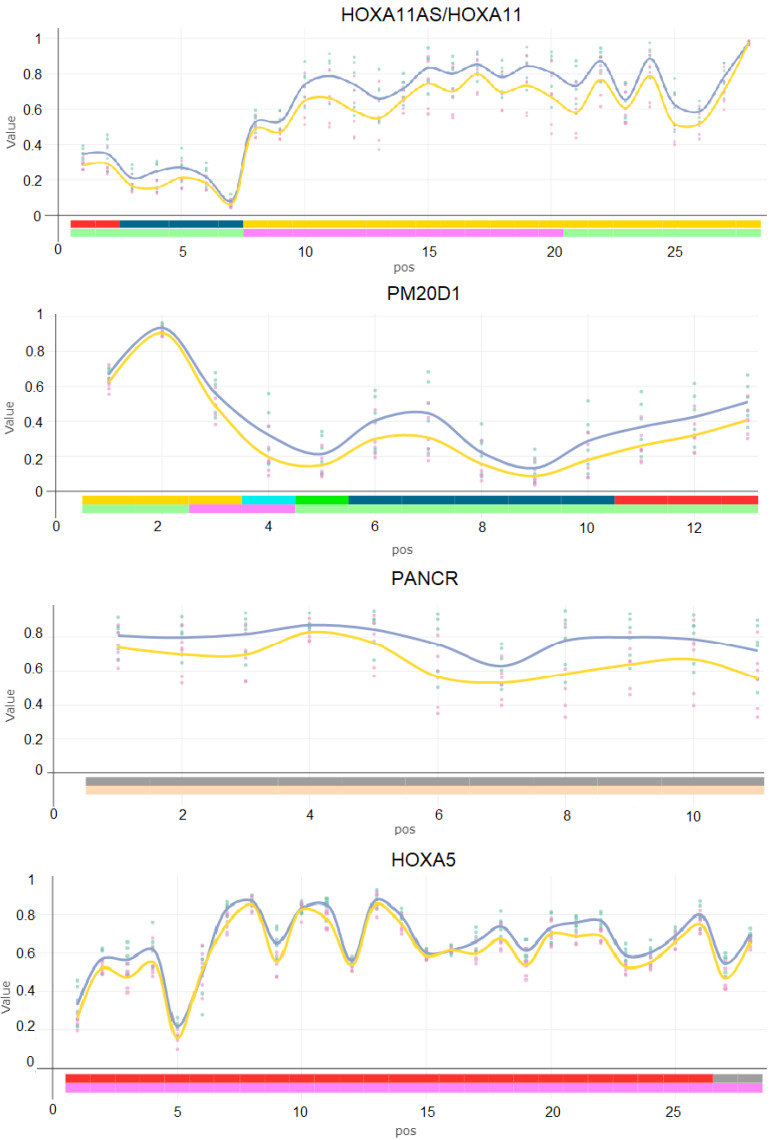

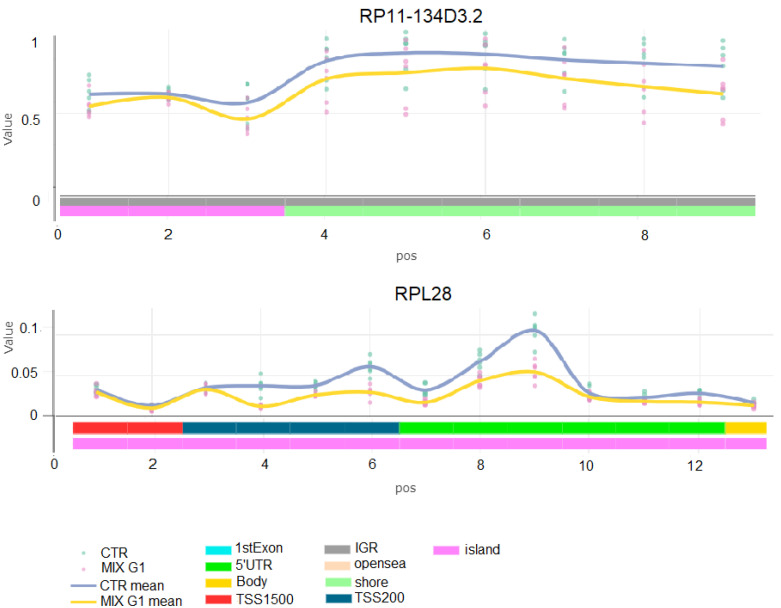
Identified differentially methylated regions (DMRs) upon exposure to Mix G1 1X. The yellow line indicates the mean methylation value upon exposure to Mix G1 1X, while the blue line indicates the mean methylation value upon exposure to control. DMR analysis was conducted using the *Bumphunter* method embedded in the ChAMP analysis package. The plots show beta methylation values (*Y* axis) of each consecutive CpG site (*X* axis) within each DMR region (not their real genomic location).

As the role for methylation at single CpGs, in particular in intergenic regions, is unclear, we focused our next analysis on identification of differentially methylated regions (DMRs). DMRs are composed of several, often correlated neigbouring DMPs in a specific gene region. As a result, DMRs are more likely to have biological relevance and associations with changes in gene expression than DMPs. Among the freely available tools, Bumphunter is a long-established tool for detection of DMRs. Thus, we conducted Bumphunter analysis for identification of DMRs (family-wise error rate (FWER) < 0.20) that are composed of seven or more successive CpGs affected by Mix G1 with a maximum distance of <300 bp between each other. Six DMRs, annotated to the genes *HOXA11AS/HOXA11*, *PM20D1*, *PANCR*, *HOXA5*, *RP11-134D3.2*, and *RPL28*, were identified to be hypomethylated by Mix G1 1X exposure (FWER < 0.20) (Table 2 and Table S5, Figure 4). No DMRs were identified upon exposure to Mix G1 10X. Upon exposure to Mix G1 1000X, two hypomethylated DMRs were identified, which were in the same regions of the *HOXA11AS/HOXA11* and *PANCR* genes as for Mix G1 1X (Figure S3 and Table S6). Expression of *HOXA11AS* and *HOXA5* positively regulates adipocyte differentiations [67,68]. While *PM20D1* codes for the enzyme PM20D1, a newly identified regulator of thermogenesis and glucose homeostasis [69,70]. Lastly, expression of *RPL28* gene has previously been identified to have a negative correlation with BMI [71].

Thus, exposure of hMSCs to 1X and 1000X Mix G1 induced DNA methylation changes in regions linked to genes that play a role in adipogenesis and metabolic functions.

### 2.4. Mix G1-Induced DMRs Are Enriched at Genes Linked to Metabolism Related Pathways

To explore which pathways may be affected by Mix G1 1X induced DMRs, enrichment of gene ontology (GO) categories was performed. To increase the number of included DMR-associated genes, adj. *p* value < 0.05 was used. We observed significant enrichment for three major GO categories: biological process (BP) (*n* = 46), cellular component (CC) (*n* = 14), and molecular function (MF) (*n* = 3) (Table S7).

**Figure 5:**
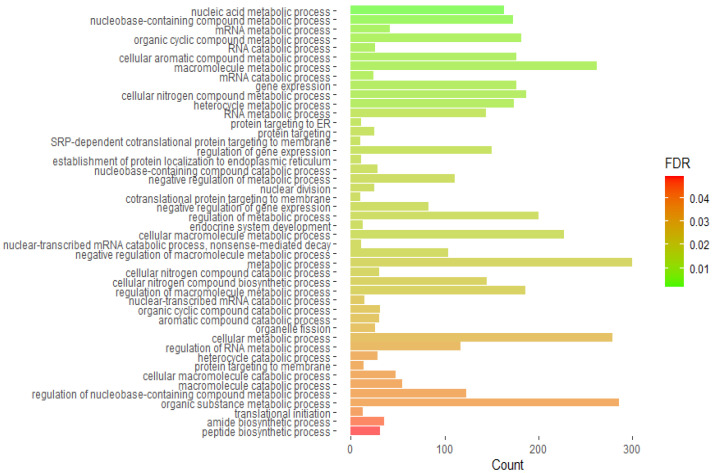
Gene ontology analysis on the list of genes that contain differentially methylated regions showing the biological terms. Count indicates the number of genes enriched in a pathway; FDR indicates False Discovery Rate adjusted *p* value.

The enrichment results for the BP category demonstrated that Mix G1 1X altered methylation of genes whose products are mainly involved in metabolic processes in the cell on the level of nucleic acids (e.g., nucleic acid metabolic process and mRNA metabolic process), gene expression (e.g., gene expression and regulation of gene expression), and proteins (e.g., protein targeting to ER). Additionally, endocrine system development and cellular macromolecule metabolic processes were enriched (Figure 5).

## 3. Discussion

In this study, we tested a mixture of EDCs associated with lower birth weight for cellular and molecular effects in vitro. The mixture, Mix G1, consists of 14 analysed compounds from seven chemical classes. We report, for the first time, that this chemical mixture at levels found in the SELMA mothers can induce adipogenesis and alter DNA methylation in genes important for metabolic functions. While we attempted to relate the ratios of the different components as closely as possible to real-life human exposure, we cannot exclude those different chemical properties affected the actual concentration levels within the cells.

In vitro exposure to Mix G1, already at human relevant concentrations, significantly induced adipogenesis of hMSCs in comparison to control treatment. We observed this when combining hMSCs from two different donors, suggesting that this is not an individual-specific effect. Our results are in accordance with a study by Mentor et al. (2020) [45] that reported significant lipid accumulation in zebrafish upon developmental exposure to a similar EDC mixture. In addition, several other studies have shown promotion of adipogenesis upon exposure to single EDCs included in Mix G1. For example, 1 µM of DDE increased adipogenesis in adipose derived MSC and 3T3-L1 adipocytes [72,73], as did 1 µM of MEHP in bone marrow stromal cells, and 10 µM in primary mouse bone marrow culture and 3T3-L1 adipocytes [74,75,76]. Several fold higher concentrations of MBzP (100 µM) and MINCH (50 µM) [77,78] have promoted adipogenesis in rat primary stromal vascular fraction of adipose tissue and in 3T3-L1 cells, where a similar result was observed upon exposure to PFOA (40 µM), PFHxS (80 µM), and PFOS (200 µM) [79,80].

Contradicting results have been reported for exposure to triclosan with anti-adipogenic effects on hMSCs at 0.156 to 2.5 μM and in 3T3-L1 adipocytes at 50 µM [81,82], while an in vivo high fat diet with 0.35 mM of triclosan resulted in mice with larger abdominal white adipose tissue [83]. It is evident that most previous studies on single chemicals found in Mix G1 have observed effects at µM concentrations, while Mix G1 showed effects already at 100 nM, and thus the concentrations of the individual compounds were several folds lower, indicating that the mixture has more potent effects than the single chemicals.

During adipogenesis, bone-marrow derived MSCs develop into adipocytes, and this process is regulated by epigenetic processes, such as changes in DNA methylation patterns at specific genes [84,85]. In this study, we showed that Mix G1 induced significant DNA methylation changes at 713 positions already at a relevant human level (1X). The majority of these DMPs were hypomethylated (63%) in IGR. While the significance of changes in CpG islands for gene expression are well known, the role of the intergenic DNA methylation is not well understood, yet these regions may contain enhancer regulatory sequences or noncoding transcripts or enhancer RNA [86,87]. This finding is in accordance with a study conducted by van den Dungen and colleagues (2017) [88] where hMSCs exposed to 10 μM PFOS also resulted in more hypomethylated DMPs in the gene body and IGR, although their results were not significant after FDR correction. Interestingly, despite a several fold lower PFOS concentration in Mix G1 1X (9.7 nM), our study identified many DMPs, again suggesting a higher potency of mixture effects in comparison to single chemical effects. A possible mechanism that could explain enrichment of hypomethylated DMPs by Mix G1 could entail interference with nuclear receptors such as the oestrogen receptor.

Oestrogen receptor beta (ERβ) has been shown to regulate DNA methylation patterns at specific sites, whereby ERβ deficiency also showed more hypo- than hypermethylated positions [89]. As an underlying mechanism, Liu and colleagues (2016) [89] proposed recruitment of thymine DNA glycosylase (TDG), an enzyme involved in DNA demethylation [90], to regulatory regions of ERβ target genes. As TDG has been shown to interact with a number of nuclear receptors [91], this could apply to other receptors affected by Mix G1 compounds as well.

We also observed that Mix G1 10X induced a noticeably different effect than Mix G1 1X and 1000X, indicating a possible non-monotonic response. As a result of structural similarities of the EDCs to endogenous hormones, they are well known to be able to induce a response at low doses, as well as exhibit non-monotonic dose-response curves [92,93]. However, no non-monotonic response was observed for the 8 commonly shared DMPs among the treatments, nor in the lipid droplet accumulation assay. Thus, we cannot draw a definite conclusion on whether or not Mix G1 produced non-monotonic effects on the DNA-methylome in hMSCs.

Six hypomethylated DMRs mapped to six genes were identified upon exposure to Mix G1 1X. Remarkably, four of these genes are linked to adipogenesis, adipose tissue function, birthweight, or obesity [67,68,69,70,71,94,95]. The most significant DMRs overlap with the promoter region of the *PM20D1* (peptidase M20 domain containing 1) gene. This gene encodes a biosynthetic enzyme that mediates UCP1-independent uncoupling of mitochondrial respiration, driving thermogenesis of brown and beige fat.

*PM20D1* is positively regulated in adipocytes by the master regulator of adipogenesis, peroxisome proliferator activated receptor-γ nuclear receptor (PPARγ) [69,96]. Recent studies have also shown that methylation of the promoter region of *PM20D1* is inversely correlated with its expression in human frontal cortex brain samples [97,98,99], suggesting that changes in DNA methylation at this region can lead to functional alterations in gene expression.

Furthermore, our analysis identified hypomethylation in a DMR that overlapped with promoter regions of *HOXA11* and its anti-sense non-coding RNA *HOXA11-AS*, as well as a DMR in the *HOXA5* gene. These genes have been shown to have an inverse correlation between their promoter methylation and expression, and they have well-established roles in embryonic development in general, and MSC differentiation in particular [68,100,101,102]. For example, knockdown of *HOXA11-AS1* in human adipose-derived stem cells inhibited adipogenesis, and high expression was found in obese patients in comparison to the controls, while low RNA expression was found to be associated with lower birth weight and lower gestational age in humans [67,103]. Similarly, a positive correlation between *HOXA5* expression and adipogenesis was shown in primary mouse adipocytes, but, contrastingly, low expression was found in adipose tissue of obese mice in comparison to the controls [68,101].

We identified two other DMRs in genes *RPL28* and *PANCR* that have not previously been associated with adipogenesis or lower birth weight before but have appeared in several obesity or metabolism related studies. *RPL28* is a gene coding for 60S ribosomal protein L28, and its promoter region, 234 bp away from the DMR we have identified, has been shown to be significantly hypomethylated in obese individuals’ blood samples [104]. Furthermore, its expression in subcutaneous white adipose tissue has been negatively associated with BMI in overweight human subjects [71]. While *PANCR*, an intergenic long noncoding RNA, positively regulates expression of adjacent the *PITX2* gene during cardiomyocyte differentiation [94]. Additionally, *PITX2* is also important for the development of myocardial components implicated in the pathogenesis of atrial fibrillation, for which low birth weight infants have increased risk of developing later in life [105,106,107].

Finally, results from the gene ontology analysis were almost exclusively related to molecular and cellular metabolism (Figure 5). Additionally, pathways implicated in processes on organismal level such as endocrine system development and regulation of metabolic processes were enriched. Examples of the identified pathways on the cellular level are establishment of protein localization to endoplasmic reticulum (ER) and protein targeting to ER.

Since stress in ER has been previously linked to adipogenesis and obesity [108], altered DNA methylation in genes involved in the process of transferring proteins to the ER could potentially play a role in adipogenesis.

This study has some limitations. Firstly, we used primary hMSCs from two donors, which limits donor-specific responses to a certain extent. For even more generalisable effects, it would have been beneficial to include hMSCs from additional donors. Furthermore, while differentiation of hMSCs reflects an essential part of gestational development of adipose tissue, it is not by any means exhaustive for studying effects of Mix G1 on metabolic programming. Additionally, we have not quantified the intracellular concentration of Mix G1; thus, the levels and ratios within the cells might deviate from the calculated ones. Also, we did not include Mix G1 100X in the Illumina EPIC DNA methylation analysis, due to limited resources. An additional concentration would have helped to better understand dose-responses of the DNA methylation changes. Lastly, due to limited material, we did not address if the observed DNA methylation changes are correlated with alterations in expression of the respective genes. While this would have added information on the functional implications of our findings, it is of note that epigenetic changes do not necessarily translate directly into transcriptional alterations, but might instead affect, e.g., inducibility of the gene by a later stimulus [109].

## 4. Materials and Methods

### 4.1. Identification and Preparation of Mix G1

The chemical mixture (Mix G1) used experimentally in this study was designed within the EU project EDC-MixRisk (http://edcmixrisk.ki.se/, accessed on 19 February 2022). Levels of 54 compounds were measured in blood and urine of >2300 pregnant women in median gestational week 10 included in the Swedish Environmental Longitudinal, Mother and Child, Asthma and allergy (SELMA) study [110]. Forty-one compounds (corresponding to 26 parent compounds), out of the 54 measured, showed levels above the limit of quantification in more than half of the women, and were therefore included in the statistical analyses. The mixture identification includes three steps, also described in Bornehag et al. (2019) and Tables S1 and S2 [44] and in the supplementary material. Firstly, we identified chemicals of concern measured in urine and serum of the SELMA mothers that were associated with a lower birth weight in their children. Such chemicals of concern were selected using weighted quantile sum regression, which is a strategy for estimating empirical weights for a weighted sum of quantiled concentrations (e.g., quartile or decile scores) most associated with the health outcome [111]. The results of this analysis are shown in Tables S1 and S2. Secondly, we estimated the serum levels of the chemicals of concern. Urine compounds were converted into serum concentrations through estimation of daily intake [112,113] and chemicals measured in serum were used as such, as described in Bornehag et al. (2019). Thirdly, the mixing proportions of the chemicals of concern were established using serum mean levels from the SELMA mothers. These estimations followed a simplified equation of a one-compartment toxicokinetic model [113]. This procedure resulted in fourteen chemicals of concern that were included in Mix G1.

The components of Mix G1 and their individual concentrations are listed in Table 1. One molar (1 M) solution in DMSO (Sigma-Aldrich, Saint Louis, MO, USA) was prepared. In the experimental systems, this mixture was tested using concentrations corresponding to human exposure, where 1X denotes the serum mean of exposure levels in SELMA pregnant women.

### 4.2. Cell Culture

Bone marrow-derived hMSCs from 2 donors (one male and one female) were kindly provided by Dr. Katarina Leblanc (Karolinska Institutet, Stockholm, Sweden). Two donors were used to decrease the risk of donor-specific findings, and the results from the two donors were not separated in the statistical analyses. The cells were cultured in growth media consisting of DMEM (Gibco, Waltham, MA, USA) supplemented with 10% Fetal Bovine Serum (FBS; Gibco, Waltham, MA, USA), 1% penicillin-streptomycin (Gibco, Waltham, MA, USA), and 2% l-glutamine (Gibco, Waltham, MA, USA) at 37 °C in an incubator with 5% (*v*/*v*) CO_2_.

The hMSCs were seeded in black-walled 96 well plates with µCLEAR bottom (Greiner Bio One, Kremsmünster, Austria) or 6 well-plates (VWR→734-2323). Growth media was replaced by treatment media (TM) consisting of DMEM supplemented with 10% charcoal stripped FBS (DCC, Gibco, Waltham, MA, USA), 1% penicillin-streptomycin (Gibco, Waltham, MA, USA), and 2% l-glutamine (Gibco, Waltham, MA, USA) two-three days before treatment start (day 0). On day 0, the treatments started and continued for 21 days. The Mix G1 exposure media (EM) consisted of TM supplemented with Mix G1 in DMSO at 10 nM (0.1X), 100 nM (1X), 1 µM (10X), 10 µM (100X), and 100 µM (1000X). Control medium (CM) contained TM supplemented with DMSO in a ratio of 1:1000. As positive control, adipogenic induction media (AIM), consisting of TM supplemented with 1 µg/mL insulin (Sigma-Aldrich, Saint Louis, MO, USA), 0.25 uM dexamethasone (Sigma-Aldrich, Saint Louis, MO, USA) and 0.5 mM 3-isobutyl-1-methylxanthine (IBMX, Sigma-Aldrich, Saint Louis, MO, USA), was used. EM/CM/AIM were changed twice a week.

AIM induced adipogenesis (measured as lipid droplet accumulation) in all experiments for both donors.

### 4.3. Lipid Droplet Accumulation

Differentiation of hMSCs into adipocytes was quantified by fluorescent microscopy, as previously described [114]. Briefly, media from the cultured cells in 96 well plates was replaced by 100 µL media containing 10 µg/mL BODIPY 493/503 (Gibco^®^, Waltham, MA, USA) and 2 µg/mL of Hoechst 33,342 (Gibco^®^, Waltham, MA, USA) and incubated for 1 h at 37 °C, 5% CO_2_. The cells were then washed three times with DPBS with Ca and Mg (Gibco, Waltham, MA, USA), after which images were immediately taken in FITC and DAPI channels at 10X magnification, at 16 sites per well, using the Image Xpress Micro High-Content Analysis System. Images were further analyzed with the MetaXpress High-Content Image Acquisition and Analysis software (Molecular Devices, Sunnyvale, CA, USA). Using the Transfluor HT analysis module, lipid droplets were quantified by measuring the integrated granule intensity, and this value was normalized to nuclei count. For each treatment condition, the lipid accumulation per cell is presented as a ratio compared to the lipid accumulation per cell of the DMSO control on the same plate.

### 4.4. DNA Extraction and Genome-Wide DNA Methylation Analysis

The DNA was extracted from hMSCs cultured in 6-well plates using AllPrep DNA/RNA micro kit (Qiagen, Hilden, Germany), according to the manufacturer’s protocol. Briefly, the cultured cells were lysed with a lysis buffer from BioRad (Aurum Total RNA Lysis Solution), lysate was then frozen in −80 °C. When thawed, 250 µL of RLT buffer was added and the lysate was transferred to a spin column, washed and centrifuged several times according to protocol. DNA was eluted with EB buffer. The Illumina EPIC Array analysis was conducted by the core facility for Bioinformatics and Expression Analysis (BEA) (Karolinska Institutet, Stockholm, Sweden).

The data was normalized and pre-processed by BEA using Chip Analysis Methylation Pipeline (ChAMP) where quality control and batch correction for slide and array were performed. The distribution of DMPs were determined against the base genome annotation for Illumina’s EPIC methylation array (Homo sapiens genome assembly GRCh37 (hg19)). The degree of methylation at a CpG site (beta-value (*β*)) was calculated using Formula (1), where the ratio of methylated fluorescent probe intensity against a total probe intensity signal was calculated with a constant offset of 100 [115,116]. The beta value is always between 0 and 1, where 1 indicates that a CpG site is 100% methylated and 0 indicates 0% methylation. The Delta Beta value is defined as the difference between the average beta values of the treatment (Mix G1) and the control samples (Formula (2)). Therefore, a negative Delta Beta value refers to the treatment sample being less methylated than control, hypomethylated, and positive Delta Beta value refers to the treatment sample being more methylated than control, hypermethylated.

β=Methylated probe intensity÷(Unmethylated probe intensity+Methylated probe intensity+100)

Formula (1). Calculation of the beta-value (*β*) in the Illumina EPIC Array.

(1) $$\begin{array}{c} Deltabeta\\ =Average\;beta\;value\;of\;Mix\;G1\;samples\\ -Average\;beta\;value\;control\;samples \end{array}$$

Formula (2). Calculation of the Delta Beta value.

The *p*-value was adjusted for multiple testing using false discovery rate (FDR) and differentially methylated CpG positions (DMPs) were identified based on differences in DNA methylation means between the treatment groups (Mix G1 1X, 10X and 1000X) and control (DMSO) group (absolute value of Δ*β* ≥ 0.1 and FDR < 0.05).

To investigate how the different samples clustered, a principle component analysis (PCA) was conducted using the singular value decomposition function (\textit{prcomp}) in R, version 4.0.5; the distribution of DMPs and the volcano plots were also conducted in R, version 4.0.5, using the base genome annotation for Illumina’s EPIC methylation array (Homo sapiens genome assembly GRCh37 (hg19)) and ggplot2 [117] package, respectively.

To investigate methylation changes at a region level, the Bumphunter tool was employed where an algorithm uses “peak detection” method for DMRs identification [117]. In short, regression coefficients of adjacent CpGs in a region are smoothed and plotted in a curve. Potential “peaks” or DMRs are then identified as regions where the collection of these coefficients are higher than expected by chance [118,119,120]. The Bumphunter (champ.DMR) function from the ChAMP package was used for DMR analysis with its default setting for DMR composition of seven or more successive DMPs within a 300-base pair genomic region. The DMRs with Family Wise Error Rate <0.2 were considered differentially methylated. Genes were extracted from the DMR analysis with adjusted *p* value cut off by 0.05 and used in a testing for enrichment of gene ontology (GO) categories from the GO.db annotation package, using functions called “GOmeth” and “champ.GSEA” with default settings. Both DMR and GO analysis were conducted on combined data from both donors, and to account for sex bias, sex chromosomes were excluded.

## 5. Conclusions

In conclusion, our study showed that a human relevant EDC mixture of 14 analysed compounds induced lipid droplet accumulation of hMSCs. Additionally, we found that exposure to the mixture leads to dysregulated DNA methylation patterns of genes previously associated with lower birth weight, obesity, as well as development and function of adipose tissue. Thus, this study suggests that a mixture corresponding to human real-life exposures, both in terms of proportions and concentrations, can change the metabolic set-point during development, which may underlie the epidemiological association between chemical mixture and lower birth weight. This, therefore, emphasizes the need for taking chemical mixture exposure into account, both in research and for regulatory risk and hazard assessment.

## Figures and Tables

**Table 1:** Individual chemicals within Mix G1.

Chemical Class	Parent Compound ^1^	Mix G1 Compound ^2^	Full Compound Name	Concentration (nM) ^3^	CAS Number
Phthalates	DEP	MEP	Monoethyl phthalate	29.7	2306-33-4
	DBP	MBP	Monobutyl phthalate	26.4	131-70-4
	BBzP	MBzP	Monobenzyl phthalate	5.3	2528-16-7
	DEHP	MEHP	Mono-(2-ethylhexyl) phthalate	19.0	4376-20-9
Plasticizer	DiNCH	MINCH	2–4-Methyl-7-oxyooctyl-oxycarbonyl-cyclohexane carboxylic acid	0.5	1588520-62-0
	TTP	DPP	Diphenylphosphate	0.5	838-85-7
Antibacterial		Triclosan		0.3	3380-34-5
PAH		2OHPH	2-Hydroxyphenanthrene	1.3	605-55-0
Pesticide	Pyrethroids	3-PBA	3-Phenoxybenzoic acid	0.1	3739-38-6
PFAS		PFOA	Perfluorooctanoic acid	3.6	335-67-1
		PFOS	Perfluorooctane sulfonate	9.7	1763-23-1
		PFHxS	Perfluorohexane sulfonate	3.0	355-46-4
Organo-chlorine pesticide		HCB	Hexachlorobenzene	0.1	118-74-1
	DDT	*p*,*p*′ DDE	Dichlorodiphenyltrichloroethane	0.5	50-29-3

^1^ Parent compounds of the analytes that were measured in the SELMA women. ^2^ Analytes measured in the SELMA women; these compounds were used to compose Mix G1. ^3^ Concentration of chemicals is in relation to 100 nM (100%) total mixture concentration at 1X SELMA exposure, meaning that the mixture ratios of the chemicals are the same as the listed concentrations. e.g., MEP constituted 29.7% of the mixture.

**Table 2:** Details about the six identified DMRs upon exposure to Mix G1 1X.

Gene	CHR ^1^	Start ^2^	End ^3^	Length	Number of CpGs	FWER ^4^
*HOXA11AS/HOXA11*	7	27,224,700	27,226,329	1629	28	0.096
*PM20D1*	1	205,818,484	205,819,609	1125	13	0.096
*PANCR*	16	87,101,534	87,102,691	1157	11	0.12
*HOXA5*	7	27,183,643	27,184,853	1210	28	0.144
*RP11-134D3.2*	4	111,532,996	111,533,951	955	9	0.152
*RPL28*	19	55,896,842	55,897,819	977	13	0.152

^1^ CHR = chromosome. ^2^ Start = the start of the DMR region in the Human (GRCh37/hg19) Assembly genome. ^3^ End = the end of the DMR region in the Human (GRCh37/hg19) Assembly genome. ^4^ FWER = family wise error rate.

## Data Availability

The data discussed in this publication have been deposited in NCBI’s Gene Expression Omnibus and are accessible through GEO Series accession number GSE196046.

## Supplementary Materials

The following supporting information can be downloaded at: https://www.mdpi.com/article/10.3390/ijms23042320/s1.
