# Supplementary material for: A Mixture of Endocrine Disrupting Chemicals Associated with Lower Birth Weight in Children Induces Adipogenesis and DNA Methylation Changes in Human Mesenchymal Stem Cells
Source: Int J Mol Sci. 2022 Feb 19;23(4):2320. doi: 10.3390/ijms23042320 (PMC8879125; doi:10.3390/ijms23042320)
Supplement: Supplementary file 1 [file ijms-23-02320-s001.zip › Supplementary Information Tables S3-4.pdf]

Table S3. Differentially methylated positions upon exposure of hMSCs to Mix G1 1X, 10X, and 1000X.

Illumina ID	FDR_Mix_G1_1X	deltaBeta_Mix_G1_1X	FDR_Mix_G1_10X	deltaBeta_Mix_G1_10X	FDR_Mix_G1_1000X	deltaBeta_Mix_G1_1000X
cg00046991	0.02681	-0.1063	0.1645	-0.05663	0.3417	-0.03514
cg00077302	0.02655	-0.06864	0.01078	-0.1527	0.08407	-0.05954
cg00104059	0.8152	-0.008365	0.0187	-0.1025	0.374	-0.03513
cg00106685	0.007999	-0.1009	0.108	-0.04861	0.01665	-0.07171
cg00115864	0.6765	-0.01328	0.01972	-0.1095	0.8187	0.008317
cg00121963	0.1815	0.07803	0.567	0.04076	0.04929	0.126
cg00135157	0.136	-0.03341	0.01416	-0.1165	0.1807	-0.03726
cg00145093	0.1487	0.07303	0.8503	0.01088	0.04941	0.1084
cg00152613	0.7471	0.01025	0.02237	-0.1376	0.06901	-0.07839
cg00189927	0.03427	-0.1033	0.2041	-0.05565	0.08504	-0.08183
cg00191710	0.369	0.03003	0.7198	0.01725	0.01514	0.109
cg00238438	0.02785	-0.1078	0.1281	-0.0656	0.1033	-0.06652
cg00252873	0.03329	-0.1185	0.5133	0.02578	0.5792	-0.02941
cg00253126	0.01564	0.143	0.634	-0.01977	0.06029	0.09752
cg00283792	0.1065	-0.06053	0.0146	-0.1223	0.06144	-0.06779
cg00292351	0.1712	0.06289	0.8393	-0.01093	0.02628	0.1095
cg00296819	0.05351	-0.051	0.01975	-0.1459	0.01672	-0.07769
cg00299454	0.005874	-0.1123	0.2234	-0.0285	0.00461	-0.1126
cg00304076	0.3042	-0.02674	0.01567	-0.1295	0.261	-0.03459
cg00308479	0.4466	0.03176	0.8246	-0.01188	0.01768	0.1013
cg00319595	0.03689	-0.117	0.3238	0.04415	0.08304	-0.08936
cg00321855	0.02496	-0.1215	0.4032	-0.04559	0.03459	-0.111
cg00328376	0.9321	0.003499	0.02605	-0.1028	0.5627	-0.02069
cg00329695	0.01909	-0.1248	0.6823	-0.01929	0.092	-0.05683
cg00333843	0.03732	-0.1273	0.9385	0.005637	0.04604	-0.1044
cg00343349	0.07586	-0.04736	0.03245	-0.08606	0.02022	-0.1049
cg00346984	0.2009	-0.04842	0.4602	-0.02632	0.01209	-0.1032
cg00351838	0.2662	-0.04266	0.5017	-0.03806	0.03535	-0.1263
cg00366537	0.06534	0.08413	0.7471	-0.01498	0.03009	0.1076
cg00376064	0.0386	-0.07664	0.9108	-0.003102	0.005224	-0.1196
cg00401085	0.02514	-0.1431	0.1342	-0.06604	0.06948	-0.07445
cg00434884	0.1725	-0.04879	0.6652	-0.01932	0.02068	-0.115
cg00435490	0.1418	0.07952	0.877	-0.01044	0.03154	0.1294
cg00456581	0.04518	-0.1132	0.1531	-0.07661	0.0419	-0.1217
cg00458868	0.01549	-0.1286	0.1374	-0.09606	0.006912	-0.1129
cg00463052	0.7362	-0.01231	0.02062	-0.1384	0.1661	-0.04656
cg00493363	0.1169	-0.04915	0.01906	-0.1135	0.3508	-0.02677
cg00506540	0.03676	-0.09153	0.5691	-0.02635	0.01069	-0.1351
cg00513665	0.07016	0.0612	0.1791	0.04171	0.01342	0.1013
cg00546932	0.7731	-0.0115	0.04849	-0.1013	0.6477	-0.01639
cg00564465	0.1485	-0.07594	0.2508	-0.05976	0.01407	-0.1398
cg00576650	0.6224	-0.013	0.02076	-0.1042	0.214	-0.03732
cg00599503	0.261	-0.04169	0.03381	-0.1159	0.6965	-0.0162
cg00620602	0.04123	-0.115	0.8477	-0.009751	0.0858	-0.08243
cg00695936	0.0381	0.1004	0.971	-0.002335	0.1618	0.04165
cg00712762	0.02221	-0.1057	0.03647	-0.09125	0.04398	-0.07677
cg00718242	0.582	0.0171	0.01959	-0.1247	0.1617	-0.04079
cg00735467	0.03848	-0.1216	0.5211	-0.02277	0.3677	-0.03931

cg00737548	0.08876	-0.09312	0.4128	-0.04454	0.02147	-0.1435
cg00743831	0.01912	-0.1328	0.1016	-0.093	0.02536	-0.1026
cg00747618	0.03318	-0.1018	0.3159	-0.03815	0.3966	-0.02679
cg00760931	0.01233	-0.1071	0.0188	-0.1166	0.008544	-0.1315
cg00764741	0.2925	-0.05659	0.8141	-0.01386	0.04508	-0.1044
cg00771217	0.08836	0.09245	0.7175	-0.02133	0.04284	0.1148
cg00826780	0.1562	-0.07619	0.9944	0.0004633	0.04752	-0.1188
cg00858624	0.02458	-0.1014	0.1083	-0.07724	0.03313	-0.1322
cg00876687	0.1047	-0.05985	0.01686	-0.1352	0.3472	-0.03398
cg00880288	0.07622	-0.06056	0.04728	-0.1013	0.04308	-0.06846
cg00889599	0.0409	0.09202	0.8227	0.01182	0.03314	0.1016
cg00893368	0.03247	-0.07605	0.302	-0.02959	0.01716	-0.1116
cg00921992	0.7088	-0.01318	0.04344	-0.1152	0.298	-0.0395
cg00923860	0.005906	-0.1214	0.5189	0.01817	0.01993	-0.07424
cg00927503	0.006686	-0.1064	0.2404	-0.02526	0.1051	-0.03589
cg00929332	0.05934	-0.1021	0.3404	-0.05371	0.03679	-0.1047
cg00934355	0.04608	-0.1055	0.7959	-0.01336	0.07497	-0.0866
cg00938087	0.1222	-0.0611	0.4192	-0.04688	0.02059	-0.1018
cg00977595	0.9699	-0.001519	0.03587	-0.1162	0.302	-0.06124
cg00988592	0.06632	-0.06178	0.1126	-0.07269	0.01844	-0.1052
cg01005535	0.02768	-0.08216	0.9373	0.003221	0.01057	-0.1114
cg01008680	0.5219	-0.02358	0.04556	-0.1085	0.3752	-0.03696
cg01009434	0.02101	0.08968	0.3528	0.0342	0.01584	0.1146
cg01054652	0.05523	0.1061	0.8168	0.01729	0.03654	0.1218
cg01061877	0.02142	0.1119	0.2792	0.04746	0.02517	0.1105
cg01063265	0.04124	0.1155	0.3714	-0.04036	0.176	0.05797
cg01078983	0.2875	-0.04121	0.02763	-0.1029	0.06067	-0.08973
cg01097488	0.04089	-0.1451	0.4431	-0.05142	0.05815	-0.131
cg01100030	0.03868	-0.07835	0.02298	-0.1009	0.06986	-0.06029
cg01101114	0.007869	0.1234	0.2991	-0.0306	0.009758	0.1199
cg01109454	0.8675	0.007718	0.03254	-0.1112	0.7696	0.01324
cg01110616	0.02269	-0.07548	0.08814	-0.03547	0.006846	-0.106
cg01117623	0.01723	0.1078	0.04231	-0.08426	0.07886	0.05863
cg01154508	0.02207	0.1121	0.349	0.04379	0.01653	0.108
cg01162358	0.01073	-0.1228	0.06407	-0.06054	0.008906	-0.1197
cg01163237	0.1333	0.07355	0.7428	0.01822	0.02888	0.1027
cg01169151	0.01587	-0.1177	0.07317	-0.08821	0.03298	-0.0885
cg01169259	0.0265	-0.1043	0.2815	-0.04539	0.01785	-0.119
cg01259782	0.2922	-0.03838	0.9565	0.002369	0.03736	-0.1104
cg01277410	0.2366	-0.02563	0.0256	-0.1126	0.02479	-0.06276
cg01282376	0.137	0.05519	0.8246	-0.01071	0.02252	0.104
cg01287850	0.03879	-0.13	0.7104	-0.02581	0.1023	-0.09432
cg01321862	0.07909	-0.04035	0.02559	-0.1017	0.05163	-0.05032
cg01344119	0.01268	-0.08693	0.02514	-0.0652	0.01056	-0.112
cg01351787	0.0574	-0.07623	0.1386	-0.06465	0.02519	-0.102
cg01368476	0.12	-0.04916	0.07475	-0.05592	0.01457	-0.1195
cg01394795	0.336	-0.03183	0.04858	-0.1047	0.5506	-0.01994
cg01396779	0.3863	-0.01909	0.01196	-0.1533	0.3717	-0.02728
cg01415122	0.04248	-0.1108	0.774	-0.01875	0.2798	-0.04508
cg01438011	0.04818	0.1125	0.8307	-0.01657	0.2018	0.05357

cg01440371	0.2348	0.06191	0.7261	-0.02541	0.02541	0.1206
cg01466163	0.03037	-0.1115	0.2972	-0.04922	0.1234	-0.07654
cg01474179	0.09095	-0.06488	0.01961	-0.1224	0.01075	-0.1505
cg01480256	0.7566	-0.01246	0.01954	-0.1538	0.5128	-0.02326
cg01491358	0.03577	0.1377	0.5501	0.03136	0.02934	0.1379
cg01498507	0.05785	0.08588	0.1921	0.05223	0.02933	0.1065
cg01512405	0.05732	0.08048	0.5561	0.02922	0.03759	0.1034
cg01517050	0.02844	-0.05056	0.006942	-0.1336	0.03791	-0.05768
cg01536941	0.04237	-0.1048	0.03041	-0.1103	0.06041	-0.08102
cg01553748	0.007999	-0.1069	0.05256	-0.06672	0.0163	-0.06623
cg01559222	0.966	-0.001977	0.04576	-0.1205	0.9362	-0.004176
cg01564380	0.04629	-0.1074	0.3555	-0.05742	0.09314	-0.08628
cg01566342	0.1243	-0.08186	0.03505	-0.1089	0.2289	-0.05765
cg01574741	0.05909	0.08378	0.4099	-0.02514	0.01784	0.1018
cg01575752	0.2845	-0.03258	0.9224	0.006259	0.03256	-0.1457
cg01591193	0.7101	-0.01587	0.0223	-0.1294	0.5628	-0.02904
cg01607771	0.04798	-0.1068	0.421	-0.03342	0.1543	-0.06603
cg01614101	0.01904	-0.0968	0.01569	-0.1391	0.102	-0.0908
cg01634612	0.0685	-0.1034	0.03558	-0.18	0.1423	-0.09265
cg01635267	0.1752	-0.04137	0.04069	-0.1011	0.6399	-0.01852
cg01659494	0.0433	-0.1195	0.9859	-0.001246	0.09126	-0.08603
cg01667018	0.4191	0.03092	0.02778	-0.1082	0.8003	0.01003
cg01741032	0.04855	-0.1022	0.2741	-0.0499	0.09866	-0.08338
cg01749549	0.1493	-0.08479	0.05468	-0.1124	0.02224	-0.149
cg01798774	0.6223	-0.01712	0.01858	-0.1185	0.3321	-0.03776
cg01806142	0.03574	-0.1946	0.1194	-0.1139	0.04579	-0.1489
cg01815536	0.3923	-0.02852	0.03314	-0.1069	0.2994	-0.03596
cg01820376	0.6813	-0.01842	0.03818	-0.1059	0.9427	-0.003524
cg01840401	0.01524	0.1068	0.2013	0.05006	0.007631	0.128
cg01843127	0.04423	-0.1322	0.04549	-0.1098	0.01703	-0.1303
cg01855601	0.2092	-0.05112	0.0238	-0.135	0.1047	-0.06553
cg01868835	0.03438	0.126	0.885	-0.01129	0.07724	0.09635
cg01873305	0.049	0.1619	0.9082	-0.0137	0.04104	0.1528
cg01876970	0.03145	0.1071	0.1788	0.04205	0.04138	0.0974
cg01877318	0.03116	-0.1753	0.4537	-0.06087	0.03824	-0.1272
cg01914007	0.02967	0.1242	0.9341	-0.006223	0.4313	0.04533
cg01914749	0.02099	0.1045	0.5358	0.02492	0.02186	0.09902
cg01922997	0.4334	0.03155	0.04887	-0.1186	0.6657	-0.02363
cg01958456	0.007025	0.1053	0.07158	0.05287	0.0161	0.1018
cg01963147	0.142	-0.04103	0.975	-0.001442	0.02409	-0.1033
cg02002217	0.9446	0.002965	0.8143	-0.01795	0.01339	0.1144
cg02006142	0.009966	0.1081	0.7597	-0.01372	0.7219	0.009658
cg02042481	0.01086	-0.1063	0.6741	0.01494	0.01971	-0.07808
cg02042710	0.04565	0.08296	0.589	0.02551	0.01472	0.101
cg02059296	0.05537	-0.07212	0.03367	-0.09205	0.02053	-0.1215
cg02068166	0.008231	0.1025	0.9164	-0.003079	0.04094	0.0746
cg02070906	0.03306	-0.08929	0.4204	-0.03522	0.02689	-0.1041
cg02077524	0.02515	-0.1031	0.5005	-0.02894	0.03644	-0.08895
cg02092708	0.01564	-0.05715	0.01727	-0.1004	0.04585	-0.04495
cg02124498	0.01682	-0.09855	0.2481	-0.03962	0.008092	-0.1107

cg02138992	0.8469	0.0104	0.03294	-0.1453	0.497	-0.03342
cg02180006	0.04807	0.1168	0.9993	6.34E-05	0.1884	0.05796
cg02198771	0.1398	-0.04825	0.01954	-0.1033	0.1831	-0.03608
cg02201720	0.05878	0.1125	0.2077	0.08787	0.04777	0.1352
cg02218444	0.02702	-0.1521	0.6486	-0.027	0.7025	-0.02269
cg02223684	0.433	-0.01338	0.00406	-0.131	0.0117	-0.06064
cg02242794	0.2115	-0.04408	0.01515	-0.1641	0.05645	-0.07615
cg02246790	0.2434	0.02714	0.01746	-0.1168	0.2692	0.02781
cg02297695	0.0922	-0.05673	0.03011	-0.115	0.04809	-0.06453
cg02299465	0.7712	-0.008698	0.01625	-0.1166	0.9405	-0.002917
cg02307266	0.02615	-0.1238	0.6068	-0.0277	0.09267	-0.08421
cg02317331	0.09491	-0.07988	0.9781	0.001731	0.03302	-0.103
cg02329430	0.7268	0.01147	0.04957	-0.1029	0.7903	-0.01072
cg02373104	0.02016	0.2061	0.9119	0.009701	0.1546	0.0873
cg02376178	0.02089	0.1223	0.06234	0.09573	0.0207	0.133
cg02389180	0.05747	-0.1222	0.7841	-0.02096	0.04578	-0.1336
cg02391239	0.1119	0.05436	0.83	0.00936	0.01231	0.1067
cg02397545	0.01173	0.1085	0.9949	0.0002264	0.05644	0.07785
cg02404407	0.03952	-0.09681	0.4289	-0.0348	0.007263	-0.131
cg02407808	0.1939	-0.05261	0.03136	-0.1211	0.3029	-0.0424
cg02444226	0.0427	-0.06953	0.06374	-0.07124	0.008092	-0.1047
cg02457108	0.2484	-0.04001	0.04667	-0.1028	0.8313	-0.00687
cg02458384	0.04721	0.1146	0.03033	0.1452	0.1255	0.06546
cg02481451	0.03278	-0.1133	0.8595	-0.009965	0.05705	-0.1023
cg02487233	0.08085	-0.105	0.9918	-0.000676	0.03122	-0.1204
cg02502679	0.04878	-0.06873	0.2856	-0.0383	0.01837	-0.1135
cg02506181	0.1189	-0.05775	0.02951	-0.1069	0.1823	-0.05081
cg02530533	0.09838	-0.06302	0.0708	-0.1003	0.03333	-0.102
cg02538079	0.8821	-0.004874	0.01358	-0.1081	0.8853	0.005089
cg02541555	0.1867	-0.05398	0.02766	-0.1232	0.1871	-0.05393
cg02551745	0.04507	-0.08165	0.0442	-0.1139	0.1894	-0.05341
cg02560595	0.04247	-0.1191	0.9483	0.003403	0.1443	-0.05486
cg02592739	0.02292	0.1306	0.6732	0.02771	0.02043	0.1331
cg02596384	0.04149	-0.1013	0.5861	-0.02975	0.1839	-0.06179
cg02611605	0.0405	-0.1078	0.1629	-0.1263	0.2132	-0.09769
cg02652579	0.7797	-0.01454	0.04612	-0.1174	0.503	-0.02636
cg02662046	0.03771	0.1042	0.1163	0.09492	0.123	0.07715
cg02676476	0.1328	-0.06312	0.02142	-0.1209	0.04846	-0.08926
cg02678306	0.5524	-0.01372	0.02894	-0.101	0.02022	-0.05887
cg02691783	0.01317	-0.1129	0.1803	-0.05391	0.009312	-0.09783
cg02699090	0.08856	-0.08773	0.04845	-0.1256	0.3618	-0.05913
cg02714176	0.01703	-0.1012	0.3424	-0.04466	0.1513	-0.06583
cg02737402	0.02482	0.1149	0.3906	-0.0646	0.1219	0.07066
cg02744670	0.01173	-0.1137	0.2246	-0.04969	0.0166	-0.1049
cg02756989	0.0996	-0.05588	0.3087	-0.04499	0.02235	-0.1014
cg02762593	0.03065	0.1069	0.8483	-0.008941	0.04003	0.08045
cg02763803	0.03864	-0.1008	0.2772	-0.05098	0.07816	-0.0682
cg02769615	0.3813	0.03179	0.03422	-0.1085	0.8032	0.009206
cg02771649	0.02748	-0.3874	0.4538	-0.06204	0.08984	0.145
cg02775883	0.1075	-0.08443	0.7677	-0.01653	0.0362	-0.1147

cg02779465	0.04543	-0.1816	0.9694	-0.005074	0.314	-0.08063
cg02796638	0.03962	0.1335	0.7847	-0.01958	0.03217	0.1454
cg02801914	0.9538	-0.002902	0.01848	-0.1474	0.8551	-0.009969
cg02830467	0.03335	0.1603	0.4822	0.05887	0.04721	0.1298
cg02839206	0.2349	-0.03014	0.01158	-0.1801	0.2853	-0.04045
cg02843573	0.01527	0.1093	0.4217	0.0324	0.03234	0.0813
cg02876747	0.08809	-0.06117	0.1419	-0.08164	0.02266	-0.1033
cg02886838	0.262	-0.03019	0.7654	0.01087	0.009543	-0.1055
cg02891633	0.05586	0.1148	0.6563	0.02535	0.01601	0.1226
cg02892885	0.04822	-0.176	0.9016	0.01187	0.129	-0.1207
cg02894083	0.1269	0.08267	0.7627	0.02008	0.03407	0.1108
cg02894224	0.1349	-0.03987	0.3299	-0.03202	0.01678	-0.1051
cg02900800	0.01284	0.1372	0.5085	0.0293	0.02742	0.08673
cg02902672	0.02418	0.11	0.4087	0.03693	0.1189	0.05729
cg02911689	0.0266	-0.08423	0.1161	-0.0569	0.01715	-0.1041
cg02912281	0.04844	-0.1042	0.4665	-0.04081	0.1188	-0.0867
cg02925848	0.03227	-0.1292	0.3554	-0.06091	0.06507	-0.1273
cg02947835	0.0238	-0.1149	0.2771	-0.04535	0.1077	-0.06554
cg02979978	0.1084	-0.04169	0.01122	-0.126	0.06686	-0.06425
cg02988795	0.06583	-0.05686	0.04394	-0.06656	0.0138	-0.1022
cg02997881	0.0493	-0.05008	0.01122	-0.09394	0.01095	-0.1024
cg03011445	0.04315	0.1024	0.2113	0.05852	0.3418	0.03348
cg03023681	0.03873	0.09023	0.6937	-0.01848	0.0196	0.1048
cg03062717	0.03526	-0.1093	0.5521	-0.03135	0.04078	-0.1084
cg03090276	0.0733	-0.06689	0.2048	-0.05047	0.0132	-0.1048
cg03120716	0.2525	0.05	0.9332	0.006516	0.04501	0.1077
cg03125444	0.03356	-0.09266	0.9367	0.004039	0.0186	-0.1043
cg03128522	0.9158	-0.006357	0.03182	-0.1432	0.4966	0.03384
cg03131219	0.9039	-0.004633	0.04496	-0.1123	0.4705	0.02509
cg03154717	0.02934	-0.0699	0.6717	-0.012	0.01058	-0.1075
cg03158874	0.3787	-0.02216	0.02302	-0.1111	0.2706	-0.03668
cg03167717	0.007869	-0.1359	0.1427	-0.05484	0.01005	-0.1165
cg03188522	0.179	0.0535	0.9727	0.002515	0.02305	0.1126
cg03189172	0.03936	-0.1175	0.5037	-0.04273	0.2843	-0.05434
cg03197835	0.6176	-0.01903	0.4835	-0.0341	0.01641	-0.111
cg03214420	0.02252	-0.1358	0.2712	-0.05284	0.01779	-0.1276
cg03227076	0.6072	-0.02202	0.5481	-0.03035	0.04679	-0.1036
cg03240624	0.06179	-0.07825	0.9414	-0.004025	0.03477	-0.111
cg03289224	0.1293	-0.09835	0.2665	-0.07675	0.03252	-0.1146
cg03309025	0.5698	-0.01878	0.8443	0.01001	0.04987	-0.114
cg03319082	0.03903	0.1232	0.5935	-0.02565	0.1658	0.06476
cg03319184	0.2729	-0.03732	0.04581	-0.1125	0.4933	-0.02836
cg03320208	0.05332	-0.03232	0.002293	-0.1259	0.8678	-0.003046
cg03329968	0.01212	-0.1029	0.8965	0.00408	0.01505	-0.0952
cg03331474	0.03761	-0.101	0.7307	0.01343	0.2063	-0.04038
cg03337575	0.01193	-0.1163	0.3569	-0.03092	0.03862	-0.09881
cg03362798	0.04867	0.1477	0.306	0.08209	0.02531	0.1767
cg03381408	0.00999	0.1685	0.666	-0.03326	0.2726	0.05467
cg03394309	0.0444	-0.1437	0.4214	-0.05403	0.2435	-0.05854
cg03394424	0.01317	0.1019	0.274	0.04397	0.0563	0.07085

cg03400587	0.04078	-0.1072	0.4782	-0.04607	0.1068	-0.08958
cg03413251	0.2921	-0.03137	0.9924	-0.0004719	0.01612	-0.102
cg03415649	0.4645	-0.02332	0.007439	-0.1035	0.2242	-0.02345
cg03417915	0.0125	-0.104	0.1131	-0.05096	0.01527	-0.09041
cg03418876	0.142	0.05488	0.02514	0.1283	0.1175	0.06898
cg03424550	0.07304	0.08086	0.03853	0.1184	0.06	0.08729
cg03426198	0.01698	-0.1291	0.1831	-0.04837	0.0254	-0.09209
cg03429643	0.02892	-0.06837	0.3949	-0.02296	0.00692	-0.1084
cg03448362	0.04394	0.1272	0.2772	-0.06913	0.7685	-0.02152
cg03455284	0.04903	-0.1368	0.08938	-0.1464	0.6769	-0.03248
cg03462525	0.007563	0.1271	0.904	0.003925	0.06484	0.0534
cg03473408	0.1955	-0.05832	0.04599	-0.1147	0.482	-0.03861
cg03486265	0.0364	-0.1004	0.2961	-0.04596	0.06406	-0.08639
cg03493083	0.03099	-0.07567	0.01122	-0.1096	0.01554	-0.08711
cg03498886	0.03544	0.1065	0.7889	-0.01425	0.2129	0.06172
cg03508427	0.03567	-0.1264	0.4097	-0.05006	0.1736	-0.09404
cg03532875	0.03868	-0.121	0.6481	0.02968	0.04238	-0.08511
cg03540917	0.03226	0.12	0.5742	0.03108	0.1611	0.05175
cg03564530	0.01268	0.116	0.6418	0.01994	0.0141	0.07664
cg03565772	0.04272	-0.1007	0.06428	-0.08949	0.1837	-0.05687
cg03589850	0.02249	-0.1094	0.9797	-0.0009612	0.2787	-0.04726
cg03592903	0.09596	-0.04338	0.02139	-0.1148	0.1197	-0.04453
cg03610869	0.3298	-0.03959	0.04564	-0.1225	0.2735	-0.04602
cg03624079	0.005857	-0.1144	0.3341	-0.02405	0.9015	-0.003817
cg03628365	0.0503	0.07764	0.05784	0.07319	0.01383	0.11
cg03629497	0.04328	-0.09775	0.06079	-0.1002	0.03359	-0.1145
cg03634650	0.0176	-0.1046	0.9843	0.0007662	0.02339	-0.07756
cg03646188	0.06242	0.08449	0.9657	0.002855	0.03526	0.1304
cg03651769	0.01944	0.1153	0.3165	0.04489	0.08605	0.07762
cg03674826	0.01941	0.08036	0.7835	-0.01104	0.01005	0.1009
cg03677069	0.0289	-0.3306	0.7866	-0.04165	0.1359	-0.2297
cg03680663	0.4274	-0.03228	0.04693	-0.1098	0.6539	-0.01919
cg03683612	0.97	0.002472	0.03232	-0.1088	0.8444	-0.009252
cg03684768	0.02814	-0.1055	0.4725	0.02999	0.09877	-0.07762
cg03691030	0.0282	-0.1165	0.07614	-0.1014	0.07844	-0.08364
cg03695083	0.4091	-0.02383	0.01351	-0.1155	0.04803	-0.06221
cg03766523	0.04534	-0.0552	0.304	-0.038	0.01269	-0.1243
cg03770722	0.03738	-0.1068	0.2725	-0.046	0.2528	-0.03412
cg03778809	0.04919	-0.1388	0.9775	-0.001921	0.06661	-0.09412
cg03794784	0.01718	-0.1429	0.6576	-0.01659	0.1794	-0.05519
cg03797593	0.6129	-0.02129	0.04232	-0.1235	0.2112	-0.04516
cg03800141	0.01482	0.1126	0.09417	0.05951	0.209	0.03463
cg03854025	0.02011	-0.07925	0.03898	-0.0944	0.02314	-0.1142
cg03880708	0.08145	-0.08663	0.7519	-0.0156	0.02422	-0.1102
cg03885197	0.2265	-0.03905	0.04195	-0.1003	0.1777	-0.04847
cg03903988	0.04909	0.1211	0.8088	-0.01495	0.06932	0.1067
cg03913374	0.9091	0.00516	0.03162	-0.1118	0.2834	-0.04965
cg03917822	0.04175	0.09394	0.1056	0.05813	0.01721	0.1148
cg03936251	0.3588	-0.03786	0.02728	-0.1169	0.8109	-0.01019
cg03941746	0.005874	-0.1714	0.802	-0.01161	0.01973	-0.1099

cg03944512	0.2182	-0.03937	0.1637	-0.05151	0.02011	-0.1134
cg03947317	0.05149	-0.07525	0.1163	-0.05445	0.008092	-0.1048
cg03967240	0.6952	-0.01851	0.0611	-0.07864	0.01217	-0.1046
cg03982350	0.5135	-0.01995	0.02362	-0.1056	0.1043	-0.06071
cg04012844	0.1207	-0.1076	0.8206	0.01644	0.03555	-0.1411
cg04078732	0.1374	-0.06404	0.03496	-0.109	0.3803	-0.0328
cg04080801	0.9801	0.0008037	0.01528	-0.1245	0.6374	0.01464
cg04104489	0.007999	-0.2803	0.05372	-0.1325	0.008836	-0.1839
cg04107410	0.0222	0.1124	0.8529	0.0101	0.01626	0.08617
cg04122493	0.3949	-0.03236	0.2543	-0.05092	0.02381	-0.1097
cg04146573	0.01364	-0.08064	0.9114	-0.004215	0.01016	-0.1017
cg04153536	0.1653	-0.06388	0.03767	-0.1102	0.8281	-0.009742
cg04208160	0.0343	0.105	0.9508	0.003666	0.07102	0.07267
cg04219941	0.1835	0.0354	0.0474	-0.1268	0.9292	0.00356
cg04246123	0.05976	0.0974	0.5363	-0.03681	0.02385	0.1254
cg04258358	0.007524	-0.09824	0.3823	-0.02088	0.02235	-0.1041
cg04259358	0.1711	-0.05769	0.7322	-0.01627	0.0163	-0.1028
cg04262961	0.973	-0.0009149	0.04692	-0.1022	0.5648	0.008793
cg04280449	0.03529	-0.07529	0.03023	-0.129	0.05892	-0.05676
cg04304779	0.1303	0.07186	0.9823	-0.001842	0.0424	0.1034
cg04307083	0.08275	0.1127	0.7652	0.0226	0.04444	0.1227
cg04330126	0.1358	-0.06204	0.6779	0.02306	0.0385	-0.1015
cg04332110	0.029	-0.108	0.9804	0.001489	0.09047	-0.07263
cg04341707	0.02017	-0.1226	0.7255	-0.02026	0.6587	-0.01821
cg04400679	0.2311	0.0483	0.7226	-0.01658	0.04379	0.1102
cg04409954	0.3363	-0.02709	0.01625	-0.1148	0.336	-0.0183
cg04415706	0.1017	0.08955	0.4441	0.03976	0.03179	0.1275
cg04420115	0.1127	-0.07729	0.04693	-0.1227	0.2677	-0.04384
cg04429324	0.8003	0.0101	0.03541	-0.1024	0.3434	0.03284
cg04436782	0.024	-0.1114	0.1849	-0.06667	0.1096	-0.06371
cg04470754	0.02881	-0.11	0.3721	-0.03602	0.0588	-0.07477
cg04479580	0.04023	-0.107	0.3562	-0.04999	0.1265	-0.0688
cg04488111	0.06004	0.09337	0.4411	-0.03149	0.02561	0.1316
cg04497094	0.04514	0.1322	0.8321	-0.0196	0.1315	0.0824
cg04502411	0.008881	-0.124	0.214	-0.04326	0.02434	-0.09147
cg04511104	0.1491	-0.06582	0.06355	-0.1013	0.03789	-0.1033
cg04519723	0.008107	-0.1245	0.3438	-0.03215	0.03346	-0.09116
cg04542080	0.02153	-0.1054	0.4789	-0.0286	0.05035	-0.07186
cg04542290	0.03799	-0.06065	0.1021	-0.0426	0.006465	-0.1022
cg04574723	0.04685	-0.1364	0.6142	0.03083	0.1663	-0.07674
cg04590790	0.65	0.01859	0.6217	0.02419	0.0285	0.1117
cg04598693	0.03672	0.1011	0.9822	-0.001498	0.06893	0.09489
cg04625976	0.03955	0.1056	0.804	-0.01134	0.08867	0.09272
cg04648412	0.02232	-0.09055	0.05013	-0.06784	0.01121	-0.1092
cg04653639	0.02539	-0.117	0.07668	-0.07874	0.02794	-0.1076
cg04657715	0.09829	0.09635	0.8224	0.01434	0.0436	0.1048
cg04674406	0.005874	0.07964	0.141	0.02852	0.002329	0.1058
cg04678950	0.0417	-0.09254	0.03615	-0.1005	0.1394	-0.06194
cg04717613	0.02574	0.1262	0.7959	-0.01373	0.01204	0.1048
cg04720283	0.03051	0.1301	0.976	0.001703	0.07668	0.08985

cg04759456	0.01517	0.1014	0.506	-0.03205	0.1742	0.05416
cg04760530	0.5144	-0.02884	0.04604	-0.1165	0.03325	-0.1098
cg04789818	0.06528	-0.1141	0.3822	-0.05325	0.04409	-0.1064
cg04804604	0.01471	-0.113	0.01858	-0.1017	0.007416	-0.1082
cg04808380	0.4797	-0.02161	0.02212	-0.1105	0.8621	-0.005952
cg04815645	0.1572	0.07164	0.8743	-0.01076	0.04141	0.1181
cg04827268	0.09805	-0.06294	0.02894	-0.1132	0.1468	-0.04489
cg04841837	0.07166	-0.06724	0.03446	-0.1066	0.02323	-0.1061
cg04880120	0.2408	-0.07605	0.7363	-0.01924	0.04613	-0.1147
cg04890706	0.007219	0.1389	0.1325	0.07526	0.00692	0.1536
cg04892765	0.01221	0.1266	0.826	0.007784	0.03318	0.07906
cg04917860	0.09051	0.07243	0.3643	0.04074	0.0397	0.1002
cg04980339	0.03694	0.09567	0.3178	0.05896	0.03127	0.1222
cg05005786	0.3678	-0.03296	0.04721	-0.1155	0.2321	-0.04292
cg05017130	0.1284	-0.06633	0.04053	-0.1197	0.09369	-0.06557
cg05019740	0.03838	-0.1277	0.5313	-0.03895	0.4779	-0.03488
cg05020409	0.0662	-0.1308	0.09036	-0.1399	0.02349	-0.1825
cg05045685	0.01897	0.1096	0.4013	0.02972	0.02074	0.09797
cg05063920	0.04551	0.1005	0.4791	0.03559	0.1846	0.05204
cg05065816	0.04049	-0.124	0.1183	-0.1003	0.06033	-0.1176
cg05096467	0.03472	0.1064	0.987	0.001142	0.06099	0.1107
cg05115460	0.01788	-0.1096	0.06275	-0.08948	0.08225	-0.06244
cg05116781	0.2707	-0.04376	0.04188	-0.1181	0.1115	-0.05756
cg05131620	0.04085	-0.1015	0.6469	-0.0236	0.03094	-0.1116
cg05136238	0.3047	0.03397	0.01505	-0.1131	0.9044	-0.004768
cg05137157	0.004198	-0.1035	0.1517	-0.05046	0.008319	-0.07558
cg05146534	0.5987	-0.01927	0.02809	-0.1311	0.02521	-0.1122
cg05174618	0.1465	-0.09009	0.0474	-0.1471	0.1023	-0.1206
cg05189232	0.04594	-0.08789	0.04053	-0.1132	0.01088	-0.131
cg05194509	0.09573	-0.04561	0.006942	-0.1121	0.0628	-0.06582
cg05199232	0.02732	0.1188	0.2925	-0.04306	0.4885	0.03219
cg05238074	0.03498	0.1068	0.3567	0.04104	0.03115	0.1177
cg05243629	0.1009	-0.09354	0.1388	-0.08959	0.02717	-0.1188
cg05316708	0.04378	0.1059	0.8571	0.009672	0.2089	0.05755
cg05381852	0.03105	-0.0931	0.2351	-0.05085	0.02278	-0.1006
cg05396624	0.099	0.07991	0.1601	-0.0976	0.0247	0.1297
cg05428706	0.1365	-0.0699	0.6091	-0.03069	0.03408	-0.1096
cg05429527	0.03589	0.09999	0.1434	-0.05089	0.007416	0.1103
cg05497451	0.3815	-0.05604	0.03759	-0.1548	0.4923	-0.04556
cg05503024	0.7085	-0.01648	0.04271	-0.1047	0.7015	-0.01829
cg05509190	0.05267	0.1026	0.2947	0.06452	0.02506	0.1199
cg05532541	0.02557	-0.1009	0.9425	0.003148	0.1624	-0.04817
cg05565863	0.01262	0.08245	0.9517	-0.002936	0.006801	0.1116
cg05575321	0.05766	-0.1181	0.2085	-0.0669	0.008544	-0.1578
cg05592345	0.1048	0.06825	0.3385	-0.04086	0.01927	0.1197
cg05600421	0.9768	-0.001683	0.935	-0.005322	0.02279	-0.1167
cg05603680	0.02213	-0.08681	0.06776	-0.08318	0.04941	-0.1018
cg05609536	0.0318	0.1112	0.3418	0.04062	0.06401	0.07508
cg05610083	0.04154	0.1015	0.403	-0.049	0.3981	0.03948
cg05635340	0.9099	0.003907	0.02587	-0.106	0.1598	-0.05032

cg05636015	0.04974	-0.1035	0.93	-0.005329	0.06237	-0.08414
cg05641446	0.03193	-0.1464	0.9373	-0.005246	0.08707	-0.09158
cg05644223	0.3705	-0.03405	0.04765	-0.1051	0.3588	-0.03081
cg05653385	0.01044	-0.08791	0.05906	-0.05205	0.008544	-0.1039
cg05660711	0.2349	-0.05924	0.1643	-0.06273	0.02489	-0.1287
cg05661717	0.02051	0.109	0.6359	-0.02376	0.1648	0.05513
cg05665937	0.03125	-0.1252	0.5834	0.02771	0.06477	-0.1016
cg05706661	0.03025	-0.1315	0.5389	-0.03532	0.03059	-0.1221
cg05712421	0.0391	-0.1004	0.3825	-0.03826	0.04236	-0.0842
cg05715544	0.02926	0.1123	0.5537	-0.02026	0.01209	0.09787
cg05731995	0.02197	0.1038	0.9156	0.004523	0.01703	0.09264
cg05741666	0.07596	0.08178	0.7613	-0.01736	0.02413	0.1164
cg05746918	0.03536	0.1007	0.9025	0.006301	0.1089	0.06346
cg05759166	0.04128	-0.1097	0.9438	-0.004632	0.1419	-0.0619
cg05764108	0.02616	-0.1199	0.7465	-0.01582	0.2012	-0.04976
cg05766539	0.02022	-0.1343	0.4574	-0.03564	0.1255	-0.06579
cg05773702	0.02813	0.1062	0.4594	0.02683	0.4834	0.02213
cg05806875	0.02317	-0.1001	0.9221	-0.003585	0.06111	-0.05547
cg05838101	0.01669	0.09433	0.9406	0.002977	0.008319	0.1315
cg05842490	0.8174	-0.007313	0.01566	-0.1059	0.065	0.05345
cg05857097	0.2156	-0.03395	0.02562	-0.1182	0.134	-0.07493
cg05859257	0.03311	0.09801	0.777	0.01326	0.02802	0.1074
cg05867410	0.03868	-0.06559	0.3795	-0.03084	0.04531	-0.1062
cg05869837	0.07822	0.06867	0.5647	-0.02456	0.01956	0.1155
cg05875486	0.03745	-0.1084	0.3981	0.03593	0.1321	-0.06149
cg05876665	0.04267	-0.102	0.9766	0.001609	0.3223	-0.04351
cg05898320	0.9141	0.004416	0.03343	-0.1017	0.438	-0.03244
cg05905568	0.02486	-0.105	0.7506	-0.01524	0.9596	0.003165
cg05962428	0.02907	0.1012	0.7386	0.01636	0.01385	0.09357
cg05975755	0.03278	-0.07726	0.06022	-0.07906	0.006777	-0.1025
cg06000275	0.03061	-0.1022	0.7583	0.01204	0.5164	0.03248
cg06003912	0.0128	-0.07269	0.01575	-0.07094	0.006846	-0.1044
cg06016475	0.4335	-0.02447	0.04581	-0.1221	0.5123	0.01752
cg06024187	0.3499	-0.0493	0.04814	-0.1454	0.1374	-0.08565
cg06043208	0.01005	-0.07591	0.01498	-0.1085	0.01634	-0.07122
cg06063452	0.04749	-0.11	0.9613	-0.002494	0.04993	-0.07979
cg06066072	0.02967	0.09953	0.4888	-0.02885	0.0199	0.1023
cg06074920	0.2919	-0.04495	0.7972	-0.01483	0.01739	-0.1073
cg06087428	0.03772	-0.1318	0.6999	-0.02909	0.1648	-0.08249
cg06111254	0.7379	-0.01221	0.02903	-0.1027	0.5964	0.0191
cg06115018	0.1769	-0.0396	0.03746	-0.1184	0.2304	-0.05444
cg06121193	0.2354	-0.05799	0.9848	0.00112	0.0346	-0.103
cg06133619	0.9973	-0.000197	0.03374	-0.1253	0.611	-0.02243
cg06140237	0.0163	0.1177	0.07604	0.05656	0.006846	0.1178
cg06173889	0.02484	0.09774	0.3719	-0.02659	0.01961	0.1034
cg06200449	0.2405	-0.05855	0.0872	-0.119	0.03406	-0.1419
cg06219801	0.254	0.04846	0.5551	-0.02811	0.04859	0.101
cg06255281	0.1579	0.08396	0.06989	-0.1175	0.03944	0.1274
cg06256735	0.09766	0.08524	0.8023	0.01904	0.04648	0.1089
cg06259377	0.01348	-0.1314	0.2485	-0.0482	0.09027	-0.0676

cg06285596	0.4712	-0.02243	0.01762	-0.1244	0.3397	-0.03027
cg06295294	0.09875	-0.0645	0.9492	0.002716	0.01824	-0.1006
cg06296759	0.6659	0.01268	0.02141	-0.1068	0.5943	-0.02406
cg06297160	0.01539	-0.1479	0.5831	-0.03262	0.2275	-0.06307
cg06301987	0.278	-0.04149	0.04347	-0.1092	0.1562	-0.06028
cg06305375	0.1059	-0.04715	0.01566	-0.1073	0.06392	-0.05599
cg06313881	0.1723	-0.04602	0.02389	-0.1034	0.2234	-0.04052
cg06324137	0.5184	0.03573	0.9839	0.00144	0.03877	0.1046
cg06325495	0.02847	-0.1168	0.7488	0.0144	0.3065	-0.04569
cg06345856	0.09908	-0.06397	0.9296	-0.004143	0.01479	-0.1353
cg06360274	0.008107	-0.1756	0.7732	-0.01581	0.02892	-0.1126
cg06361793	0.02752	0.1059	0.1298	0.06462	0.0247	0.08499
cg06375758	0.1074	-0.04154	0.01589	-0.1009	0.01966	-0.0578
cg06379340	0.3957	-0.03527	0.03343	-0.1204	0.1919	-0.06499
cg06391889	0.04954	0.1273	0.8384	-0.01286	0.07093	0.09556
cg06401677	0.2957	-0.03295	0.02927	-0.142	0.07912	-0.04293
cg06425081	0.9432	0.002212	0.01566	-0.1012	0.1281	-0.05391
cg06426828	0.06393	-0.09632	0.1115	-0.08464	0.04493	-0.1109
cg06485214	0.03427	-0.08408	0.8735	-0.006951	0.02413	-0.1013
cg06505783	0.07642	0.1243	0.5535	0.04758	0.02714	0.1693
cg06514994	0.1837	-0.05301	0.02389	-0.127	0.04809	-0.08501
cg06528823	0.1243	-0.03595	0.01579	-0.12	0.05224	-0.07506
cg06533798	0.02498	0.135	0.08152	0.07325	0.07884	0.07508
cg06548300	0.3793	0.0258	0.02024	-0.1032	0.3286	-0.02992
cg06562103	0.2261	-0.07298	0.5281	-0.03767	0.04733	-0.1219
cg06568490	0.02755	-0.1058	0.516	0.02965	0.1493	-0.05752
cg06611109	0.01471	-0.1047	0.02024	-0.06443	0.03355	-0.06926
cg06646197	0.01317	0.1095	0.3183	-0.0406	0.02395	0.07874
cg06648955	0.01674	0.09361	0.1694	0.04448	0.0146	0.1282
cg06667609	0.8701	0.01141	0.04515	-0.1647	0.8243	0.01698
cg06675348	0.03066	-0.1001	0.8345	0.011	0.167	-0.05837
cg06702499	0.6447	-0.0193	0.8571	-0.0125	0.03869	-0.1409
cg06712913	0.05706	-0.04872	0.01625	-0.1033	0.1395	-0.03032
cg06734940	0.04999	-0.106	0.9198	-0.004658	0.07012	-0.08426
cg06771890	0.0178	0.1582	0.5539	0.03097	0.03005	0.1111
cg06786335	0.1656	-0.06998	0.04074	-0.1438	0.04061	-0.1056
cg06789134	0.6012	-0.0157	0.04131	-0.1102	0.1546	-0.04758
cg06801615	0.03559	-0.133	0.9255	0.005925	0.1279	-0.07721
cg06840429	0.05332	-0.04525	0.01196	-0.1014	0.002329	-0.08486
cg06854197	0.02924	-0.1037	0.2821	-0.05126	0.04822	-0.09036
cg06878960	0.02824	-0.1218	0.8334	-0.008478	0.0269	-0.1144
cg06898293	0.5696	-0.02035	0.01994	-0.1234	0.3693	-0.03408
cg06925060	0.08422	-0.08182	0.1097	-0.07657	0.01903	-0.1238
cg06926703	0.9746	-0.001004	0.01122	-0.1292	0.6858	-0.0153
cg06938767	0.03183	-0.1335	0.2546	-0.06334	0.01512	-0.1707
cg06949395	0.01991	-0.138	0.577	-0.01977	0.1073	-0.06518
cg06973095	0.04424	0.08259	0.9821	0.001454	0.03417	0.1171
cg06979924	0.09488	0.06938	0.432	0.03127	0.008745	0.1059
cg06986743	0.1745	0.04797	0.9507	-0.003636	0.02391	0.1166
cg07019835	0.0169	-0.1044	0.4865	-0.02818	0.3161	-0.03546

cg07033513	0.1437	-0.03314	0.0136	-0.08183	0.006578	-0.1017
cg07053481	0.01365	0.1231	0.4392	0.0353	0.02324	0.1028
cg07053668	0.03459	0.1012	0.2283	-0.05733	0.04468	0.1047
cg07055718	0.0004002	-0.1033	0.02605	-0.08669	0.005348	-0.09463
cg07077303	0.0205	0.1139	0.7418	0.01244	0.1137	0.05576
cg07083854	0.5277	-0.01398	0.01906	-0.1032	0.9571	-0.001821
cg07098102	0.1094	0.09575	0.7303	-0.03083	0.04356	0.1288
cg07108021	0.2549	0.06163	0.04692	-0.1235	0.88	0.009486
cg07124457	0.3389	-0.04439	0.05027	-0.09887	0.03684	-0.1277
cg07130406	0.8765	-0.006348	0.03766	-0.1063	0.9304	0.004026
cg07135540	0.01893	0.1423	0.3338	0.03112	0.07992	0.07855
cg07162914	0.03666	-0.1185	0.6965	-0.02127	0.01816	-0.144
cg07204602	0.02131	0.1345	0.2173	0.05932	0.01829	0.1136
cg07213119	0.4218	-0.02802	0.5217	-0.02515	0.01902	-0.1137
cg07232711	0.01062	-0.1471	0.09609	-0.05262	0.04857	-0.08849
cg07236562	0.4279	-0.02181	0.02042	-0.131	0.01759	-0.07496
cg07254048	0.04829	0.05173	0.629	-0.02066	0.01742	0.1102
cg07267674	0.006311	-0.1058	0.09198	-0.05892	0.006041	-0.1053
cg07271580	0.03305	-0.1011	0.5595	-0.02461	0.04853	-0.07925
cg07271653	0.999	4.02E-05	0.03005	-0.1152	0.9272	-0.003596
cg07287156	0.03687	0.05985	0.4662	-0.02585	0.008836	0.1041
cg07318939	0.04305	0.1083	0.5849	0.02995	0.02337	0.115
cg07342117	0.03696	-0.09422	0.02251	-0.1297	0.01029	-0.1391
cg07346177	0.01747	-0.1106	0.1106	-0.06709	0.01314	-0.1194
cg07356654	0.6992	-0.01816	0.03104	-0.1343	0.1698	-0.05156
cg07357324	0.04007	0.09006	0.202	-0.04075	0.008621	0.1078
cg07359376	0.1883	-0.03774	0.02895	-0.102	0.3354	-0.02826
cg07376732	0.06368	0.08652	0.8257	0.01265	0.03963	0.1136
cg07380881	0.2073	-0.04607	0.3336	-0.05122	0.02256	-0.112
cg07386121	0.2831	-0.03831	0.04442	-0.1002	0.4886	-0.0231
cg07396477	0.02625	-0.1242	0.4111	-0.04331	0.07059	-0.1114
cg07397867	0.0357	-0.04983	0.01625	-0.1034	0.3402	-0.02323
cg07416098	0.4438	-0.03455	0.03672	-0.136	0.8605	-0.009339
cg07519598	0.04801	-0.1009	0.2335	-0.05169	0.01308	-0.1523
cg07528445	0.04254	-0.1362	0.9429	-0.005356	0.07108	-0.1001
cg07529660	0.0283	-0.06822	0.02499	-0.07171	0.00656	-0.1016
cg07557612	0.7817	-0.008975	0.01894	-0.1097	0.8069	-0.008006
cg07558672	0.02509	0.1049	0.9477	0.00288	0.03954	0.0791
cg07562175	0.02226	0.07675	0.3381	0.0376	0.01721	0.1022
cg07572596	0.7696	-0.01215	0.04779	-0.1048	0.72	-0.01806
cg07573238	0.3635	0.02676	0.03775	-0.1377	0.5302	0.02403
cg07582245	0.02965	0.1017	0.9232	-0.006414	0.05622	0.07741
cg07583756	0.03394	0.1167	0.8977	0.009133	0.2715	0.05352
cg07617237	0.2328	-0.04567	0.04692	-0.1047	0.2211	-0.0394
cg07620831	0.07775	-0.07622	0.03849	-0.1099	0.2927	-0.04147
cg07680368	0.04929	-0.05848	0.02711	-0.1013	0.6765	-0.0128
cg07696542	0.1149	0.06791	0.839	0.01269	0.02442	0.1051
cg07705004	0.9223	0.006001	0.03277	-0.1315	0.9755	-0.002053
cg07715041	0.03035	0.09127	0.3887	0.0306	0.02205	0.1027
cg07717559	0.01674	0.1106	0.1171	0.08128	0.008895	0.1175

cg07730007	0.06685	0.1188	0.8364	0.0173	0.04309	0.1784
cg07735461	0.1315	-0.05717	0.02706	-0.1102	0.779	0.008026
cg07743467	0.06625	0.06682	0.02509	0.1161	0.335	0.03289
cg07748847	0.04753	-0.07416	0.1394	-0.05426	0.01398	-0.1123
cg07761273	0.02869	0.1078	0.6254	0.03254	0.04098	0.1165
cg07781847	0.01392	-0.0745	0.05352	-0.05898	0.006846	-0.1252
cg07823855	0.7456	-0.0126	0.09833	-0.05939	0.04718	-0.1076
cg07830794	0.0669	-0.09878	0.8886	-0.007318	0.01398	-0.102
cg07831164	0.1092	0.07802	0.6917	-0.01859	0.03829	0.1004
cg07842837	0.01694	-0.1573	0.421	-0.03257	0.07476	-0.08141
cg07861170	0.4385	-0.02284	0.01906	-0.1161	0.2977	-0.02781
cg07874127	0.1886	-0.05421	0.04721	-0.1175	0.7728	-0.01121
cg07886914	0.01635	-0.1059	0.1943	-0.05063	0.03554	-0.08412
cg07914250	0.4552	-0.02556	0.02957	-0.1023	0.4365	-0.0269
cg07925411	0.008254	0.1005	0.201	0.03762	0.01468	0.08895
cg07948472	0.4681	-0.02549	0.8665	0.008414	0.02726	-0.1011
cg07961310	0.1171	-0.045	0.01579	-0.1057	0.3273	-0.02519
cg07961467	0.005874	0.1113	0.6962	0.009232	0.002259	0.1048
cg08008402	0.1753	-0.03643	0.03454	-0.1154	0.758	-0.01096
cg08009458	0.9331	-0.005889	0.3489	0.07145	0.04994	0.1171
cg08017210	0.09104	0.127	0.3521	-0.07576	0.04793	0.1536
cg08026055	0.9285	-0.004821	0.04231	-0.1324	0.3204	-0.05444
cg08045042	0.07563	0.07279	0.3087	0.0351	0.01487	0.104
cg08095896	0.6206	-0.03301	0.03615	-0.1646	0.8677	-0.01245
cg08097446	0.01429	-0.06988	0.02409	-0.1004	0.06872	-0.05198
cg08118042	0.01126	0.1025	0.8722	-0.005274	0.05027	0.0619
cg08120184	0.3253	-0.04588	0.04442	-0.1242	0.218	-0.07264
cg08145700	0.02448	0.1173	0.9677	0.002695	0.07696	0.07755
cg08189964	0.5426	-0.01672	0.04206	-0.1093	0.2334	-0.04135
cg08212576	0.02368	0.1348	0.8586	0.01088	0.02433	0.1365
cg08230150	0.01044	-0.1509	0.1862	-0.05598	0.07803	-0.07094
cg08242132	0.05365	-0.06392	0.1665	-0.05767	0.01427	-0.1058
cg08251761	0.09252	-0.09009	0.9556	-0.003785	0.03793	-0.1058
cg08266275	0.7687	-0.01764	0.04667	-0.1428	0.3596	-0.05339
cg08291024	0.1757	-0.06575	0.9652	0.002861	0.04335	-0.1089
cg08319139	0.2689	-0.03737	0.04461	-0.1011	0.5802	-0.02231
cg08336417	0.02244	-0.1344	0.1729	-0.06796	0.04207	-0.09794
cg08339825	0.05693	-0.108	0.6615	-0.02232	0.03825	-0.1031
cg08373822	0.2092	0.05027	0.7747	0.01625	0.03389	0.1098
cg08384163	0.07255	-0.02988	0.1916	-0.03225	0.005224	-0.122
cg08389123	0.7848	0.008393	0.01672	-0.1294	0.8468	-0.006957
cg08394012	0.06487	0.07472	0.1759	0.058	0.02742	0.1053
cg08411224	0.0328	0.1026	0.5478	0.02625	0.01354	0.1165
cg08474897	0.02926	0.09277	0.9824	-0.001389	0.01287	0.1161
cg08499756	0.05056	-0.123	0.3126	-0.06886	0.04252	-0.1376
cg08515910	0.02305	-0.09492	0.2021	-0.04156	0.0163	-0.1116
cg08531698	0.05068	-0.07523	0.9566	0.002333	0.01989	-0.1001
cg08541788	0.01795	-0.08573	0.1374	-0.04882	0.006578	-0.1142
cg08562209	0.03633	-0.06992	0.03188	-0.1028	0.007818	-0.1227
cg08570574	0.07136	-0.07932	0.9725	0.002025	0.01857	-0.1094

cg08575894	0.008338	0.112	0.737	0.009348	0.01797	0.08653
cg08607175	0.08738	-0.06057	0.01429	-0.1377	0.2167	-0.0468
cg08628561	0.01284	-0.1179	0.5942	-0.02049	0.09154	-0.05318
cg08649440	0.5432	0.01829	0.02961	-0.106	0.2933	0.03073
cg08658810	0.8844	-0.009175	0.9098	0.0105	0.01548	0.1558
cg08659539	0.1272	-0.06729	0.9608	-0.002578	0.0224	-0.109
cg08683548	0.3065	-0.0418	0.3353	-0.04984	0.03459	-0.1047
cg08688498	0.0774	0.04551	0.01358	-0.109	0.07892	-0.05536
cg08691372	0.00915	0.1086	0.5223	-0.03186	0.7372	0.01184
cg08718713	0.01459	0.1036	0.5815	0.01597	0.01072	0.09187
cg08718844	0.4085	-0.04203	0.02419	-0.1182	0.5725	-0.02286
cg08751988	0.01929	-0.1107	0.6949	-0.01981	0.1193	-0.08322
cg08753403	0.008881	-0.05909	0.01625	-0.08218	0.004355	-0.125
cg08761909	0.807	0.01059	0.03925	-0.1092	0.6862	0.01753
cg08787039	0.1242	-0.05246	0.02539	-0.1125	0.371	-0.02988
cg08819934	0.03102	-0.1027	0.4843	0.03898	0.1661	-0.06745
cg08842154	0.1588	0.02917	0.02221	-0.1036	0.9779	-0.0009663
cg08857745	0.4202	-0.02693	0.04534	-0.1136	0.1238	-0.06983
cg08858393	0.03312	-0.1497	0.9795	-0.002066	0.03453	-0.1373
cg08874974	0.03661	0.1109	0.4094	-0.045	0.1527	0.05429
cg08881024	0.01909	0.1096	0.4471	0.03102	0.0273	0.09613
cg08881610	0.2155	-0.04689	0.02603	-0.134	0.111	-0.05491
cg08895562	0.1211	-0.04725	0.02054	-0.1104	0.08656	-0.04701
cg08908482	0.02115	-0.1327	0.3291	-0.04894	0.03665	-0.1277
cg08929862	0.7356	0.008542	0.0177	-0.1013	0.1924	-0.03617
cg08939373	0.1509	-0.04125	0.006942	-0.124	0.1174	-0.04017
cg08975998	0.04971	0.102	0.6609	0.0208	0.1122	0.07447
cg08980265	0.03361	-0.1055	0.5658	-0.0254	0.03291	-0.1109
cg08991104	0.1741	-0.04315	0.02605	-0.1132	0.5105	-0.02219
cg09015797	0.5534	0.02288	0.04273	0.102	0.4972	0.02689
cg09021390	0.1469	0.05893	0.9262	0.004699	0.02102	0.1015
cg09047188	0.7175	-0.01125	0.0263	-0.1096	0.5644	-0.01957
cg09048907	0.8868	0.005144	0.03229	-0.1216	0.222	-0.04935
cg09079417	0.03646	-0.08841	0.04608	-0.1249	0.1205	-0.06655
cg09089239	0.6784	0.01915	0.03013	-0.1215	0.5137	-0.02424
cg09097187	0.3075	-0.03112	0.9982	0.0001156	0.01895	-0.1053
cg09112723	0.02865	-0.1342	0.263	-0.06069	0.03781	-0.09561
cg09125508	0.04026	-0.08123	0.1614	-0.05236	0.005098	-0.162
cg09126880	0.4975	-0.01618	0.03538	-0.1046	0.1032	-0.04403
cg09142647	0.1664	-0.08201	0.03086	-0.1558	0.3542	-0.05328
cg09146892	0.02002	-0.155	0.02605	-0.1331	0.06437	-0.1121
cg09181453	0.1316	-0.0784	0.0448	-0.1448	0.55	-0.03688
cg09196248	0.03735	0.09975	0.4747	0.03559	0.01641	0.1466
cg09210935	0.0831	0.07245	0.6165	-0.0296	0.01866	0.1114
cg09230667	0.0413	-0.118	0.8569	-0.009896	0.05117	-0.08438
cg09236600	0.01591	0.1062	0.51	0.02162	0.01918	0.099
cg09249173	0.0409	-0.1367	0.4713	-0.04377	0.05636	-0.1325
cg09290103	0.6803	-0.01615	0.03872	-0.09975	0.02089	-0.103
cg09291739	0.1881	-0.04579	0.02351	-0.1176	0.303	-0.04122
cg09304355	0.05603	-0.06069	0.0241	-0.05673	0.002329	-0.1014

cg09307259	0.02384	-0.07146	0.06668	-0.04871	0.007433	-0.1085
cg09307266	0.02423	-0.1763	0.1312	-0.103	0.01025	-0.2087
cg09323390	0.2135	-0.05323	0.2148	-0.0768	0.03211	-0.1061
cg09330274	0.2311	0.03974	0.9376	-0.004302	0.007919	0.1358
cg09337948	0.0332	-0.1344	0.6133	0.02459	0.2332	-0.04893
cg09339360	0.2795	-0.02817	0.01358	-0.121	0.1284	-0.03899
cg09375768	0.1672	-0.04267	0.01734	-0.1168	0.1679	-0.03304
cg09406856	0.01691	-0.1206	0.386	-0.03558	0.02779	-0.09322
cg09417864	0.007999	0.1733	0.07461	-0.07919	0.01256	0.1435
cg09425753	0.03633	0.3174	0.4215	-0.09774	0.9453	0.01671
cg09439027	0.6117	0.01473	0.01746	-0.112	0.7277	-0.01039
cg09460999	0.03666	-0.1204	0.1378	-0.09507	0.04646	-0.1367
cg09496544	0.06431	-0.05641	0.01223	-0.1332	0.01973	-0.083
cg09523886	0.04365	-0.06177	0.02024	-0.1139	0.08759	-0.05546
cg09524650	0.0242	-0.1083	0.9188	-0.004159	0.1011	-0.07267
cg09539425	0.1427	0.03079	0.02382	-0.1105	0.08518	0.03745
cg09540471	0.03812	0.1084	0.6068	0.0248	0.01979	0.1149
cg09558638	0.0913	-0.0979	0.2241	-0.0909	0.02206	-0.1829
cg09567048	0.9431	0.002661	0.03924	-0.1082	0.2479	-0.04286
cg09569760	0.6928	0.02296	0.04301	-0.162	0.5947	-0.03102
cg09575960	0.03039	-0.09034	0.9294	0.004424	0.02521	-0.1184
cg09609613	0.1532	-0.05763	0.6617	-0.01869	0.0167	-0.1632
cg09615760	0.05286	-0.0846	0.03595	-0.1213	0.01799	-0.1063
cg09640065	0.07032	-0.05179	0.7345	-0.01391	0.01829	-0.1119
cg09666444	0.06651	-0.08394	0.94	0.004205	0.03017	-0.1076
cg09689385	0.09835	-0.07449	0.7887	-0.01319	0.01291	-0.1211
cg09733719	0.06563	-0.0751	0.04516	-0.1215	0.03433	-0.0736
cg09741995	0.1496	-0.04883	0.01351	-0.1143	0.3146	-0.03264
cg09768680	0.02597	-0.1213	0.3929	-0.05073	0.03924	-0.1031
cg09781747	0.9137	-0.00432	0.8215	-0.01263	0.01386	-0.1084
cg09784070	0.417	0.02336	0.04374	-0.1039	0.9733	-0.001549
cg09799162	0.1248	-0.03181	0.03699	-0.1414	0.04687	-0.03583
cg09828339	0.6906	-0.01069	0.02269	-0.103	0.929	0.002867
cg09839654	0.05765	0.08753	0.8844	0.008664	0.01245	0.1357
cg09849036	0.08369	0.08508	0.5474	0.03261	0.03805	0.1073
cg09858826	0.08296	0.06472	0.4378	0.02973	0.02959	0.1087
cg09861299	0.02388	0.1017	0.8554	-0.008987	0.06989	0.07635
cg09886276	0.05055	-0.09144	0.06122	-0.08792	0.02771	-0.1033
cg09935994	0.04109	-0.1044	0.7136	-0.01655	0.07511	-0.09284
cg09988805	0.0387	-0.1221	0.2568	-0.03578	0.03999	-0.09248
cg09990943	0.0831	0.07725	0.9539	-0.003003	0.03684	0.1005
cg09994094	0.1136	0.06597	0.02474	0.1087	0.1102	0.0565
cg09999096	0.6468	-0.0298	0.09651	-0.1351	0.01204	-0.198
cg10008882	0.03732	0.1028	0.9295	-0.004896	0.05855	0.04899
cg10017964	0.95	0.00224	0.01339	-0.1519	0.6824	-0.01198
cg10027815	0.04721	-0.1017	0.579	-0.03004	0.06286	-0.07921
cg10049588	0.0118	-0.105	0.9015	-0.006205	0.01383	-0.0909
cg10068516	0.05979	-0.09799	0.7894	0.01234	0.01934	-0.1181
cg10070735	0.8474	0.007619	0.9475	-0.004011	0.03517	0.1132
cg10070915	0.268	0.04669	0.4196	0.03949	0.02888	0.1097

cg10081811	0.02635	-0.1096	0.2727	-0.04017	0.176	-0.05087
cg10119564	0.05978	-0.09293	0.19	-0.05682	0.03516	-0.1119
cg10137287	0.07958	0.07674	0.9356	0.005078	0.03224	0.1003
cg10233639	0.01715	-0.1133	0.2852	0.0419	0.5999	-0.02353
cg10257671	0.044	0.07546	0.3102	0.03864	0.008158	0.1127
cg10258033	0.03543	0.1034	0.373	0.06317	0.01398	0.1384
cg10263583	0.623	-0.01995	0.04815	-0.1021	0.2029	-0.04801
cg10294939	0.04596	-0.1251	0.1369	-0.09271	0.04826	-0.1339
cg10299128	0.2011	-0.02609	0.01254	-0.1176	0.1485	-0.0442
cg10314760	0.6414	0.01457	0.0465	-0.1187	0.2	0.04003
cg10334509	0.3984	-0.03084	0.028	-0.1004	0.575	-0.01638
cg10363337	0.04012	-0.1444	0.9038	-0.00955	0.3733	-0.04629
cg10374402	0.1258	-0.05829	0.02414	-0.1064	0.08194	-0.06033
cg10384201	0.04925	-0.1236	0.9176	0.007131	0.06837	-0.09779
cg10402316	0.007999	-0.09388	0.04418	-0.08151	0.01595	-0.107
cg10426372	0.1875	-0.06065	0.1357	-0.09107	0.03105	-0.102
cg10430994	0.2524	-0.04576	0.01981	-0.1189	0.4091	-0.02891
cg10440718	0.01927	-0.08514	0.01062	-0.1082	0.005098	-0.1242
cg10448258	0.09082	0.0441	0.01515	0.1048	0.00852	0.1397
cg10458554	0.07998	0.02865	0.01672	-0.115	0.7191	0.008131
cg10500719	0.02439	-0.1299	0.3006	-0.05927	0.7295	-0.02122
cg10502354	0.2598	-0.029	0.01741	-0.1038	0.1775	-0.03606
cg10502517	0.008107	-0.1085	0.07195	-0.06722	0.02137	-0.07193
cg10503767	0.1748	-0.05452	0.02605	-0.1425	0.2002	-0.06522
cg10504669	0.01968	0.1392	0.8663	0.009705	0.03839	0.1005
cg10516080	0.01141	-0.1283	0.2002	-0.04234	0.06704	-0.07653
cg10518811	0.02673	-0.1137	0.5253	-0.02693	0.08621	-0.07593
cg10532351	0.02573	0.1039	0.9304	0.005164	0.08677	0.06807
cg10554439	0.04634	-0.06666	0.01465	-0.1018	0.1325	-0.05286
cg10566610	0.01839	-0.07006	0.939	0.001229	0.006846	-0.1105
cg10592734	0.04808	-0.1045	0.9685	-0.002283	0.09154	-0.07469
cg10623530	0.09089	0.03698	0.2952	-0.03072	0.005207	0.1054
cg10637890	0.3412	-0.01627	0.01122	-0.1065	0.8659	-0.004431
cg10646447	0.4992	-0.01623	0.01369	-0.1286	0.1028	-0.06765
cg10673484	0.01602	-0.1098	0.1648	-0.06243	0.05152	-0.0529
cg10677105	0.04709	-0.1211	0.2646	-0.0816	0.3442	-0.06642
cg10686955	0.1978	-0.04435	0.04866	-0.1081	0.545	-0.01813
cg10687107	0.07894	-0.09865	0.9422	0.003723	0.04067	-0.107
cg10737307	0.01723	-0.1141	0.08963	-0.07287	0.09418	-0.0431
cg10813585	0.02412	0.1049	0.8528	0.006882	0.04915	0.08468
cg10832788	0.01497	-0.1097	0.7459	0.01303	0.1289	-0.06757
cg10837110	0.05797	0.08033	0.6187	-0.01871	0.01147	0.1282
cg10863300	0.6337	0.02067	0.1185	-0.08058	0.03364	-0.1137
cg10868567	0.1896	-0.03964	0.01734	-0.1063	0.1504	-0.05228
cg10870177	0.166	-0.03414	0.01505	-0.1157	0.05132	-0.06683
cg10877183	0.05229	-0.1187	0.08153	-0.1205	0.03555	-0.1432
cg10900075	0.3023	-0.02275	0.01579	-0.1315	0.174	-0.05102
cg10921001	0.02142	0.1135	0.8441	0.009216	0.1399	0.0393
cg10934670	0.02115	0.1253	0.8766	0.007074	0.012	0.1377
cg10935804	0.4115	-0.02827	0.04764	-0.1136	0.8642	0.009285

cg10942455	0.798	-0.006779	0.744	-0.01052	0.004976	-0.1092
cg10965596	0.9248	-0.003475	0.03699	-0.1032	0.5515	-0.02081
cg10990357	0.059	-0.07207	0.1381	-0.06469	0.007608	-0.1327
cg11013329	0.01643	0.1676	0.08559	0.1134	0.07132	0.1073
cg11024641	0.03683	-0.1053	0.4811	-0.04113	0.1119	-0.09046
cg11027529	0.3119	0.02335	0.01906	-0.1044	0.5077	0.0215
cg11063880	0.04801	-0.1162	0.2072	-0.07494	0.05607	-0.1199
cg11077103	0.03223	-0.06232	0.01416	-0.1133	0.02688	-0.06474
cg11078188	0.01479	-0.1107	0.3604	-0.02648	0.09135	-0.04174
cg11090384	0.02118	0.1242	0.4476	0.03013	0.1204	0.06279
cg11100465	0.1158	0.2505	0.08962	0.3583	0.04092	0.3242
cg11127770	0.01975	-0.1589	0.9874	-0.001091	0.07729	-0.08746
cg11140265	0.6655	-0.01423	0.03436	-0.1106	0.7609	-0.01005
cg11143438	0.6103	-0.01727	0.0187	-0.1165	0.5238	-0.02173
cg11191914	0.2034	-0.0505	0.5869	-0.01653	0.006351	-0.1211
cg11226661	0.02259	-0.103	0.07614	-0.0729	0.02381	-0.09494
cg11227979	0.04568	-0.2292	0.05789	-0.1719	0.1573	-0.1261
cg11237636	0.0479	-0.1187	0.9912	-0.0006376	0.1004	-0.08138
cg11242581	0.5595	-0.01319	0.048	-0.1087	0.4295	-0.01835
cg11252995	0.04709	-0.1282	0.2481	-0.08232	0.03852	-0.1702
cg11256152	0.03035	-0.1099	0.2386	-0.04006	0.06849	-0.06953
cg11264667	0.02872	0.1092	0.9978	-0.0002798	0.1137	0.07334
cg11290286	0.01005	-0.1888	0.8051	-0.01333	0.08026	-0.09859
cg11318129	0.007342	0.2307	0.262	0.06186	0.01664	0.148
cg11352430	0.02346	-0.1543	0.2109	-0.0698	0.01978	-0.14
cg11391912	0.0368	-0.1298	0.7126	-0.02335	0.3714	-0.04221
cg11425517	0.8671	0.01134	0.0409	-0.1321	0.579	-0.03371
cg11432900	0.03227	-0.1013	0.2514	-0.05262	0.1432	-0.07483
cg11435261	0.03295	-0.1043	0.2421	-0.06196	0.1538	-0.0603
cg11442337	0.02577	0.1245	0.5494	0.03352	0.07812	0.09331
cg11447377	0.008963	0.1076	0.9747	0.00151	0.1632	0.04702
cg11464053	0.2048	0.06284	0.5023	0.03896	0.03318	0.1056
cg11467177	0.04431	0.06047	0.5792	0.01518	0.02375	0.1044
cg11494508	0.06439	-0.07929	0.2968	-0.05718	0.02516	-0.1138
cg11500790	0.03947	-0.1457	0.09402	-0.1052	0.04334	-0.1112
cg11508593	0.0809	0.07133	0.8493	-0.01174	0.0285	0.1208
cg11539674	0.04542	-0.1078	0.9289	-0.004949	0.1012	-0.07642
cg11547764	0.08923	-0.108	0.9202	-0.0064	0.03972	-0.1132
cg11551901	0.9061	0.006415	0.04172	-0.1385	0.591	0.02367
cg11587848	0.09829	-0.06066	0.02711	-0.1047	0.2265	-0.04579
cg11602041	0.01349	0.1279	0.9996	-2.89E-05	0.01869	0.101
cg11617129	0.8468	-0.01065	0.04829	-0.1229	0.6342	-0.01943
cg11628864	0.02006	-0.1058	0.06946	-0.1036	0.1478	-0.05763
cg11633177	0.8279	-0.006747	0.04819	-0.1026	0.09083	-0.05266
cg11637718	0.03152	-0.1065	0.6768	0.01484	0.04185	-0.07226
cg11650585	0.5764	-0.01384	0.03739	-0.105	0.327	-0.02656
cg11676622	0.8413	-0.007062	0.02872	-0.1111	0.7754	-0.01062
cg11702543	0.47	-0.03178	0.03769	-0.1196	0.1877	-0.04738
cg11731173	0.8727	-0.005689	0.06024	-0.08026	0.008406	-0.1233
cg11739924	0.3216	0.04779	0.9828	0.001711	0.04652	0.1021

cg11755441	0.4207	-0.0384	0.2188	-0.09822	0.03475	-0.1277
cg11781676	0.04753	-0.0652	0.01369	-0.1165	0.7422	-0.01124
cg11787780	0.5135	-0.01992	0.02024	-0.1136	0.1427	-0.04617
cg11807559	0.5934	-0.02365	0.01358	-0.2201	0.3373	-0.05194
cg11830061	0.04749	-0.1023	0.9427	-0.004729	0.1647	-0.07881
cg11830721	0.008881	0.1209	0.6217	0.01718	0.008895	0.1151
cg11833983	0.3336	-0.02841	0.02012	-0.106	0.8315	-0.006618
cg11847964	0.02785	-0.1111	0.5008	-0.02084	0.214	-0.04328
cg11854563	0.01141	0.1062	0.8649	-0.007277	0.04284	0.07195
cg11854691	0.3462	-0.02384	0.07443	-0.07788	0.009027	-0.1139
cg11857253	0.5142	-0.02241	0.03003	-0.112	0.9239	-0.003253
cg11859594	0.03207	-0.0843	0.01827	-0.1035	0.071	-0.04818
cg11875405	0.1231	0.05637	0.04733	0.1212	0.1281	0.04932
cg11881146	0.1414	-0.05366	0.1199	-0.08592	0.0194	-0.1103
cg11904751	0.06079	-0.07139	0.03752	-0.0933	0.01321	-0.1822
cg11924169	0.6966	-0.01221	0.03861	-0.1144	0.9944	-0.0003644
cg11924931	0.3914	0.02482	0.01228	-0.1185	0.6479	0.01251
cg11925710	0.7885	0.01095	0.03888	-0.1014	0.9764	0.00151
cg11932719	0.8335	-0.004146	0.01956	-0.1051	0.7632	0.006367
cg11942332	0.987	0.0007939	0.03233	-0.1403	0.9698	-0.002229
cg11967527	0.9958	0.0002408	0.02898	-0.1276	0.1109	-0.06603
cg11974120	0.0254	-0.1071	0.2942	-0.05393	0.07414	-0.08827
cg11998420	0.1677	0.04848	0.06956	0.07372	0.01674	0.1134
cg12008447	0.04759	-0.1231	0.9956	-0.0003758	0.1609	-0.0746
cg12013591	0.02423	-0.1157	0.9181	0.01033	0.1023	-0.06875
cg12024649	0.0425	-0.08122	0.5049	-0.02238	0.009257	-0.1661
cg12025209	0.01359	-0.06841	0.07421	-0.0408	0.006801	-0.1105
cg12078642	0.1056	-0.06774	0.05643	-0.09267	0.03001	-0.1076
cg12081021	0.04778	-0.1191	0.03757	-0.124	0.03448	-0.1018
cg12097811	0.07554	-0.1039	0.08545	-0.1023	0.03338	-0.1133
cg12136497	0.2884	-0.05126	0.07904	-0.1074	0.04248	-0.1077
cg12146909	0.02366	-0.09004	0.0838	-0.06695	0.01576	-0.1115
cg12148123	0.3431	0.02833	0.0216	-0.134	0.7719	0.01315
cg12154842	0.05204	-0.1068	0.2183	-0.07847	0.02344	-0.1238
cg12168074	0.07012	-0.1295	0.9143	0.007267	0.04589	-0.1169
cg12171875	0.03125	-0.1729	0.6042	-0.03282	0.1352	-0.07545
cg12186771	0.04354	0.09646	0.1919	0.06125	0.03061	0.1015
cg12209615	0.06328	0.0442	0.02772	-0.1038	0.6102	0.01142
cg12234870	0.02515	-0.1109	0.07129	-0.09599	0.3888	-0.03812
cg12245637	0.117	-0.03799	0.02152	-0.1228	0.8901	0.00396
cg12253571	0.063	-0.09997	0.489	-0.03647	0.0353	-0.1112
cg12307982	0.07594	0.0489	0.04266	-0.1155	0.1001	0.06004
cg12308861	0.8847	-0.003977	0.0212	-0.1052	0.4482	-0.01917
cg12377222	0.01816	0.108	0.8852	-0.007707	0.1198	0.06571
cg12378285	0.03076	-0.1011	0.1541	-0.04754	0.01941	-0.08239
cg12428218	0.02578	-0.1097	0.9419	0.003773	0.03053	-0.1104
cg12454073	0.4775	-0.02418	0.04374	-0.1005	0.7889	-0.01062
cg12467098	0.15	0.06176	0.1992	0.06037	0.03606	0.1217
cg12467490	0.01643	-0.1052	0.5779	-0.01532	0.03293	-0.07545
cg12476230	0.03995	-0.108	0.1196	-0.04498	0.03747	-0.05679

cg12482993	0.02531	-0.1102	0.6793	-0.01844	0.03574	-0.0892
cg12486315	0.0149	-0.08426	0.3015	-0.03452	0.01624	-0.1063
cg12490773	0.006766	0.1101	0.1323	0.02988	0.01471	0.0643
cg12505658	0.0923	-0.05978	0.04206	-0.1002	0.1288	-0.0493
cg12508693	0.2741	-0.04186	0.04759	-0.1111	0.3536	-0.0514
cg12558059	0.05713	-0.08897	0.8929	0.006113	0.04137	-0.1079
cg12605529	0.03222	0.1063	0.6372	-0.02968	0.8266	0.01047
cg12622597	0.04876	-0.2088	0.9726	0.003671	0.3446	-0.08705
cg12629056	0.8922	0.007525	0.0444	-0.1633	0.9501	-0.004043
cg12643702	0.0198	0.103	0.7853	-0.01287	0.1102	0.04686
cg12653149	0.4739	-0.03262	0.04521	-0.1055	0.6473	-0.02131
cg12695915	0.07749	-0.05789	0.02001	-0.1236	0.03415	-0.07642
cg12708181	0.1783	-0.05037	0.0474	-0.1133	0.8725	-0.006104
cg12787787	0.02611	0.09457	0.2962	0.0368	0.01824	0.115
cg12788105	0.04498	-0.1013	0.6326	-0.02089	0.9723	-0.00145
cg12824282	0.2589	0.03456	0.02896	0.1119	0.4972	0.02164
cg12840850	0.1428	-0.05939	0.7135	-0.01844	0.02413	-0.1022
cg12854248	0.6028	-0.0156	0.04692	-0.1041	0.7302	-0.01737
cg12856865	0.8424	0.00639	0.04581	-0.1028	0.3378	-0.01966
cg12873350	0.04199	0.07422	0.08527	0.07744	0.02192	0.1024
cg12891810	0.04671	-0.1031	0.06665	-0.08488	0.05207	-0.09542
cg12903012	0.01172	0.1101	0.6064	0.01758	0.03312	0.0747
cg12929255	0.02543	0.1013	0.7987	0.01101	0.1142	0.06222
cg12958642	0.04021	0.1316	0.4813	0.05362	0.136	0.09397
cg12959488	0.08999	-0.03894	0.0474	-0.1024	0.1478	-0.03402
cg12962953	0.04048	-0.04663	0.01501	-0.1339	0.03594	-0.07604
cg12966202	0.02988	-0.06461	0.1159	-0.04991	0.008859	-0.1211
cg12967050	0.05553	0.07282	0.06614	0.05017	0.04263	0.1093
cg12982075	0.02147	-0.1372	0.1858	-0.07498	0.0307	-0.129
cg12988570	0.9765	0.001386	0.01416	-0.1573	0.9778	-0.001146
cg13053116	0.04974	-0.101	0.1395	-0.09049	0.1417	-0.06911
cg13097462	0.1688	-0.05885	0.2124	-0.05918	0.0244	-0.103
cg13098800	0.1627	-0.0332	0.01579	-0.1235	0.6274	-0.01469
cg13107864	0.1023	-0.04206	0.01746	-0.1012	0.09573	-0.0445
cg13111928	0.02274	0.08256	0.1259	0.05927	0.008868	0.1041
cg13146336	0.03439	-0.1007	0.6577	-0.01976	0.07134	-0.07761
cg13147462	0.04807	-0.1134	0.474	-0.03696	0.01658	-0.1178
cg13173809	0.005874	-0.1445	0.1269	-0.05607	0.01948	-0.1065
cg13175850	0.08777	-0.06939	0.02672	-0.134	0.09191	-0.07783
cg13195849	0.04509	-0.1054	0.7017	-0.01999	0.2248	-0.05246
cg13199010	0.04412	-0.1147	0.5513	-0.03175	0.3087	-0.05654
cg13201036	0.02351	-0.1079	0.1064	-0.1063	0.04893	-0.07386
cg13312035	0.04408	0.1058	0.365	-0.05332	0.07816	0.0817
cg13326063	0.3414	-0.03369	0.07126	-0.1139	0.02033	-0.1346
cg13329381	0.1372	0.07784	0.2166	0.07762	0.0423	0.1103
cg13347284	0.1142	0.09031	0.9063	0.008613	0.04613	0.1056
cg13364923	0.04674	-0.06051	0.4343	-0.02223	0.009564	-0.1091
cg13372513	0.02131	-0.1501	0.3745	-0.04267	0.1171	-0.09764
cg13372624	0.02127	-0.106	0.1331	-0.06498	0.2751	-0.04459
cg13376438	0.04876	-0.1008	0.9049	0.006993	0.1535	-0.07358

cg13390780	0.04662	-0.1267	0.9055	-0.008483	0.1027	-0.0873
cg13408940	0.04144	-0.07097	0.03182	-0.1032	0.04534	-0.07318
cg13418562	0.1514	-0.05421	0.01333	-0.1338	0.008872	-0.1213
cg13420803	0.04498	-0.107	0.4681	-0.04628	0.1947	-0.06909
cg13427189	0.02882	-0.1273	0.6731	0.02476	0.7126	0.02366
cg13430943	0.03289	-0.1028	0.6282	-0.02349	0.01153	-0.1203
cg13435220	0.06197	-0.03799	0.01627	-0.1277	0.3298	-0.03599
cg13442319	0.01489	-0.1085	0.2458	-0.04516	0.03377	-0.07819
cg13457515	0.02477	-0.1071	0.1313	-0.0658	0.03833	-0.09764
cg13458607	0.01192	-0.1188	0.2737	-0.03341	0.01029	-0.09761
cg13472610	0.8807	0.007999	0.04697	-0.1043	0.502	0.03236
cg13495667	0.02975	0.1463	0.1504	0.09276	0.04969	0.1308
cg13499708	0.4429	-0.03103	0.04043	-0.1299	0.8111	0.01174
cg13510487	0.03888	0.1029	0.2159	0.05687	0.1367	0.07507
cg13537590	0.03682	-0.1296	0.529	0.03968	0.05831	-0.09999
cg13551754	0.02979	0.1273	0.3214	0.0568	0.01457	0.1364
cg13557530	0.0357	-0.1066	0.05013	-0.1081	0.03308	-0.1123
cg13615770	0.9037	0.002762	0.02891	-0.1058	0.407	0.01433
cg13634600	0.5271	-0.01821	0.01901	-0.1317	0.2419	-0.04329
cg13634870	0.03863	-0.1663	0.9678	-0.004016	0.2473	-0.09291
cg13650176	0.03888	-0.1108	0.7596	-0.01675	0.4727	-0.0308
cg13650484	0.04994	-0.0613	0.2642	-0.03392	0.02036	-0.1008
cg13654183	0.8854	0.006873	0.03512	-0.1068	0.551	-0.02198
cg13678412	0.07003	-0.0587	0.1592	-0.07459	0.008859	-0.1974
cg13681768	0.04273	-0.108	0.7791	-0.01482	0.389	-0.05032
cg13681992	0.1821	0.05738	0.9355	0.00495	0.03312	0.1165
cg13692650	0.03051	-0.1011	0.2812	-0.04261	0.1318	-0.05655
cg13707189	0.03488	-0.1021	0.02155	-0.09495	0.06126	-0.0786
cg13708475	0.01569	-0.08278	0.01246	-0.1177	0.05729	-0.06768
cg13765685	0.01942	-0.1055	0.4645	-0.0284	0.3363	-0.03865
cg13775196	0.01478	0.1135	0.1261	0.04689	0.05849	0.06738
cg13775295	0.05231	-0.07812	0.03826	-0.1021	0.1498	-0.05806
cg13780911	0.01795	0.1105	0.8477	0.00778	0.04434	0.07174
cg13787252	0.2117	-0.05023	0.04618	-0.1438	0.02022	-0.09969
cg13800745	0.2257	-0.0487	0.03518	-0.1185	0.03789	-0.1027
cg13802789	0.2092	0.08072	0.8396	-0.01658	0.03364	0.1083
cg13805793	0.07541	-0.07952	0.08979	-0.08841	0.02904	-0.12
cg13814242	0.1481	-0.0612	0.1217	-0.07309	0.02498	-0.1064
cg13837502	0.07521	0.1139	0.2982	0.06743	0.02841	0.1542
cg13844500	0.03616	-0.1155	0.7394	0.01915	0.2508	-0.05397
cg13862258	0.798	-0.03595	0.5238	0.05122	0.01383	0.2631
cg13870005	0.04108	0.1168	0.4896	0.03272	0.03841	0.1115
cg13871684	0.07564	-0.06386	0.795	-0.0114	0.028	-0.1125
cg13881619	0.005874	-0.1321	0.4468	-0.02443	0.01519	-0.1365
cg13895148	0.1603	-0.03143	0.02574	-0.1127	0.1635	-0.04036
cg13896748	0.03681	0.1268	0.3917	0.04232	0.06737	0.09269
cg13899418	0.005874	-0.1469	0.4326	-0.02936	0.02364	-0.1081
cg13910601	0.9166	-0.003128	0.02219	-0.11	0.5686	-0.01743
cg13913589	0.2378	-0.04319	0.1967	-0.06167	0.03787	-0.1115
cg13914373	0.0377	0.09843	0.9223	-0.006797	0.03664	0.1007

cg13931531	0.029	-0.08489	0.1326	-0.06562	0.0263	-0.108
cg13939216	0.02681	0.1004	0.273	0.03684	0.01209	0.1011
cg13947691	0.167	0.07513	0.042	-0.1113	0.2047	0.06684
cg13978447	0.02252	0.08975	0.328	0.03589	0.01245	0.1045
cg13988448	0.01294	0.07373	0.02931	0.06438	0.006578	0.1039
cg14006031	0.4355	-0.01678	0.02791	-0.109	0.3602	-0.03063
cg14036868	0.03395	-0.1022	0.7398	0.01795	0.02979	-0.09238
cg14038077	0.02582	-0.1028	0.4773	-0.0327	0.1298	-0.08757
cg14085715	0.09844	0.05428	0.8163	0.01076	0.01983	0.1044
cg14089418	0.7909	0.01304	0.04212	-0.1003	0.2634	-0.05224
cg14102721	0.04645	-0.08629	0.2551	-0.05607	0.01747	-0.1124
cg14114719	0.0757	-0.0715	0.03352	-0.1119	0.8421	-0.008091
cg14129328	0.3636	-0.04621	0.5119	-0.04023	0.01907	-0.126
cg14167415	0.04965	-0.1329	0.8906	-0.01043	0.04198	-0.1406
cg14175802	0.3446	-0.02473	0.01848	-0.1023	0.2495	-0.03084
cg14178352	0.02448	-0.08166	0.01078	-0.1561	0.4393	-0.02191
cg14185509	0.09404	-0.05731	0.2854	-0.04303	0.01414	-0.1099
cg14194576	0.8912	-0.007561	0.2748	-0.05493	0.04097	-0.1011
cg14222432	0.5119	-0.02818	0.0474	-0.1057	0.9587	0.002537
cg14231565	0.02142	-0.1275	0.2608	-0.05329	0.2055	-0.05658
cg14233178	0.03756	0.1823	0.9605	-0.005225	0.1527	0.09361
cg14265604	0.04474	0.2429	0.5923	0.06688	0.06567	0.2199
cg14277161	0.01426	-0.07769	0.05414	-0.05662	0.00704	-0.1093
cg14281039	0.06528	0.06519	0.6938	0.017	0.0218	0.107
cg14281821	0.1127	-0.06682	0.6327	-0.02622	0.0414	-0.1048
cg14282698	0.3097	-0.02185	0.01339	-0.1126	0.3359	-0.02747
cg14332819	0.7472	-0.007493	0.01122	-0.1097	0.2275	-0.03426
cg14346311	0.439	-0.02919	0.03298	-0.1446	0.128	-0.04989
cg14354292	0.05556	0.1901	0.08634	0.2	0.03891	0.2044
cg14367995	0.005629	-0.1078	0.6638	-0.01005	0.006846	-0.07439
cg14372606	0.258	0.03889	0.02024	0.1053	0.9362	0.002702
cg14373914	0.04433	-0.06998	0.0345	-0.1002	0.04059	-0.08021
cg14399284	0.3246	-0.04634	0.03185	-0.1327	0.603	-0.01951
cg14418119	0.007101	-0.1086	0.1559	-0.03108	0.02359	-0.06151
cg14427255	0.7247	-0.01195	0.04548	-0.1115	0.3024	-0.03507
cg14435083	0.09858	0.05477	0.01596	-0.1274	0.6895	-0.01334
cg14444798	0.5874	-0.02155	0.03162	-0.1079	0.04877	-0.0714
cg14445707	0.6019	-0.01304	0.01358	-0.1025	0.1287	-0.03147
cg14446979	0.06343	0.1994	0.06864	0.2713	0.04086	0.2269
cg14456873	0.1578	-0.06422	0.9971	0.0002486	0.03574	-0.1117
cg14484792	0.04969	-0.08911	0.08889	-0.07054	0.01552	-0.1027
cg14485083	0.08056	-0.04639	0.01358	-0.1035	0.01755	-0.0808
cg14505104	0.009995	0.1118	0.8773	0.009708	0.2833	0.0431
cg14506716	0.02996	-0.1245	0.7299	-0.01721	0.09796	-0.08199
cg14506821	0.03391	-0.1193	0.04183	-0.1131	0.07082	-0.08791
cg14562441	0.9557	-0.001198	0.04018	-0.1109	0.5731	-0.01705
cg14565417	0.005874	0.1289	0.3445	0.03666	0.007946	0.1274
cg14575602	0.0245	0.08925	0.8031	0.01079	0.008049	0.1197
cg14581997	0.0205	-0.1505	0.9507	-0.004153	0.04137	-0.1158
cg14590644	0.01578	-0.1121	0.01489	-0.1159	0.0171	-0.12

cg14601416	0.04272	-0.05481	0.01528	-0.1072	0.03035	-0.05853
cg14622659	0.03662	-0.04777	0.1547	-0.04642	0.01264	-0.1118
cg14626381	0.2087	0.04114	0.999	-6.96E-05	0.046	0.102
cg14628993	0.0254	-0.1014	0.1238	-0.06977	0.03515	-0.09585
cg14646168	0.3815	0.03054	0.04109	-0.1044	0.3603	-0.03495
cg14672496	0.04783	0.1045	0.5321	0.03566	0.04813	0.1049
cg14683962	0.03867	-0.114	0.1055	-0.07119	0.05039	-0.08386
cg14704980	0.1312	-0.03721	0.0295	-0.1062	0.2122	-0.04398
cg14728174	0.04077	-0.1059	0.5375	0.0267	0.1435	-0.06485
cg14734841	0.04085	-0.1019	0.4167	-0.03643	0.385	-0.03847
cg14767228	0.02607	-0.102	0.09505	-0.09599	0.05696	-0.09202
cg14768930	0.07363	-0.06261	0.03411	-0.1351	0.0384	-0.08284
cg14770814	0.01167	-0.1052	0.7307	-0.01701	0.03668	-0.06166
cg14779509	0.02568	0.1107	0.6904	0.02315	0.07676	0.07411
cg14833461	0.3463	-0.0417	0.09069	-0.09105	0.03419	-0.1124
cg14868764	0.01943	-0.2863	0.8174	-0.03209	0.1191	-0.1457
cg14871290	0.0811	-0.067	0.03512	-0.1243	0.1507	-0.04217
cg14878305	0.2185	-0.03213	0.0177	-0.1003	0.9225	0.003752
cg14881187	0.03801	0.1157	0.306	0.05142	0.01609	0.1551
cg14882904	0.03015	0.08092	0.2375	0.03594	0.00842	0.1211
cg14913512	0.9064	-0.003625	0.03615	-0.1242	0.05504	-0.09248
cg14922325	0.1244	-0.06053	0.0715	-0.07877	0.02186	-0.1032
cg14928444	0.01757	0.09622	0.1976	0.04705	0.01758	0.1003
cg14933246	0.1186	-0.08733	0.3552	-0.06329	0.0475	-0.1334
cg14990478	0.7493	-0.01178	0.03143	-0.1236	0.03616	-0.09244
cg15014137	0.6486	0.02039	0.1385	-0.07253	0.01907	0.1038
cg15022523	0.3022	-0.04897	0.1955	-0.07387	0.04936	-0.1048
cg15027610	0.1606	-0.05279	0.6145	-0.0264	0.03025	-0.1167
cg15029698	0.02454	-0.1003	0.9101	-0.003743	0.2549	-0.03436
cg15041691	0.01359	-0.1108	0.8684	0.008181	0.6245	-0.02155
cg15051408	0.178	-0.06565	0.1419	-0.05126	0.01946	-0.1335
cg15067773	0.1829	-0.06406	0.03377	-0.1273	0.2787	-0.04737
cg15076259	0.05851	-0.09661	0.2686	-0.04461	0.01614	-0.1071
cg15115984	0.03813	-0.06427	0.88	-0.005591	0.01261	-0.1093
cg15154628	0.04682	-0.07569	0.02474	-0.1153	0.3059	-0.04028
cg15173629	0.03412	0.1096	0.2534	0.05414	0.2488	0.06162
cg15195990	0.009995	-0.1205	0.1073	-0.06886	0.05724	-0.09815
cg15208718	0.04576	-0.1453	0.4466	0.0462	0.1868	-0.08403
cg15216497	0.02752	0.1055	0.1978	0.05128	0.05777	0.09342
cg15222045	0.09788	-0.07135	0.7522	0.01507	0.03433	-0.1042
cg15227848	0.1745	-0.0539	0.01333	-0.1739	0.2317	-0.06282
cg15233880	0.03988	0.1115	0.1792	0.06078	0.02897	0.1131
cg15254238	0.02306	-0.1027	0.6702	-0.0162	0.0842	-0.05597
cg15257791	0.0222	-0.1005	0.3212	-0.03497	0.1162	-0.0581
cg15266508	0.01718	-0.1012	0.1214	-0.06594	0.02625	-0.08937
cg15320998	0.2432	-0.03181	0.03613	-0.1029	0.3311	-0.02514
cg15332173	0.5644	-0.01243	0.0146	-0.1011	0.213	-0.0392
cg15343322	0.01835	-0.105	0.6337	-0.01728	0.04179	-0.04626
cg15352537	0.3	-0.01797	0.02206	-0.105	0.224	-0.02631
cg15380432	0.005137	-0.1003	0.02894	-0.04284	0.005224	-0.09105

cg15408326	0.02035	-0.1422	0.1508	-0.05991	0.02731	-0.1107
cg15413849	0.2598	-0.05137	0.4474	-0.03151	0.04321	-0.1117
cg15441656	0.107	0.06513	0.08192	0.06411	0.01007	0.1219
cg15444831	0.2517	-0.0358	0.3452	-0.0407	0.03931	-0.1134
cg15448159	0.04368	-0.07398	0.05788	-0.08434	0.01833	-0.129
cg15479754	0.3997	-0.02534	0.01528	-0.128	0.1606	-0.05466
cg15481741	0.09781	-0.0809	0.03047	-0.1164	0.07673	-0.08621
cg15508393	0.2434	-0.0353	0.0324	-0.104	0.9247	0.003486
cg15555383	0.03171	0.1189	0.5669	0.02748	0.03101	0.1089
cg15584547	0.03623	-0.1243	0.1551	-0.09076	0.153	-0.06494
cg15601071	0.1107	-0.07591	0.02155	-0.1172	0.1313	-0.05989
cg15622493	0.07919	-0.09975	0.09093	-0.156	0.03057	-0.1616
cg15623687	0.02971	-0.1029	0.6318	0.01835	0.1474	-0.05346
cg15646593	0.04984	-0.1156	0.8498	-0.01278	0.2117	-0.06846
cg15660962	0.02987	0.09289	0.5166	0.02632	0.00852	0.1293
cg15671350	0.01596	-0.1383	0.3997	-0.04763	0.101	-0.07557
cg15692041	0.4792	0.02314	0.04954	-0.106	0.9308	-0.005471
cg15726123	0.04153	-0.112	0.6698	-0.02436	0.05025	-0.1031
cg15727398	0.006724	-0.08096	0.03515	-0.04517	0.005687	-0.1154
cg15748122	0.4069	-0.02538	0.04053	-0.1009	0.2739	-0.04012
cg15761231	0.08723	-0.05884	0.2245	-0.04737	0.008158	-0.1192
cg15790205	0.09181	-0.06595	0.6157	-0.02202	0.01138	-0.1143
cg15801640	0.04592	-0.1049	0.3147	0.03181	0.08511	-0.07481
cg15804259	0.4305	0.02743	0.02865	-0.1044	0.09353	-0.05731
cg15825553	0.1103	-0.05287	0.03766	-0.1031	0.2767	-0.02955
cg15829499	0.7973	-0.007519	0.03237	-0.1255	0.03403	-0.07471
cg15830081	0.1329	-0.04575	0.8645	0.008815	0.01802	-0.1086
cg15833019	0.7157	-0.01735	0.8208	-0.01595	0.02774	0.1078
cg15845156	0.02766	-0.104	0.05675	-0.0819	0.0881	-0.06151
cg15867829	0.0454	-0.05375	0.4106	-0.01533	0.004083	-0.1082
cg15870552	0.09302	0.1125	0.9679	0.003389	0.04959	0.1102
cg15892650	0.03038	-0.1685	0.4811	-0.05294	0.445	-0.04925
cg15946126	0.1213	-0.05735	0.01515	-0.1204	0.2557	-0.03513
cg15959363	0.8577	0.007206	0.03247	-0.1028	0.784	-0.01271
cg15965986	0.2683	0.04966	0.4708	0.04144	0.03135	0.1308
cg15993296	0.04257	-0.1116	0.849	-0.01104	0.1459	-0.07056
cg15998596	0.01648	0.1113	0.3048	0.02235	0.02563	0.06931
cg16024058	0.008927	0.1048	0.873	-0.007165	0.009517	0.0782
cg16028965	0.7607	0.01004	0.4076	-0.02868	0.02707	-0.1261
cg16043943	0.943	-0.005063	0.3096	-0.04438	0.02988	0.166
cg16057788	0.01177	-0.1015	0.3549	-0.02644	0.02717	-0.07649
cg16058493	0.01228	-0.1243	0.4431	-0.03068	0.0163	-0.105
cg16082682	0.1103	-0.05258	0.02587	-0.1046	0.1402	-0.03893
cg16091500	0.0196	0.1197	0.9215	-0.005549	0.9287	0.003971
cg16140794	0.8081	-0.01329	0.03017	-0.1422	0.7109	-0.01907
cg16144294	0.02335	-0.1151	0.6957	0.01999	0.07176	-0.08925
cg16170919	0.1222	-0.06447	0.8437	-0.01118	0.03509	-0.1161
cg16172587	0.119	-0.0906	0.8198	0.013	0.0441	-0.1151
cg16199914	0.5938	-0.01098	0.004388	-0.1659	0.0862	-0.04072
cg16206564	0.5006	-0.02331	0.04526	-0.1202	0.3376	-0.03472

cg16214654	0.02371	-0.1064	0.2789	-0.05014	0.06473	-0.08003
cg16220369	0.1813	0.04552	0.006942	-0.1336	0.8386	0.005509
cg16223899	0.04018	0.1397	0.9021	0.008229	0.0567	0.1194
cg16236736	0.06553	0.1243	0.206	0.08654	0.02357	0.1632
cg16243083	0.08821	-0.06522	0.1636	-0.05775	0.01734	-0.1209
cg16245191	0.1474	-0.06271	0.5355	0.02715	0.04391	-0.1038
cg16261823	0.8504	0.00614	0.02237	-0.1016	0.5838	-0.01757
cg16277040	0.06157	-0.1072	0.02212	-0.1555	0.02431	-0.1225
cg16291637	0.04568	-0.1305	0.2375	-0.07402	0.3627	-0.06423
cg16310458	0.145	-0.0386	0.01901	-0.1164	0.06627	-0.06406
cg16313875	0.08988	-0.1069	0.7509	-0.01841	0.02708	-0.1109
cg16316183	0.1389	0.07413	0.7677	-0.01951	0.03592	0.1117
cg16333323	0.5769	0.02875	0.04581	-0.1176	0.7182	0.02027
cg16348233	0.2131	-0.05361	0.01339	-0.1596	0.02646	-0.1023
cg16362222	0.01959	-0.1017	0.6753	-0.0266	0.7325	-0.01773
cg16391727	0.01385	0.1466	0.5069	-0.03978	0.02826	0.1249
cg16393844	0.03795	0.1075	0.8803	0.009884	0.01398	0.1512
cg16395911	0.7004	-0.02035	0.02472	-0.1135	0.4786	-0.02295
cg16403509	0.6905	-0.01018	0.04051	-0.1116	0.06327	-0.05733
cg16429261	0.2089	-0.05435	0.02364	-0.1354	0.3087	-0.03787
cg16457489	0.1168	-0.04463	0.03465	-0.1235	0.09249	-0.05771
cg16457652	0.02231	-0.1181	0.1532	-0.05606	0.06033	-0.07854
cg16469636	0.05213	-0.05213	0.01361	-0.1191	0.06103	-0.04524
cg16490439	0.1231	-0.06046	0.03152	-0.145	0.1909	-0.05542
cg16547382	0.7959	-0.009756	0.02767	-0.1188	0.7435	-0.01324
cg16550359	0.03078	0.1561	0.3428	0.07141	0.0922	0.09527
cg16552406	0.2635	-0.03557	0.02121	-0.1039	0.2341	-0.03995
cg16583884	0.2605	-0.05309	0.1052	-0.114	0.02434	-0.1181
cg16599631	0.8753	-0.005826	0.8883	-0.005823	0.02553	-0.1038
cg16624729	0.432	-0.02687	0.03185	-0.1069	0.05449	-0.07211
cg16631618	0.2812	0.04667	0.6785	0.02318	0.0499	0.1001
cg16633394	0.01006	0.1211	0.0864	0.06634	0.0145	0.1041
cg16643177	0.2272	-0.03895	0.04592	-0.109	0.5992	0.01441
cg16650906	0.02026	-0.07463	0.07978	-0.04554	0.007416	-0.1036
cg16672922	0.02794	0.1681	0.8807	-0.008749	0.01847	0.1697
cg16696039	0.03954	0.1221	0.2311	0.07543	0.02093	0.1444
cg16699128	0.02882	-0.1126	0.6982	0.01752	0.1661	-0.06189
cg16717793	0.9624	-0.002267	0.02519	-0.1522	0.7513	-0.01574
cg16730700	0.005874	0.1018	0.1362	0.03083	0.0111	0.07937
cg16745246	0.1116	0.1007	0.6962	0.03039	0.04423	0.1244
cg16763840	0.2563	-0.05384	0.0885	-0.1013	0.03994	-0.1004
cg16777477	0.08437	-0.07108	0.4626	0.03032	0.04284	-0.1072
cg16781548	0.02851	-0.1263	0.8725	-0.008412	0.02497	-0.1078
cg16790390	0.4407	-0.01768	0.01961	-0.1169	0.5213	-0.01688
cg16803837	0.02823	0.0946	0.08935	0.08733	0.01109	0.117
cg16819400	0.3776	-0.03475	0.7035	0.01844	0.03196	-0.1107
cg16848009	0.04668	-0.1061	0.2443	-0.04862	0.3565	-0.03692
cg16877697	0.1305	-0.06265	0.5496	-0.02574	0.01988	-0.1006
cg16884491	0.048	-0.1124	0.293	-0.05402	0.1401	-0.07059
cg16907766	0.03812	0.1405	0.8406	0.01354	0.1145	0.08463

cg16908763	0.3711	0.02387	0.0345	0.1018	0.8577	0.009037
cg16915469	0.04971	0.1322	0.4489	-0.05	0.02489	0.1421
cg17009760	0.03623	-0.1175	0.5406	-0.03726	0.05942	-0.1129
cg17010737	0.0966	-0.03185	0.8581	-0.004994	0.006846	-0.1077
cg17031475	0.9718	0.002096	0.9407	0.006186	0.04302	0.1077
cg17031631	0.1159	-0.04776	0.01906	-0.1225	0.2457	-0.03651
cg17047106	0.6223	-0.01574	0.0234	-0.1085	0.3445	-0.03208
cg17081914	0.07961	-0.08149	0.7837	-0.01153	0.02679	-0.1075
cg17108237	0.008107	0.1018	0.126	0.04269	0.3293	0.03131
cg17117557	0.02126	-0.1054	0.4924	-0.02078	0.06325	-0.06866
cg17130474	0.03295	-0.1042	0.0898	-0.07376	0.9717	0.001947
cg17160906	0.05258	-0.05211	0.2316	-0.03567	0.01394	-0.1118
cg17167021	0.01487	-0.1049	0.3841	-0.02724	0.04153	-0.1206
cg17183213	0.03121	-0.102	0.07944	-0.09806	0.07111	-0.07298
cg17198663	0.01435	-0.1168	0.01326	-0.107	0.03042	-0.06506
cg17200325	0.6274	-0.01657	0.02637	-0.1262	0.482	-0.02579
cg17201721	0.4786	-0.02594	0.04887	-0.1016	0.6494	-0.01622
cg17202604	0.04924	-0.0645	0.677	0.01848	0.0141	-0.1254
cg17220690	0.08506	0.117	0.7707	0.02265	0.03363	0.1277
cg17232254	0.4209	-0.01398	0.01246	-0.1026	0.9561	0.00137
cg17272168	0.1616	-0.04218	0.01616	-0.1053	0.169	-0.0378
cg17273837	0.1735	0.06277	0.6813	-0.0189	0.03695	0.1032
cg17287034	0.206	-0.07856	0.3867	-0.04999	0.0446	-0.1405
cg17307051	0.02726	-0.132	0.9059	0.007143	0.1113	-0.07384
cg17314520	0.02126	-0.1052	0.2694	-0.0521	0.4361	-0.02372
cg17346231	0.2186	-0.03424	0.01906	-0.1056	0.3955	-0.01664
cg17348578	0.02748	0.1085	0.4478	0.06404	0.149	0.07815
cg17349025	0.02089	-0.1268	0.2547	-0.04894	0.02342	-0.09689
cg17363439	0.08654	-0.07834	0.8842	0.007442	0.03669	-0.103
cg17371170	0.5899	-0.02635	0.01894	-0.1437	0.8157	0.0117
cg17406317	0.02351	-0.1378	0.9474	-0.00397	0.05837	-0.104
cg17415167	0.01141	-0.07734	0.906	0.00321	0.004976	-0.1085
cg17415486	0.1979	0.04886	0.4282	-0.03742	0.0179	0.116
cg17421046	0.697	-0.01301	0.02968	-0.1008	0.1416	-0.04114
cg17422176	0.8625	-0.005072	0.01907	-0.1091	0.8638	-0.003451
cg17439770	0.1699	-0.0401	0.01746	-0.1416	0.5007	-0.0158
cg17443668	0.9664	-0.001288	0.01305	-0.1008	0.5837	-0.01445
cg17444751	0.2783	-0.04606	0.0282	-0.1133	0.1617	-0.06491
cg17464298	0.07329	-0.1295	0.5274	-0.0504	0.03841	-0.1631
cg17467154	0.05117	0.07496	0.4286	-0.02958	0.03264	0.1098
cg17499682	0.08934	-0.06413	0.0322	-0.1079	0.2277	-0.04763
cg17506887	0.318	-0.04233	0.4732	0.04225	0.03444	-0.1028
cg17554685	0.184	0.06538	0.1097	0.07722	0.03564	0.1047
cg17560785	0.05229	-0.08975	0.04549	-0.1106	0.1575	-0.05067
cg17588094	0.02797	-0.1017	0.5164	-0.02725	0.05614	-0.08245
cg17589580	0.04643	0.1197	0.8504	-0.01333	0.2723	0.05758
cg17596409	0.01343	-0.1125	0.5568	-0.01991	0.1396	-0.05185
cg17600630	0.0314	-0.1024	0.2346	-0.05412	0.05525	-0.08067
cg17619619	0.6521	0.01442	0.03643	-0.1032	0.6433	0.01343
cg17644279	0.02206	-0.08681	0.04958	-0.07439	0.01321	-0.1124

cg17658303	0.01321	-0.1028	0.4095	-0.01686	0.006986	-0.09744
cg17658731	0.02382	-0.1021	0.2682	-0.04102	0.1508	-0.04759
cg17702689	0.04429	0.1339	0.2365	0.08769	0.06976	0.122
cg17705814	0.1166	-0.07211	0.03178	-0.1603	0.02882	-0.09722
cg17727413	0.01268	0.0914	0.6444	-0.01699	0.01321	0.1064
cg17734022	0.4363	-0.0245	0.01465	-0.1473	0.1157	-0.04192
cg17738213	0.01662	-0.1367	0.09858	-0.07813	0.05094	-0.08871
cg17760881	0.02509	0.1042	0.854	-0.009379	0.01346	0.1439
cg17787321	0.08887	-0.04713	0.01196	-0.1076	0.06519	-0.05944
cg17823769	0.07411	0.1216	0.9598	0.004293	0.04446	0.1365
cg17836195	0.4297	0.02756	0.0324	-0.1459	0.8664	0.00721
cg17847074	0.9214	-0.002816	0.01746	-0.1005	0.02911	-0.06799
cg17859594	0.9783	0.000995	0.02896	-0.1473	0.3507	-0.0317
cg17906152	0.01044	-0.1024	0.069	-0.05934	0.002259	-0.1464
cg18036763	0.03815	-0.1504	0.6749	-0.03382	0.03433	-0.1416
cg18062044	0.01138	-0.09655	0.06689	-0.05593	0.003262	-0.1082
cg18087023	0.0605	-0.06961	0.02605	-0.1052	0.06043	-0.05764
cg18089906	0.03745	-0.08117	0.1972	-0.03368	0.01332	-0.103
cg18093151	0.6252	-0.0178	0.03005	-0.143	0.3985	-0.03383
cg18135337	0.03663	-0.1016	0.9807	-0.001589	0.02646	-0.09061
cg18141431	0.01282	-0.1187	0.198	-0.04962	0.3282	-0.03479
cg18144076	0.07593	-0.06682	0.01596	-0.1375	0.0552	-0.07278
cg18159794	0.5269	0.02533	0.3439	0.04481	0.02524	0.1008
cg18169835	0.1259	0.08418	0.8436	0.01351	0.04169	0.1027
cg18233217	0.02126	0.1438	0.8302	0.01294	0.05609	0.1341
cg18265665	0.6975	-0.01442	0.03318	-0.1114	0.3814	-0.03583
cg18273401	0.02673	-0.1272	0.973	-0.002297	0.246	-0.06914
cg18315831	0.03568	0.1044	0.4628	-0.03386	0.1686	0.0725
cg18317199	0.02621	-0.1164	0.117	-0.05779	0.1017	-0.06855
cg18323984	0.05307	-0.1085	0.1143	-0.09509	0.01584	-0.1334
cg18335670	0.02736	0.1231	0.8329	0.01261	0.01613	0.1395
cg18344020	0.04763	0.1505	0.6322	-0.01519	0.9364	0.004192
cg18354248	0.01557	-0.1219	0.5427	-0.03024	0.2323	-0.04191
cg18355137	0.02198	-0.1296	0.794	-0.01427	0.01641	-0.1311
cg18360994	0.04093	-0.1095	0.9117	0.006944	0.2407	-0.06054
cg18366199	0.09493	-0.0748	0.3197	-0.03297	0.01778	-0.1138
cg18369288	0.02127	-0.08992	0.9124	-0.004431	0.008818	-0.1012
cg18377014	0.4022	-0.1061	0.6864	0.02219	0.04368	0.1048
cg18395632	0.3609	0.02894	0.5601	-0.0202	0.009127	0.1155
cg18412984	0.01494	-0.1065	0.1561	-0.05183	0.01016	-0.1004
cg18420846	0.01573	-0.1066	0.2259	-0.04638	0.09937	-0.0568
cg18440027	0.0551	-0.06487	0.3523	-0.02819	0.01009	-0.1025
cg18466514	0.9342	-0.002821	0.0402	-0.1009	0.7435	0.009876
cg18493113	0.08858	0.07952	0.9445	-0.00436	0.02188	0.1181
cg18512357	0.1003	-0.08976	0.9385	0.006641	0.02572	-0.1266
cg18522626	0.03171	-0.1131	0.03381	-0.1456	0.1535	-0.078
cg18541453	0.006766	-0.1379	0.1292	-0.05674	0.02388	-0.08375
cg18587985	0.03462	0.117	0.2741	0.06464	0.02483	0.1338
cg18605120	0.04157	-0.0618	0.0804	-0.05673	0.009127	-0.1078
cg18647768	0.02515	0.1311	0.9171	0.006502	0.2937	0.04225

cg18657917	0.06578	-0.07947	0.01358	-0.1337	0.02263	-0.09088
cg18658709	0.05317	0.07508	0.3939	0.03529	0.02935	0.1039
cg18676273	0.02418	-0.1072	0.3177	-0.03566	0.03703	-0.083
cg18679869	0.02276	0.1028	0.5908	0.01971	0.05881	0.07757
cg18693051	0.04149	-0.07955	0.02961	-0.1087	0.4171	-0.02552
cg18700820	0.1677	-0.08813	0.04643	-0.1618	0.3031	-0.05848
cg18735451	0.1506	-0.0674	0.5803	-0.03593	0.04343	-0.1055
cg18737789	0.9495	-0.002221	0.02874	-0.1081	0.08721	-0.05701
cg18767244	0.7513	-0.01998	0.03211	-0.1845	0.8576	-0.01368
cg18770029	0.1407	-0.05882	0.09933	-0.0764	0.02346	-0.1195
cg18798487	0.07205	-0.049	0.01555	-0.1016	0.02601	-0.06509
cg18806980	0.1248	-0.0477	0.01848	-0.1122	0.02381	-0.08446
cg18814142	0.9037	-0.003577	0.02076	-0.102	0.6192	-0.0119
cg18820295	0.01497	0.1316	0.963	0.003694	0.08021	0.1041
cg18848528	0.03804	0.107	0.5074	0.02927	0.2572	0.0571
cg18883726	0.0254	-0.1057	0.4029	-0.04146	0.02909	-0.08692
cg18908062	0.04157	-0.08866	0.02461	-0.1062	0.02345	-0.09725
cg18943803	0.07305	0.09148	0.8987	-0.008611	0.04257	0.1057
cg18951919	0.1191	-0.08584	0.8811	-0.01151	0.02448	-0.1141
cg18955863	0.03834	-0.1385	0.7294	-0.01726	0.1219	-0.08503
cg18974117	0.01641	0.1178	0.7743	0.01355	0.02983	0.07205
cg18987685	0.05948	-0.08502	0.9224	0.004569	0.01332	-0.1134
cg18990874	0.01795	-0.1097	0.02973	-0.1048	0.1113	-0.05664
cg19000871	0.03269	-0.1667	0.9856	-0.001676	0.09305	-0.1092
cg19014792	0.02998	-0.1011	0.9706	0.001765	0.02801	-0.094
cg19024161	0.01251	0.1004	0.9247	-0.00468	0.06402	0.08563
cg19064941	0.1487	-0.0807	0.03374	-0.1481	0.06341	-0.1274
cg19066064	0.06249	-0.06192	0.08273	-0.05828	0.02358	-0.1196
cg19066589	0.1219	-0.08451	0.04214	-0.125	0.4136	-0.04253
cg19103770	0.2208	-0.03541	0.5468	-0.02684	0.01599	-0.1192
cg19124486	0.62	-0.02556	0.7914	-0.02326	0.03684	-0.1476
cg19128882	0.07043	-0.0828	0.395	-0.03459	0.02174	-0.1409
cg19129350	0.03889	-0.1081	0.5257	-0.03682	0.245	-0.05926
cg19133443	0.008202	0.155	0.5718	0.02518	0.008042	0.1383
cg19160955	0.3324	0.03478	0.0187	-0.1131	0.5087	0.01892
cg19172703	0.03313	-0.1107	0.761	0.01666	0.7068	0.0201
cg19179168	0.3226	-0.03019	0.02073	-0.1469	0.9564	0.003108
cg19188377	0.0453	0.08911	0.9493	-0.003171	0.02486	0.1282
cg19226080	0.0365	-0.1011	0.1448	-0.06392	0.3475	-0.04187
cg19227679	0.01227	-0.1065	0.03752	-0.05472	0.00461	-0.09905
cg19265606	0.0879	0.0588	0.9777	0.002079	0.02116	0.1145
cg19265886	0.02843	-0.08015	0.4115	-0.03332	0.0165	-0.1079
cg19268720	0.0175	0.1271	0.1407	0.07338	0.0196	0.1079
cg19269678	0.08392	-0.05058	0.01416	-0.1101	0.5109	-0.01803
cg19275526	0.3815	0.04128	0.7223	-0.0174	0.02884	0.1169
cg19303728	0.04399	-0.1441	0.7362	-0.02227	0.05495	-0.05253
cg19326244	0.01527	0.1139	0.6815	0.01682	0.05248	0.08005
cg19353326	0.02762	-0.09646	0.03701	-0.09646	0.03421	-0.1023
cg19399100	0.0158	-0.13	0.04125	-0.08842	0.00852	-0.1097
cg19413251	0.03175	-0.1141	0.0626	-0.08648	0.1452	-0.07547

cg19417833	0.549	0.02056	0.02485	-0.1128	0.8916	-0.006733
cg19424175	0.02101	0.1039	0.9415	-0.005377	0.03785	0.07374
cg19437793	0.005137	-0.1075	0.03699	-0.06903	0.01308	-0.07043
cg19445082	0.5094	-0.02483	0.02789	-0.1249	0.5024	-0.02669
cg19447020	0.01496	0.1073	0.4004	-0.03153	0.03619	0.09718
cg19449934	0.02118	0.07344	0.5921	0.01698	0.009823	0.1027
cg19450840	0.315	0.03362	0.06949	-0.081	0.03192	0.1052
cg19452011	0.02565	0.1429	0.6904	-0.01951	0.0194	0.1325
cg19475988	0.07656	-0.08923	0.7634	-0.01621	0.04089	-0.1024
cg19501536	0.03273	0.08324	0.04695	0.08107	0.03407	0.1268
cg19503143	0.03508	0.1184	0.9918	-0.0006531	0.1427	0.06841
cg19504888	0.882	0.0123	0.69	0.03627	0.04684	0.1365
cg19510150	0.5088	-0.01588	0.01358	-0.1073	0.6286	-0.01054
cg19529596	0.1132	-0.04999	0.6761	-0.01684	0.01286	-0.1143
cg19535507	0.02515	-0.1048	0.1258	-0.06799	0.05651	-0.08561
cg19557713	0.198	0.05351	0.3167	0.048	0.03005	0.1117
cg19564816	0.05976	-0.05471	0.01746	-0.1108	0.05606	-0.04909
cg19570759	0.1924	-0.0763	0.2418	-0.07553	0.04309	-0.1316
cg19588649	0.1251	-0.05078	0.01515	-0.1242	0.09553	-0.0587
cg19590050	0.0611	-0.06435	0.02913	-0.1036	0.2936	-0.02605
cg19591696	0.03514	-0.06812	0.01351	-0.1138	0.04152	-0.07874
cg19620629	0.008231	-0.1017	0.125	-0.04677	0.03447	-0.06671
cg19643545	0.03657	-0.07457	0.2803	-0.04056	0.02231	-0.1049
cg19644428	0.02848	0.111	0.3276	0.03628	0.03053	0.08826
cg19645258	0.03716	0.09731	0.5913	0.028	0.01612	0.1112
cg19652717	0.05954	0.09822	0.1523	0.07976	0.03223	0.1254
cg19665213	0.2671	0.05642	0.8991	-0.01127	0.03459	0.1309
cg19669193	0.01435	0.1144	0.2358	-0.04935	0.02099	0.09878
cg19676686	0.3164	-0.05324	0.03852	-0.1295	0.4847	-0.03518
cg19687473	0.06403	-0.08534	0.04232	-0.107	0.3358	-0.04341
cg19705320	0.3493	-0.03917	0.04635	-0.1079	0.04067	-0.08772
cg19716950	0.07604	-0.06917	0.939	0.003286	0.0163	-0.1032
cg19742736	0.963	-0.001958	0.04721	-0.1152	0.1706	-0.0621
cg19792683	0.01643	0.1116	0.8699	0.006016	0.06406	0.06018
cg19810954	0.0147	0.1164	0.1569	0.07235	0.01175	0.1198
cg19815589	0.01215	-0.1218	0.1028	-0.05352	0.0858	-0.07682
cg19816250	0.1235	-0.071	0.0412	-0.1215	0.04343	-0.1195
cg19827716	0.0665	0.1223	0.05332	0.223	0.03546	0.1638
cg19835482	0.04316	0.09537	0.1784	0.0712	0.02235	0.104
cg19851526	0.0799	-0.0737	0.1029	-0.0725	0.02007	-0.1076
cg19860548	0.03963	0.1136	0.3572	0.04634	0.0335	0.1186
cg19869734	0.03517	-0.0695	0.03013	-0.1063	0.03941	-0.06519
cg19886981	0.01358	0.1043	0.5674	0.02143	0.01787	0.0979
cg19915449	0.008679	-0.06989	0.01358	-0.1118	0.02487	-0.05247
cg19918774	0.3712	-0.02777	0.04145	-0.1112	0.503	-0.0251
cg19926245	0.04388	-0.07237	0.01596	-0.1107	0.2554	-0.03115
cg19927586	0.9284	-0.003557	0.03241	-0.106	0.6132	-0.01938
cg19931583	0.0861	-0.1002	0.817	-0.01458	0.03937	-0.1158
cg19940625	0.1903	-0.04792	0.7457	0.01703	0.02332	-0.1085
cg19981301	0.4263	-0.01926	0.01366	-0.141	0.3578	-0.02351

cg20007394	0.5953	0.01496	0.02071	-0.109	0.3453	-0.02531
cg20011788	0.02988	-0.1038	0.04678	-0.09787	0.3613	-0.04353
cg20025677	0.03035	0.08786	0.2179	0.05195	0.01973	0.1084
cg20031394	0.04149	-0.1032	0.8744	-0.008058	0.09259	-0.07263
cg20046896	0.07901	0.06795	0.02134	0.1317	0.0218	0.08682
cg20061296	0.9073	0.008941	0.04998	-0.1557	0.5137	-0.03439
cg20102045	0.4353	-0.02841	0.03343	-0.1084	0.03891	-0.08736
cg20127596	0.5474	-0.01926	0.0411	-0.1075	0.2026	-0.0383
cg20141509	0.4495	-0.02101	0.0282	-0.1178	0.01931	-0.08451
cg20154455	0.09285	-0.05825	0.1868	-0.04216	0.01597	-0.1054
cg20202792	0.0196	0.1044	0.08144	0.07999	0.06825	0.07452
cg20203365	0.2658	-0.03801	0.01513	-0.129	0.07779	-0.07346
cg20207715	0.007999	-0.08717	0.2052	-0.03217	0.006903	-0.1041
cg20238164	0.02562	-0.07366	0.03479	-0.1054	0.00852	-0.1079
cg20287512	0.01203	0.106	0.8756	0.007212	0.9799	0.0008599
cg20290167	0.03755	-0.1002	0.8032	-0.01103	0.03207	-0.09378
cg20316986	0.02965	-0.1351	0.05555	-0.1062	0.232	-0.0548
cg20335039	0.9506	0.001961	0.0336	-0.1046	0.6699	-0.01576
cg20379345	0.02545	-0.1024	0.7281	0.01545	0.07093	-0.05586
cg20379470	0.2555	0.03286	0.03439	-0.1073	0.09988	-0.03705
cg20391567	0.06244	0.1906	0.101	0.2259	0.0496	0.2305
cg20411395	0.009879	-0.1229	0.1235	-0.06627	0.03521	-0.07341
cg20412744	0.0377	-0.124	0.2232	-0.05069	0.3511	-0.03901
cg20424182	0.7421	-0.009105	0.0159	-0.1078	0.09198	-0.05424
cg20463995	0.118	-0.05718	0.03019	-0.1193	0.05009	-0.07971
cg20466064	0.01821	-0.1061	0.2032	-0.04867	0.1193	-0.05767
cg20473723	0.03402	0.1475	0.2369	0.07526	0.04248	0.1319
cg20503288	0.02348	-0.1018	0.5541	-0.01197	0.05392	-0.07951
cg20521198	0.02354	-0.3245	0.9523	0.01281	0.07218	-0.2112
cg20523525	0.5132	-0.01434	0.01827	-0.1245	0.1779	-0.03419
cg20530876	0.01883	-0.1502	0.1573	-0.09199	0.2462	-0.05292
cg20548859	0.02591	0.1138	0.2337	0.05089	0.03873	0.1032
cg20572967	0.07528	-0.04309	0.01746	-0.1023	0.1403	-0.03878
cg20598768	0.03795	0.1923	0.449	-0.03523	0.9304	0.006621
cg20645912	0.2387	0.08215	0.8275	-0.01939	0.02334	0.1749
cg20661257	0.03003	-0.1496	0.07597	-0.09418	0.03494	-0.1302
cg20672131	0.01673	0.1403	0.3884	0.04199	0.0231	0.134
cg20707512	0.2509	-0.03037	0.01616	-0.1209	0.2387	-0.03892
cg20784899	0.01478	-0.1267	0.1195	-0.05245	0.01296	-0.09932
cg20795083	0.871	-0.005971	0.01746	-0.1001	0.04197	-0.06981
cg20829834	0.02484	0.08931	0.6315	0.02076	0.01178	0.1056
cg20884362	0.2106	-0.03696	0.01954	-0.1047	0.3613	-0.02643
cg20887670	0.1012	-0.07867	0.03615	-0.1525	0.01522	-0.1255
cg20891116	0.01874	-0.1008	0.2762	-0.02604	0.03195	-0.06272
cg20899927	0.01926	0.123	0.04336	0.1012	0.05239	0.08332
cg20938830	0.01236	-0.1013	0.2018	-0.03929	0.03523	-0.1004
cg20948271	0.7312	-0.009412	0.02269	-0.1079	0.947	-0.00262
cg20963142	0.1505	-0.06404	0.574	-0.03624	0.02628	-0.1375
cg20970245	0.0141	0.1152	0.8307	0.009635	0.01346	0.1076
cg20984688	0.06862	-0.07566	0.9005	-0.005765	0.03224	-0.1002

cg21004104	0.01879	0.1112	0.703	-0.0131	0.02224	0.0722
cg21069434	0.7228	-0.01207	0.03032	-0.06616	0.006986	-0.1202
cg21092526	0.01061	-0.0998	0.02362	-0.1042	0.02529	-0.08186
cg21127508	0.04485	-0.07833	0.1899	-0.05254	0.0273	-0.1002
cg21158587	0.01664	-0.1032	0.8068	-0.007752	0.1357	-0.04519
cg21162961	0.3646	-0.03802	0.04019	-0.1245	0.9157	-0.006792
cg21197023	0.04109	0.1011	0.9465	0.005042	0.09364	0.08266
cg21203643	0.05246	-0.09053	0.792	-0.0146	0.03019	-0.1135
cg21237749	0.1785	-0.05495	0.7727	0.01117	0.0238	-0.1041
cg21240332	0.02752	-0.0715	0.006942	-0.1101	0.3297	-0.02367
cg21240580	0.3919	-0.0273	0.0474	-0.1001	0.1139	-0.04667
cg21305756	0.03544	-0.1107	0.7068	-0.02867	0.3556	-0.053
cg21318267	0.008927	0.1079	0.1125	0.05222	0.01847	0.08272
cg21351733	0.04047	0.0792	0.8013	0.006424	0.008895	0.105
cg21364330	0.01517	-0.1136	0.05263	-0.0826	0.04519	-0.08833
cg21365444	0.1497	-0.04857	0.02106	-0.1005	0.1527	-0.03683
cg21392047	0.1679	-0.04606	0.0402	-0.1017	0.6115	-0.02167
cg21402968	0.03389	-0.1017	0.8423	-0.00802	0.0665	-0.081
cg21406461	0.03838	0.1184	0.9202	0.007614	0.05383	0.1098
cg21410051	0.01455	-0.1127	0.2612	-0.03282	0.04149	-0.08317
cg21416120	0.2143	-0.03877	0.0345	-0.1024	0.05731	-0.06556
cg21426441	0.04358	-0.1042	0.8623	-0.01174	0.06846	-0.08936
cg21449673	0.04764	-0.1235	0.7412	0.0179	0.06257	-0.1073
cg21453209	0.03569	-0.1053	0.546	-0.03472	0.04504	-0.09529
cg21457147	0.1752	-0.06187	0.4837	0.04567	0.03357	-0.1087
cg21461856	0.0852	0.08469	0.8793	0.009455	0.03208	0.1127
cg21535931	0.1055	0.05092	0.02711	0.1014	0.4984	0.02128
cg21543897	0.04895	-0.08093	0.01596	-0.129	0.02685	-0.1065
cg21545073	0.7051	-0.01285	0.03357	-0.1079	0.7642	-0.01019
cg21548109	0.08882	-0.06581	0.1095	-0.06972	0.02536	-0.1037
cg21549666	0.04393	0.2823	0.8957	0.02203	0.1108	0.1956
cg21550098	0.156	-0.06266	0.705	-0.0161	0.03921	-0.1103
cg21582489	0.02834	0.1021	0.2533	0.05135	0.05175	0.08614
cg21585996	0.04111	0.1071	0.8985	-0.007929	0.8454	0.009821
cg21586613	0.06865	-0.06586	0.04192	-0.1032	0.1453	-0.05345
cg21588792	0.01077	-0.1083	0.9146	0.003419	0.01066	-0.1101
cg21601056	0.5663	-0.02256	0.02961	-0.1082	0.2021	-0.03688
cg21619224	0.005874	-0.08539	0.01746	-0.06619	0.006853	-0.1044
cg21664764	0.0217	0.1027	0.2816	0.03243	0.05779	0.05929
cg21707633	0.398	-0.02062	0.02264	-0.1036	0.02467	-0.05962
cg21742058	0.04605	-0.1024	0.8663	0.007334	0.1225	-0.06381
cg21769057	0.02812	-0.07384	0.1692	-0.06266	0.02864	-0.1047
cg21774861	0.21	-0.03836	0.01734	-0.1234	0.5478	-0.01816
cg21788470	0.03177	-0.1011	0.9874	0.0005444	0.1115	-0.04011
cg21805179	0.1158	-0.08464	0.1699	-0.06544	0.04127	-0.1029
cg21814995	0.1295	-0.06878	0.09069	-0.08838	0.01581	-0.1181
cg21895182	0.6864	-0.01745	0.04053	-0.1348	0.1524	-0.07415
cg21911007	0.0328	0.1264	0.4755	0.04357	0.0634	0.08499
cg21916600	0.08155	0.09541	0.992	0.0008102	0.03852	0.103
cg21921829	0.03568	0.1233	0.3682	0.06729	0.0278	0.1316

cg21931078	0.5288	-0.01783	0.04524	-0.1026	0.1083	-0.07096
cg21975400	0.03266	0.1337	0.3203	0.05792	0.05177	0.1177
cg21977507	0.02891	-0.1223	0.3355	-0.04971	0.04502	-0.1006
cg21980820	0.3751	0.04691	0.04269	-0.1374	0.1856	0.08614
cg22009470	0.1406	0.07256	0.04053	0.119	0.1434	0.07624
cg22011560	0.2009	-0.09134	0.04867	-0.1678	0.501	-0.04667
cg22030962	0.01639	-0.1026	0.3775	-0.02745	0.03054	-0.06446
cg22036069	0.02053	-0.1103	0.05972	-0.1008	0.008859	-0.1523
cg22061809	0.02472	-0.1275	0.343	-0.03842	0.01684	-0.118
cg22115168	0.5053	-0.0162	0.037	-0.1044	0.9761	-0.0008689
cg22117381	0.204	-0.05298	0.6102	-0.02261	0.04597	-0.1206
cg22128067	0.03532	0.1253	0.5352	-0.03123	0.05392	0.1071
cg22128896	0.01044	0.1789	0.7307	-0.01488	0.8865	0.01936
cg22142922	0.02351	-0.1038	0.943	-0.003336	0.03485	-0.09683
cg22157340	0.5854	0.02274	0.04374	-0.08037	0.03488	0.1006
cg22158639	0.02598	-0.0694	0.3223	-0.02814	0.005844	-0.1049
cg22185875	0.1993	-0.06195	0.04183	-0.1423	0.5932	-0.02749
cg22215784	0.02979	0.1229	0.0714	0.1091	0.1265	0.07107
cg22220674	0.5634	0.027	0.04949	-0.1135	0.6957	-0.01967
cg22221487	0.07473	0.06961	0.9211	0.005396	0.02317	0.1116
cg22226592	0.04087	0.0887	0.3425	0.03987	0.02771	0.1032
cg22230947	0.0386	-0.1039	0.8107	-0.01263	0.165	-0.05191
cg22234154	0.3059	-0.04269	0.3187	-0.05267	0.04082	-0.1069
cg22250390	0.7208	-0.009845	0.0355	-0.1046	0.8289	-0.006811
cg22256960	0.02582	-0.1362	0.2813	-0.07384	0.02255	-0.1311
cg22257236	0.1867	-0.1051	0.04487	-0.1755	0.1452	-0.1153
cg22292025	0.07162	-0.07317	0.0421	-0.1102	0.1684	-0.05287
cg22301270	0.1171	-0.06172	0.4223	-0.03715	0.01548	-0.1092
cg22308937	0.024	-0.139	0.8395	0.01225	0.4692	-0.0415
cg22309080	0.43	0.02084	0.03182	-0.1047	0.06564	-0.0599
cg22312738	0.0969	-0.06576	0.02796	-0.1126	0.2891	-0.04232
cg22325858	0.2266	0.0411	0.02146	-0.1056	0.9639	-0.001753
cg22335635	0.2143	-0.03857	0.02362	-0.1048	0.1315	-0.05475
cg22354468	0.03098	0.1345	0.1559	0.06893	0.1104	0.05974
cg22355498	0.4336	-0.03274	0.8536	-0.01043	0.04806	-0.1069
cg22407629	0.04484	0.1193	0.7342	0.01987	0.3393	0.04661
cg22455642	0.5634	-0.02334	0.04406	-0.1115	0.9042	0.003623
cg22475834	0.2314	-0.0414	0.04005	-0.1156	0.09816	-0.06478
cg22494263	0.04583	0.1097	0.293	0.08039	0.05675	0.1192
cg22502776	0.4548	-0.03613	0.03624	-0.1177	0.9267	0.004822
cg22534690	0.04839	-0.07578	0.7519	-0.01465	0.01339	-0.1016
cg22543173	0.6058	-0.015	0.02779	-0.107	0.3942	-0.03168
cg22553273	0.04143	-0.126	0.4165	-0.04767	0.01499	-0.1551
cg22609511	0.1056	0.09192	0.4015	0.04644	0.03053	0.1617
cg22613078	0.3106	0.03659	0.1291	-0.07152	0.03169	0.1054
cg22627734	0.5487	-0.02628	0.02693	-0.1154	0.4056	-0.0389
cg22627750	0.02824	0.1234	0.08426	0.08171	0.06314	0.08235
cg22658654	0.7175	0.01064	0.03234	-0.1037	0.9995	-2.50E-05
cg22671903	0.02708	0.07832	0.2812	-0.03247	0.006919	0.1023
cg22681777	0.03678	0.1064	0.5623	0.03529	0.04477	0.0904

cg22699422	0.1407	-0.06817	0.03205	-0.1447	0.03435	-0.09813
cg22700015	0.06635	-0.08423	0.04667	-0.09938	0.03471	-0.1061
cg22703515	0.484	0.02424	0.02155	-0.117	0.2005	-0.04485
cg22706992	0.04272	-0.1107	0.9876	0.001007	0.2565	-0.0654
cg22718891	0.03154	0.1015	0.9276	0.003791	0.0575	0.07613
cg22719250	0.08533	-0.05289	0.03018	-0.101	0.05209	-0.07117
cg22726220	0.6786	-0.01672	0.02711	-0.1434	0.3682	-0.03263
cg22741129	0.06721	0.07538	0.6438	0.02335	0.01741	0.1174
cg22798521	0.1392	-0.04669	0.04406	-0.1105	0.1124	-0.05667
cg22803262	0.03438	0.1048	0.8407	-0.01074	0.05147	0.08653
cg22814944	0.08494	-0.06576	0.043	-0.1004	0.188	-0.05824
cg22826902	0.01988	0.1122	0.848	-0.008406	0.02993	0.1066
cg22835215	0.05013	-0.09891	0.6962	0.01483	0.01778	-0.1125
cg22850860	0.1163	-0.03989	0.04701	-0.0686	0.01339	-0.1051
cg22851864	0.06958	-0.09209	0.9155	-0.008141	0.03559	-0.1166
cg22852858	0.02737	-0.11	0.8279	-0.01144	0.07104	-0.07472
cg22858170	0.8963	0.002967	0.03262	-0.1038	0.7047	-0.01099
cg22888311	0.03311	0.08481	0.9412	0.004135	0.01552	0.1143
cg22911931	0.204	-0.0334	0.1086	-0.0653	0.02052	-0.1133
cg22917617	0.1239	-0.07972	0.7357	0.01989	0.0493	-0.1021
cg22938469	0.03107	-0.1046	0.1925	-0.04828	0.2083	-0.05329
cg22939703	0.01879	-0.1132	0.1761	-0.03952	0.1657	-0.06002
cg22965487	0.02101	-0.1167	0.747	0.01326	0.06984	-0.07688
cg22985036	0.04136	0.1166	0.1814	0.09025	0.02785	0.1423
cg22986091	0.2381	-0.03914	0.03449	-0.1199	0.08871	-0.07504
cg22994019	0.01044	-0.1101	0.08192	-0.06363	0.02964	-0.0782
cg22998812	0.02225	-0.1139	0.4048	-0.02437	0.04577	-0.06248
cg23013736	0.4203	0.01523	0.0266	-0.1092	0.3052	0.02189
cg23014794	0.009717	-0.1256	0.04711	-0.1082	0.04074	-0.08221
cg23023953	0.8519	-0.003788	0.01339	-0.1043	0.369	-0.02061
cg23024047	0.03099	-0.08063	0.2775	-0.03884	0.01979	-0.1021
cg23028728	0.7869	0.009641	0.5835	-0.03839	0.03942	-0.1027
cg23029289	0.02119	-0.1021	0.1121	-0.05752	0.08956	-0.05949
cg23039425	0.1351	0.07512	0.07261	0.1085	0.02868	0.1303
cg23043514	0.02199	-0.1003	0.08597	-0.07204	0.04036	-0.08861
cg23050025	0.4024	-0.02873	0.03619	-0.1013	0.9323	-0.003727
cg23079522	0.5486	0.01652	0.03422	-0.1087	0.7735	0.01028
cg23087770	0.06345	-0.1137	0.8415	-0.01599	0.03781	-0.1434
cg23088600	0.6907	-0.01796	0.261	-0.06968	0.04805	-0.1082
cg23102129	0.5029	-0.01383	0.01339	-0.104	0.6895	-0.01084
cg23135286	0.03166	0.1179	0.1728	0.07344	0.01386	0.1474
cg23139797	0.02662	-0.1084	0.8587	-0.006002	0.07528	-0.08698
cg23193363	0.05658	-0.0986	0.8103	-0.01297	0.03719	-0.1185
cg23202321	0.1047	0.09135	0.9396	0.006005	0.04241	0.1357
cg23206194	0.03833	-0.1103	0.3631	-0.04078	0.01983	-0.1216
cg23233952	0.005874	-0.1031	0.08556	-0.03996	0.01292	-0.06171
cg23238327	0.02548	-0.1259	0.9371	0.005798	0.1295	-0.06139
cg23261107	0.09117	0.07981	0.8159	-0.01053	0.02197	0.1008
cg23262213	0.03937	0.1014	0.635	0.02341	0.07456	0.07916
cg23278604	0.02932	0.1154	0.4219	0.03841	0.6667	-0.01511

cg23285202	0.0233	-0.1012	0.7031	0.01795	0.2543	-0.04991
cg23314948	0.2218	-0.0482	0.1517	-0.06816	0.03694	-0.1022
cg23323906	0.02089	0.101	0.9065	-0.007899	0.1028	0.05697
cg23325502	0.1381	-0.04636	0.2199	-0.03805	0.008244	-0.1026
cg23342296	0.03612	-0.134	0.4364	-0.03264	0.0192	-0.1052
cg23358787	0.5313	-0.02795	0.03013	-0.1579	0.6235	-0.02422
cg23362669	0.04912	0.1106	0.915	0.006045	0.0456	0.1001
cg23363481	0.4548	-0.02929	0.03233	-0.1216	0.1634	-0.05844
cg23382549	0.01497	-0.1438	0.1306	-0.09562	0.02532	-0.144
cg23394510	0.09941	-0.0509	0.04932	-0.06376	0.01009	-0.1226
cg23413497	0.04056	0.1029	0.8623	0.01191	0.9999	-6.64E-06
cg23462338	0.04716	-0.1146	0.9297	0.005308	0.1189	-0.06742
cg23472335	0.1039	0.08041	0.8721	-0.01087	0.03283	0.1163
cg23476312	0.04021	-0.09455	0.01672	-0.1311	0.03198	-0.1042
cg23497707	0.9749	0.001219	0.04811	-0.1084	0.3375	-0.02791
cg23530239	0.02965	0.1442	0.6701	0.02444	0.015	0.1853
cg23531585	0.08508	-0.07359	0.0411	-0.1101	0.04092	-0.1015
cg23586018	0.03694	0.1468	0.9959	0.0004982	0.04967	0.1125
cg23599947	0.4093	-0.03434	0.01839	-0.1427	0.06101	-0.07913
cg23603057	0.0108	0.1076	0.5566	-0.01369	0.05747	0.05411
cg23630124	0.4408	-0.01307	0.02024	-0.1121	0.1071	-0.056
cg23631465	0.09017	-0.06689	0.3708	-0.04253	0.02235	-0.1336
cg23633028	0.2037	-0.04626	0.1089	-0.07405	0.04522	-0.1115
cg23651157	0.02461	0.1093	0.06573	-0.0727	0.1529	0.07011
cg23666588	0.04567	-0.1037	0.4852	-0.03995	0.05572	-0.09969
cg23683779	0.04209	-0.1334	0.7888	-0.01744	0.09546	-0.1028
cg23689304	0.03839	-0.1069	0.9848	0.001123	0.1168	-0.06419
cg23700062	0.04535	-0.07104	0.04888	-0.08571	0.02361	-0.1084
cg23717600	0.005874	-0.1013	0.1277	-0.0356	0.02951	-0.07211
cg23728761	0.0375	0.1342	0.2646	0.07135	0.03697	0.1274
cg23741855	0.01401	0.1077	0.4921	-0.03214	0.04465	0.08791
cg23755033	0.6488	0.01323	0.02531	-0.1002	0.8417	-0.007511
cg23797052	0.1875	-0.05698	0.0409	-0.1319	0.03752	-0.1189
cg23803120	0.5079	-0.01749	0.04312	-0.1127	0.1182	-0.04221
cg23824502	0.5811	-0.02077	0.06024	-0.09559	0.03092	-0.1009
cg23830281	0.02064	0.1385	0.2852	0.03566	0.2111	0.03183
cg23840821	0.01568	0.1064	0.05851	0.06589	0.3975	0.02493
cg23849677	0.02586	0.1241	0.8895	0.01004	0.7563	0.01078
cg23867647	0.5458	-0.02794	0.02305	-0.116	0.006846	-0.1397
cg23871920	0.9427	-0.003457	0.02501	-0.1085	0.06017	-0.07917
cg23887878	0.03621	-0.1082	0.02132	-0.1623	0.05319	-0.1017
cg23889476	0.1004	0.07995	0.8466	0.01386	0.0141	0.1281
cg23906786	0.9415	-0.002092	0.03186	-0.1142	0.9425	-0.00206
cg23920494	0.1512	-0.06531	0.2926	-0.05704	0.01981	-0.1053
cg23934000	0.5348	-0.02466	0.04752	-0.139	0.8836	-0.008707
cg23975251	0.437	-0.02371	0.01351	-0.1004	0.7062	-0.01218
cg23986315	0.124	-0.0837	0.03169	-0.1497	0.3108	-0.03762
cg24006939	0.02131	0.1081	0.6828	0.01753	0.02118	0.09966
cg24019478	0.2404	-0.04798	0.2115	-0.06274	0.0141	-0.1015
cg24029640	0.021	0.1187	0.8165	-0.009659	0.06586	0.06418

cg24062313	0.04724	-0.1437	0.8629	-0.01206	0.1581	-0.08657
cg24070446	0.03975	0.1081	0.5473	0.0263	0.05734	0.1005
cg24101638	0.0144	-0.0874	0.03158	-0.09832	0.008447	-0.1112
cg24105648	0.02292	-0.09129	0.01122	-0.131	0.04136	-0.09688
cg24109894	0.7928	-0.01086	0.02796	-0.1485	0.5153	-0.02889
cg24116052	0.02813	-0.1032	0.7368	0.01482	0.07946	-0.0721
cg24139233	0.04688	-0.1253	0.8594	-0.01078	0.2868	-0.04662
cg24152283	0.3515	-0.04987	0.04195	-0.1377	0.2657	-0.05449
cg24169735	0.1417	0.08022	0.8624	0.0166	0.02326	0.126
cg24215868	0.1176	-0.06533	0.9858	0.001438	0.01727	-0.1115
cg24219589	0.9187	0.00392	0.01906	-0.13	0.9895	0.0006024
cg24221357	0.1723	-0.06806	0.2054	0.05901	0.04124	-0.1168
cg24239590	0.02797	0.1027	0.4471	0.03415	0.1211	0.06508
cg24239922	0.01943	-0.1225	0.04206	-0.1334	0.2678	-0.04049
cg24245297	0.02208	0.1107	0.5224	-0.01622	0.03153	0.111
cg24260323	0.01587	-0.101	0.1387	-0.06756	0.3054	-0.04036
cg24272305	0.01933	-0.1821	0.7404	0.03256	0.2056	-0.08218
cg24276053	0.01833	-0.1251	0.02907	-0.1436	0.06327	-0.1344
cg24285017	0.1453	-0.05443	0.9787	-0.001461	0.04322	-0.1132
cg24310667	0.03452	0.1627	0.1599	0.09432	0.0478	0.1385
cg24334833	0.07569	-0.0658	0.08629	-0.05328	0.0127	-0.1065
cg24341059	0.5251	-0.01475	0.01465	-0.1434	0.4722	-0.01816
cg24345184	0.03046	-0.1187	0.7637	-0.01583	0.05008	-0.1109
cg24353819	0.8947	0.007593	0.04581	-0.1517	0.9682	-0.002535
cg24372701	0.01677	-0.1331	0.2174	0.03354	0.4186	-0.03859
cg24373618	0.04402	0.131	0.5281	0.04013	0.04372	0.1328
cg24425178	0.04232	-0.1118	0.7589	-0.01743	0.02307	-0.109
cg24454759	0.03351	-0.1076	0.7952	-0.01327	0.03853	-0.08773
cg24465960	0.07562	-0.06992	0.536	-0.02132	0.00852	-0.1245
cg24478001	0.02366	0.1293	0.7741	-0.008144	0.8585	0.006962
cg24505819	0.1379	-0.0738	0.03646	-0.1236	0.2106	-0.05929
cg24530218	0.03508	-0.1226	0.1628	0.05217	0.1987	-0.083
cg24550172	0.4599	0.01966	0.02918	-0.1016	0.6167	0.01684
cg24550880	0.02652	-0.1096	0.6714	-0.01936	0.05528	-0.08561
cg24569985	0.07482	-0.1145	0.2862	-0.07313	0.03797	-0.1599
cg24583282	0.1669	-0.04798	0.02076	-0.1046	0.1827	-0.04099
cg24601055	0.06922	-0.093	0.9553	0.003364	0.03599	-0.1045
cg24605576	0.04372	-0.104	0.7143	-0.02046	0.1166	-0.05977
cg24650734	0.1035	0.08231	0.5014	0.04028	0.04869	0.1034
cg24657022	0.0587	-0.07642	0.04183	-0.07677	0.01717	-0.1133
cg24666046	0.04691	0.1005	0.9401	0.004235	0.1791	0.06058
cg24706802	0.1193	-0.07324	0.04362	-0.1159	0.2192	-0.0534
cg24721399	0.4498	-0.018	0.03546	-0.1103	0.08747	-0.04149
cg24736099	0.01149	-0.1149	0.4155	-0.0288	0.6437	0.01072
cg24771111	0.07043	-0.0461	0.05559	-0.06473	0.008859	-0.106
cg24828322	0.3226	-0.03658	0.0474	-0.1018	0.5058	-0.02965
cg24835244	0.03364	-0.09413	0.05475	-0.07403	0.006853	-0.1051
cg24850121	0.04198	-0.1392	0.8216	0.01705	0.05974	-0.1179
cg24869879	0.04083	-0.1208	0.2577	-0.06838	0.06081	-0.1084
cg24871445	0.04257	-0.1102	0.7342	0.01748	0.03802	-0.1026

cg24915409	0.005874	0.08726	0.1197	-0.03331	0.004085	0.1136
cg24945863	0.557	-0.02556	0.3318	-0.057	0.04638	-0.1035
cg24959045	0.7077	-0.01091	0.03309	-0.1126	0.1594	-0.04503
cg24982343	0.01906	-0.1037	0.3589	0.03818	0.8342	0.02121
cg24990564	0.04297	-0.1215	0.08558	-0.09637	0.1343	-0.07333
cg25001923	0.007999	-0.09145	0.1926	-0.03993	0.00649	-0.1233
cg25020666	0.3308	0.04117	0.8155	-0.01135	0.00852	0.1142
cg25035320	0.02617	0.1051	0.8123	0.01315	0.03878	0.09053
cg25052941	0.08486	-0.09462	0.9259	0.005805	0.03415	-0.1227
cg25084175	0.2557	-0.04286	0.04721	-0.1096	0.08739	-0.07204
cg25091323	0.04247	-0.1121	0.03005	-0.1678	0.03487	-0.1268
cg25106607	0.1835	-0.04807	0.03558	-0.1008	0.07899	-0.06481
cg25212717	0.6922	0.01493	0.04183	-0.1027	0.4625	-0.02719
cg25214898	0.03167	-0.09201	0.386	-0.03231	0.01014	-0.1212
cg25226068	0.02142	-0.1026	0.7393	0.01401	0.08962	-0.07002
cg25246070	0.04129	0.1016	0.4074	-0.03618	0.09885	0.07115
cg25273027	0.01471	-0.08955	0.02322	-0.06932	0.004664	-0.1011
cg25322106	0.01662	-0.1184	0.5207	-0.01441	0.06662	-0.04744
cg25325873	0.2701	-0.03236	0.04989	-0.08157	0.01296	-0.1198
cg25330875	0.6694	0.01961	0.0263	-0.1168	0.3539	0.04256
cg25342674	0.02749	-0.1186	0.01584	-0.125	0.0488	-0.1081
cg25351578	0.1706	-0.04338	0.07903	-0.0703	0.01204	-0.1224
cg25409529	0.0377	-0.09237	0.03725	-0.1035	0.02391	-0.09956
cg25430793	0.01682	0.1599	0.4645	-0.04348	0.5707	0.02472
cg25431432	0.2328	-0.03489	0.01579	-0.1079	0.2221	-0.04778
cg25442687	0.05683	-0.09457	0.6015	-0.03165	0.04784	-0.1046
cg25445341	0.03238	-0.1036	0.8419	-0.01063	0.1791	-0.06907
cg25446153	0.09703	0.075	0.3326	-0.05277	0.03459	0.1032
cg25481384	0.02142	-0.1186	0.6886	-0.01497	0.06674	-0.08618
cg25490194	0.3406	-0.03208	0.02029	-0.1132	0.6429	-0.01766
cg25497545	0.01569	0.1412	0.8797	-0.008124	0.1959	0.05464
cg25581843	0.4822	-0.02522	0.02509	-0.134	0.5687	-0.02335
cg25636182	0.141	-0.05729	0.02763	-0.1271	0.09973	-0.05597
cg25638714	0.1412	-0.05661	0.0333	-0.1101	0.01222	-0.1113
cg25662254	0.168	0.02914	0.04366	-0.1114	0.3883	0.01762
cg25669783	0.7859	0.01186	0.03173	-0.1297	0.4509	-0.03057
cg25673596	0.1437	-0.06364	0.4589	-0.0418	0.02603	-0.1269
cg25681958	0.1966	-0.03711	0.02605	-0.1116	0.1254	-0.04653
cg25768573	0.02232	-0.1435	0.04334	-0.1361	0.04318	-0.1586
cg25769122	0.00654	-0.1051	0.391	-0.02693	0.009027	-0.09419
cg25782229	0.04044	0.09625	0.6833	0.0146	0.01599	0.1115
cg25801807	0.3417	0.02987	0.03436	-0.1072	0.3318	-0.03003
cg25823314	0.04352	-0.05809	0.985	-0.0005888	0.02357	-0.102
cg25830719	0.6251	0.01877	0.0474	-0.1178	0.5465	-0.02336
cg25856220	0.01481	-0.09694	0.131	-0.04683	0.01065	-0.1058
cg25862826	0.8765	0.006752	0.02998	-0.118	0.3107	-0.04148
cg25863892	0.4697	0.02636	0.04013	-0.1204	0.6872	0.01874
cg25921756	0.1205	0.1047	0.6044	0.03839	0.04615	0.1216
cg25923214	0.02673	0.1978	0.04728	0.1576	0.139	0.1065
cg25943727	0.02338	-0.1351	0.2189	-0.06349	0.01578	-0.163

cg25955330	0.03862	0.1458	0.6517	0.03335	0.1194	0.0972
cg25959317	0.2178	-0.0525	0.4756	-0.03454	0.02224	-0.1182
cg25972537	0.02616	0.1022	0.1808	0.06214	0.0741	0.07586
cg25973563	0.05767	-0.0853	0.7625	0.01863	0.04828	-0.1083
cg25999594	0.01625	-0.1029	0.06722	-0.04135	0.009027	-0.07394
cg26011615	0.1849	-0.09458	0.6943	0.03735	0.04675	-0.1432
cg26027478	0.03359	0.1018	0.4159	0.04048	0.07364	0.09614
cg26030553	0.03795	-0.1084	0.8351	0.009403	0.09807	-0.0648
cg26068079	0.0753	0.08892	0.6164	0.02042	0.0163	0.1195
cg26079423	0.5769	-0.03085	0.0474	-0.1363	0.4946	-0.04322
cg26161661	0.01988	-0.08041	0.111	-0.05772	0.01057	-0.1048
cg26168383	0.05147	-0.05509	0.01959	-0.1036	0.2558	-0.02806
cg26188658	0.04681	-0.1391	0.2112	-0.07399	0.08992	-0.1058
cg26206686	0.03849	-0.1601	0.5466	-0.05422	0.3814	-0.056
cg26240235	0.0254	0.06112	0.6009	0.01482	0.01467	0.1015
cg26241591	0.4633	-0.02341	0.02221	-0.1252	0.8888	0.005317
cg26271001	0.3619	-0.02465	0.01966	-0.1084	0.3788	-0.02008
cg26281107	0.03365	0.1028	0.6717	-0.01937	0.04688	0.1094
cg26288438	0.03104	0.1836	0.985	-0.0008831	0.529	0.03468
cg26364693	0.04272	-0.1005	0.3695	-0.04536	0.3629	-0.02582
cg26369576	0.04979	-0.126	0.8913	-0.00856	0.06375	-0.1007
cg26373662	0.284	0.0505	0.5515	-0.03805	0.02188	0.108
cg26374058	0.1172	-0.05687	0.1606	-0.02752	0.01717	-0.1148
cg26382607	0.02622	-0.1169	0.6425	0.02161	0.0699	-0.0802
cg26389958	0.9495	0.002551	0.02619	-0.1121	0.922	-0.002964
cg26434332	0.0134	0.141	0.2686	0.06386	0.01918	0.1251
cg26439474	0.04335	-0.08555	0.02022	-0.1077	0.1948	-0.05408
cg26495357	0.04198	0.1024	0.5963	-0.02163	0.01768	0.1362
cg26498966	0.09064	0.08478	0.7614	-0.01759	0.03653	0.1037
cg26528940	0.1164	-0.0616	0.0474	-0.1192	0.5598	-0.02746
cg26551334	0.03372	-0.09319	0.03818	-0.08323	0.02048	-0.1097
cg26567688	0.5813	-0.01874	0.01339	-0.1309	0.997	-0.0001867
cg26620978	0.01524	0.06934	0.8987	-0.004394	0.00451	0.1068
cg26643317	0.02578	-0.1349	0.335	-0.04442	0.04498	-0.09735
cg26650885	0.01958	0.07241	0.3623	0.0277	0.01918	0.1024
cg26667773	0.01401	0.1166	0.3212	0.0352	0.1007	0.07826
cg26672652	0.3997	-0.03312	0.0216	-0.1281	0.1005	-0.0628
cg26685255	0.6156	0.01675	0.02796	-0.1481	0.9861	0.0008532
cg26689716	0.0284	-0.1018	0.5175	-0.02728	0.04211	-0.08469
cg26689777	0.2313	-0.05966	0.9752	-0.002234	0.02897	-0.1033
cg26702908	0.01344	-0.1476	0.2023	-0.04554	0.004976	-0.1645
cg26708970	0.7018	0.01836	0.04867	-0.1174	0.6465	0.02142
cg26717322	0.01044	-0.1017	0.2713	-0.02856	0.0161	-0.08569
cg26783079	0.01784	-0.08444	0.7009	-0.01314	0.006846	-0.1273
cg26785468	0.9863	-0.001042	0.9455	0.005261	0.02784	-0.1382
cg26800107	0.7471	0.01427	0.04597	-0.1379	0.1518	0.047
cg26814100	0.01747	-0.1806	0.4024	-0.04329	0.2615	-0.05342
cg26823617	0.1633	-0.05215	0.4625	-0.03512	0.04999	-0.1129
cg26840333	0.08427	0.08579	0.9263	-0.006869	0.04554	0.1103
cg26848331	0.05476	-0.09904	0.9242	-0.005053	0.02772	-0.1166

cg26849111	0.2043	-0.03151	0.01569	-0.1033	0.5701	-0.01578
cg26853057	0.01294	0.1069	0.9058	-0.006225	0.06885	0.05255
cg26870771	0.02311	-0.0985	0.3353	-0.03671	0.01767	-0.1047
cg26880445	0.8313	0.01311	0.03271	-0.152	0.958	-0.003102
cg26923959	0.01925	-0.07988	0.6932	0.01349	0.01802	-0.1139
cg26929897	0.01932	-0.1443	0.3598	-0.04957	0.0285	-0.1158
cg26934726	0.005874	-0.123	0.2579	-0.03968	0.03835	-0.06347
cg26972422	0.01256	-0.1256	0.01929	-0.0732	0.01823	-0.07452
cg26975943	0.1018	-0.09004	0.803	0.01557	0.04257	-0.1108
cg26980978	0.07676	-0.06692	0.1037	-0.06169	0.01771	-0.1024
cg27013898	0.03794	-0.05666	0.08446	-0.06884	0.02636	-0.1149
cg27055495	0.0236	-0.1001	0.5927	-0.01826	0.05914	-0.07554
cg27059537	0.6583	0.01838	0.03895	-0.1333	0.611	0.02487
cg27063986	0.04314	-0.1087	0.9744	-0.002742	0.2228	-0.06733
cg27070070	0.2189	-0.02853	0.03182	-0.1048	0.1283	-0.0322
cg27070445	0.01317	0.1773	0.1186	0.08625	0.006846	0.2005
cg27079614	0.7765	0.008823	0.03119	-0.1021	0.595	0.01718
cg27087233	0.9221	0.003223	0.03077	-0.1029	0.4729	-0.02464
cg27095866	0.01472	-0.1093	0.2411	-0.03034	0.01431	-0.05216
cg27139244	0.03199	-0.1002	0.6392	-0.01196	0.1054	-0.05722
cg27162842	0.01658	0.1154	0.8788	0.007478	0.196	0.04462
cg27164167	0.5621	0.02262	0.04268	-0.1113	0.325	-0.03748
cg27181047	0.05045	-0.1085	0.4419	-0.044	0.04148	-0.1033
cg27271129	0.1664	-0.04584	0.1125	-0.06038	0.00998	-0.1181
cg27299021	0.01169	-0.1151	0.07973	-0.06421	0.3169	-0.03119
cg27318134	0.04542	-0.1325	0.2675	-0.08718	0.03356	-0.145
cg27343660	0.1409	-0.05138	0.03343	-0.108	0.02959	-0.08628
cg27374435	0.4328	-0.03355	0.07527	-0.09365	0.03333	-0.1361
cg27380552	0.4629	0.02492	0.04172	-0.1181	0.6881	-0.01809
cg27397636	0.02739	-0.1164	0.1702	-0.06589	0.212	-0.06265
cg27421102	0.02965	-0.1091	0.4424	-0.03616	0.3324	-0.0491
cg27423177	0.6519	-0.02334	0.03343	-0.1059	0.9855	0.001247
cg27429832	0.01923	-0.123	0.4163	-0.04419	0.2504	-0.06659
cg27437083	0.2126	0.03957	0.01603	-0.1102	0.4667	0.02963
cg27440731	0.4471	0.0231	0.01465	-0.1238	0.8366	-0.006841
cg27444195	0.03467	-0.09655	0.01906	-0.1056	0.01057	-0.09933
cg27478030	0.06875	-0.08778	0.0474	-0.1126	0.01812	-0.1358
cg27480179	0.03732	0.1071	0.7738	-0.0193	0.1258	0.08904
cg27486714	0.2138	-0.04398	0.01918	-0.1292	0.2116	-0.05294
cg27527887	0.06497	-0.06715	0.1533	-0.06319	0.02109	-0.1012
cg27528748	0.7056	-0.02069	0.5218	0.05197	0.01668	0.1609
cg27533825	0.02284	0.1	0.1703	-0.0542	0.01564	0.1211
cg27534278	0.02297	-0.1191	0.01566	-0.1439	0.2195	-0.049
cg27545977	0.03383	0.1016	0.9555	-0.003	0.01095	0.135
cg27546670	0.04662	-0.1045	0.55	-0.02506	0.1656	-0.06359
cg27589988	0.02022	0.1448	0.3453	-0.0424	0.05149	0.09311
cg27605358	0.01054	-0.1041	0.1106	-0.05372	0.01558	-0.09047
cg27622325	0.01863	-0.1246	0.2778	-0.05067	0.09667	-0.07498
cg27626190	0.2619	-0.04107	0.02362	-0.1058	0.3012	-0.03129
cg27629776	0.2886	-0.07465	0.1545	0.129	0.03835	-0.1387

cg27639094	0.01908	-0.1051	0.0399	-0.08892	0.07222	-0.05426
cg27661952	0.02681	-0.1003	0.9016	-0.005579	0.03207	-0.08725
cg27663476	0.01956	0.1034	0.8228	0.009062	0.04438	0.09022

Table S4.Overlapping differentially methylated positions upon exposure of hMSCs to Mix G1 1X and 1000X.

IlluminaID	Gene	deltaBeta.1x	deltaBeta.1000x	deltaBeta.10x
cg04104489	PGM1	-0.2803	-0.1839	
cg01806142	NA	-0.1946	-0.1489	
cg09307266	BCLAF1	-0.1763	-0.2087	
cg06360274	NA	-0.1756	-0.1126	
cg01877318	CDC27	-0.1753	-0.1272	
cg03941746	SLC11A2	-0.1714	-0.1099	
cg11352430	PDE11A	-0.1543	-0.14	
cg14581997	KCNQ3	-0.1505	-0.1158	
cg18036763	PHF21B	-0.1504	-0.1416	
cg08858393	GRM5	-0.1497	-0.1373	
cg20661257	NA	-0.1496	-0.1302	
cg26702908	ARHGEF26	-0.1476	-0.1645	
cg13899418	PPP4R4	-0.1469	-0.1081	
cg11500790	OPCML	-0.1457	-0.1112	
cg13173809	THRA1/BTR	-0.1445	-0.1065	
cg26929897	ST14	-0.1443	-0.1158	
cg23382549	LINC00862	-0.1438	-0.144	
cg25768573	C5orf66	-0.1435	-0.1586	-0.1361
cg15408326	NA	-0.1422	-0.1107	
cg12982075	NA	-0.1372	-0.129	
cg22256960	NA	-0.1362	-0.1311	
cg03167717	TAL1	-0.1359	-0.1165	
cg03214420	C19orf61	-0.1358	-0.1276	
cg25943727	NA	-0.1351	-0.163	
cg23342296	CACNA2D3	-0.134	-0.1052	
cg06938767	NDRG4	-0.1335	-0.1707	
cg14167415	NA	-0.1329	-0.1406	
cg00743831	NSMCE2	-0.1328	-0.1026	
cg08908482	NA	-0.1327	-0.1277	
cg27318134	NA	-0.1325	-0.145	
cg01843127	NA	-0.1322	-0.1303	-0.1098
cg13881619	FAM129B	-0.1321	-0.1365	
cg05706661	LOC10192861[illegible]	-0.1315	-0.1221	
cg19399100	NA	-0.13	-0.1097	
cg18355137	NDUFAF2	-0.1296	-0.1311	
cg00458868	NA	-0.1286	-0.1129	
cg11252995	NA	-0.1282	-0.1702	
cg22061809	IMPACT	-0.1275	-0.118	
cg00333843	MAF	-0.1273	-0.1044	
cg16781548	NA	-0.1263	-0.1078	
cg22553273	IRS1	-0.126	-0.1551	
cg10294939	NA	-0.1251	-0.1339	
cg16058493	NA	-0.1243	-0.105	
cg01162358	CECR2	-0.1228	-0.1197	
cg21977507	NA	-0.1223	-0.1006	
cg06878960	GTF3C1	-0.1218	-0.1144	
cg00321855	NA	-0.1215	-0.111	
cg09768680	NA	-0.1213	-0.1031	
cg09460999	NA	-0.1204	-0.1367	

cg12081021	CAPN8	-0.1191	-0.1018	-0.1240
cg25342674	SERINC2	-0.1186	-0.1081	-0.1250
cg07162914	MIR548AJ2	-0.1185	-0.144	
cg04653639	FLT1	-0.117	-0.1076	
cg02744670	RNF4	-0.1137	-0.1049	
cg13147462	UQCRB	-0.1134	-0.1178	
cg00456581	NA	-0.1132	-0.1217	
cg04804604	NA	-0.113	-0.1082	-0.1017
cg00299454	NA	-0.1123	-0.1126	
cg25091323	NA	-0.1121	-0.1268	-0.1678
cg14590644	NR6A1	-0.1121	-0.12	-0.1159
cg24425178	NA	-0.1118	-0.109	
cg07346177	DLGAP2	-0.1106	-0.1194	
cg23206194	NA	-0.1103	-0.1216	
cg22036069	NA	-0.1103	-0.1523	
cg24871445	LOC283856	-0.1102	-0.1026	
cg12428218	NA	-0.1097	-0.1104	
cg03062717	FFAR2	-0.1093	-0.1084	
cg21588792	NA	-0.1083	-0.1101	
cg00760931	NA	-0.1071	-0.1315	-0.1166
cg13557530	NA	-0.1066	-0.1123	
cg18412984	WDR20	-0.1065	-0.1004	
cg07267674	NA	-0.1058	-0.1053	
cg08980265	NA	-0.1055	-0.1109	
cg17167021	MAP2	-0.1049	-0.1206	
cg01169259	NA	-0.1043	-0.119	
cg13430943	RBMS1	-0.1028	-0.1203	
cg17906152	GRIA4	-0.1024	-0.1464	
cg05131620	NA	-0.1015	-0.1116	
cg00858624	NA	-0.1014	-0.1322	
cg20938830	ATR	-0.1013	-0.1004	
cg07519598	MAGI2	-0.1009	-0.1523	
cg13939216	WHSC1	0.1004	0.1011	
cg07053668	TCF25	0.1012	0.1047	
cg27545977	NA	0.1016	0.135	
cg26495357	SNTG2	0.1024	0.1362	
cg08411224	GNAQ	0.1026	0.1165	
cg26281107	NA	0.1028	0.1094	
cg10258033	NA	0.1034	0.1384	
cg17760881	NA	0.1042	0.1439	
cg14672496	C18orf8	0.1045	0.1049	
cg01958456	NA	0.1053	0.1018	
cg01840401	TOMM7	0.1068	0.128	
cg05238074	NA	0.1068	0.1177	
cg16393844	NA	0.1075	0.1512	
cg07761273	EGFL6	0.1078	0.1165	
cg07318939	USP11	0.1083	0.115	
cg09540471	AMN	0.1084	0.1149	
cg07717559	RAP1GAP2	0.1106	0.1175	
cg23362669	UXS1	0.1106	0.1001	

cg24245297	CNR1	0.1107	0.111	
cg07961467	IL27RA	0.1113	0.1048	
cg15233880	CCND1	0.1115	0.1131	
cg01061877	KPTN	0.1119	0.1105	
cg01154508	NA	0.1121	0.108	
cg22826902	COX16	0.1122	0.1066	
cg19860548	NA	0.1136	0.1186	
cg20548859	ADARB1	0.1138	0.1032	
cg20970245	LONRF1	0.1152	0.1076	
cg14881187	DMTF1	0.1157	0.1551	
cg19810954	ADARB1	0.1164	0.1198	
cg22985036	POLS	0.1166	0.1423	
cg13870005	PCDHGA1	0.1168	0.1115	
cg18587985	RABL6	0.117	0.1338	
cg06140237	APBB2	0.1177	0.1178	
cg23135286	PHF7	0.1179	0.1474	
cg15555383	NA	0.1189	0.1089	
cg11830721	LOC10192781	0.1209	0.1151	
cg16633394	FHIT	0.1211	0.1041	
cg16696039	FGF9	0.1221	0.1444	
cg02376178	CSNK1G2	0.1223	0.133	
cg18335670	NA	0.1231	0.1395	
cg07053481	TMEM185A	0.1231	0.1028	
cg21921829	ST7	0.1233	0.1316	
cg01101114	LIMA1	0.1234	0.1199	
cg10934670	ARPP21	0.1253	0.1377	
cg04717613	NA	0.1262	0.1048	
cg19268720	PTPRJ	0.1271	0.1079	
cg13551754	ECE1	0.1273	0.1364	
cg11602041	ME3	0.1279	0.101	
cg14565417	ZNRF3	0.1289	0.1274	
cg02592739	NA	0.1306	0.1331	
cg24373618	NA	0.131	0.1328	
cg16915469	PWP2	0.1322	0.1421	
cg02796638	NA	0.1335	0.1454	
cg23728761	LINC00996	0.1342	0.1274	
cg07204602	CYP26C1	0.1345	0.1136	
cg08212576	LINC00571	0.1348	0.1365	
cg01491358	ZZEF1	0.1377	0.1379	
cg04890706	NHLRC3	0.1389	0.1536	
cg10504669	LARGE	0.1392	0.1005	
cg20672131	NA	0.1403	0.134	
cg26434332	CADM1	0.141	0.1251	
cg19452011	ADAMTS16	0.1429	0.1325	
cg23530239	RPS6KA2	0.1442	0.1853	
cg13495667	NA	0.1463	0.1308	
cg16391727	PRKAG2	0.1466	0.1249	
cg23586018	TENM1	0.1468	0.1125	
cg20473723	FERMT1	0.1475	0.1319	
cg03362798	MYOF	0.1477	0.1767	

cg19133443	TMEM26	0.155	0.1383	
cg06771890	NA	0.1582	0.1111	
cg02830467	MYCT1	0.1603	0.1298	
cg01873305	MRPS15	0.1619	0.1528	
cg24310667	KIAA1324L	0.1627	0.1385	
cg16672922	ACSM3	0.1681	0.1697	
cg09417864	FAM149A	0.1733	0.1435	
cg27070445	C9orf171	0.1773	0.2005	
cg11318129	HCFC1	0.2307	0.148	
