# Supplementary material for: A Mixture of Endocrine Disrupting Chemicals Associated with Lower Birth Weight in Children Induces Adipogenesis and DNA Methylation Changes in Human Mesenchymal Stem Cells
Source: Int J Mol Sci. 2022 Feb 19;23(4):2320. doi: 10.3390/ijms23042320 (PMC8879125; doi:10.3390/ijms23042320)
Supplement: Supplementary file 1 [file ijms-23-02320-s001.zip › Supplementary Information.pdf]

A mixture of endocrine disrupting chemicals associated with lower birth weight in children induces adipogenesis and DNA methylation changes in human mesenchymal stem cells

Polina Lizunkova, Elin Engdahl, Gabor Borbély, Chris Gennings, Christian Lindh, Carl-Gustaf Bornehag, Joëlle Rüegg

Supporting Information

Number of pages: S30

Number of Supplementary Figures: 3

Number of Supplementary Tables: 7

Supplementary Methods

Identification and preparation of Mixture G1

The chemical mixture (Mix G1) used experimentally in this study was designed within the EU project EDC-MixRisk (http://edcmixrisk.ki.se/). Levels of 54 compounds were measured in blood and urine of >2,300 pregnant women in median gestational week 10 included in the Swedish Environmental Longitudinal, Mother and Child, Asthma and allergy (SELMA) study (Bornehag et al., 2012). Forty-one compounds (corresponding to 26 parent compounds) out of the 54 measured showed levels above the limit of quantification in more than half of the women, and were therefore included in the statistical analyses. The mixture identification includes three steps, also described in Bornehag et al., (2019):

Firstly, we identified chemicals of concern measured in prenatal urine and serum of the SELMA mothers that were associated with a lower birth weight in their children. Such chemicals of concern were selected using weighted quantile sum (WQS) regression which is a strategy for estimating empirical weights for a weighted sum of quantiled concentrations (e.g., quartile or decile scores) most associated with the health outcome (Carrico, et al., 2015). The results are a beta coefficient associated with the weighted sum (beta, SE, and p value) and the empirical weights (which are constrained to sum to 1). The components most associated with the health outcomes have non-negligible weights, i.e., greater than an a priori threshold value. The threshold values were set in relation to the number of compounds in the models, i.e., from a set of n components with weights that exceed 1/n to be of interest. In the overall analysis including all 41 compounds 1/41=0.024, we considered components

with a weight above 2%, and in the separate analyses of urine and serum compounds including around 20 compounds in each, 1/20=0.050 where we have considered components above 5%. We also analysed boys and girls separately. The following criteria were used to identify a chemical of concern: i) the p value for the model estimating the weights had to be < 0.05 AND ii) the weight for a given analyte must be above the threshold value in the overall model (with 41compounds) of all children and at least in one other model, or, the weights from models of a single sex must exceed the threshold value in at least the overall model and in the corresponding urine or serum model. In this study, WQS regression was used to determine the contribution of the 41 quantified compounds, as a mixture, on a lower birth weight in the offspring in 1,476 mother-child pairs (773 boys and 703 girls) where data was available for the women's exposure levels, the child's birth weight as well as potential confounders (urinary creatinine level, sex of the child, gestational week at birth, parity as well as maternal smoking status, fish intake, and mothers age and weight at enrolment). Fourteen chemicals of concern were identified according to the criteria above (Table S1 and Table S2).

Secondly, we estimated the serum levels of the chemicals of concern. Urine compounds were converted into serum concentrations through estimation of daily intake (Koch et al., 2007 and Fromme et al., 2007) and chemicals measured in serum were used as such, as described in Bornehag et al., (2019).

Thirdly, the mixing proportions of the chemicals of concern were established using biomonitoring data (i.e., serum geometric mean levels) from the SELMA study. These

estimations followed a simplified equation of a one-compartment toxicokinetic model (Fromme et al., 2007).

Figures

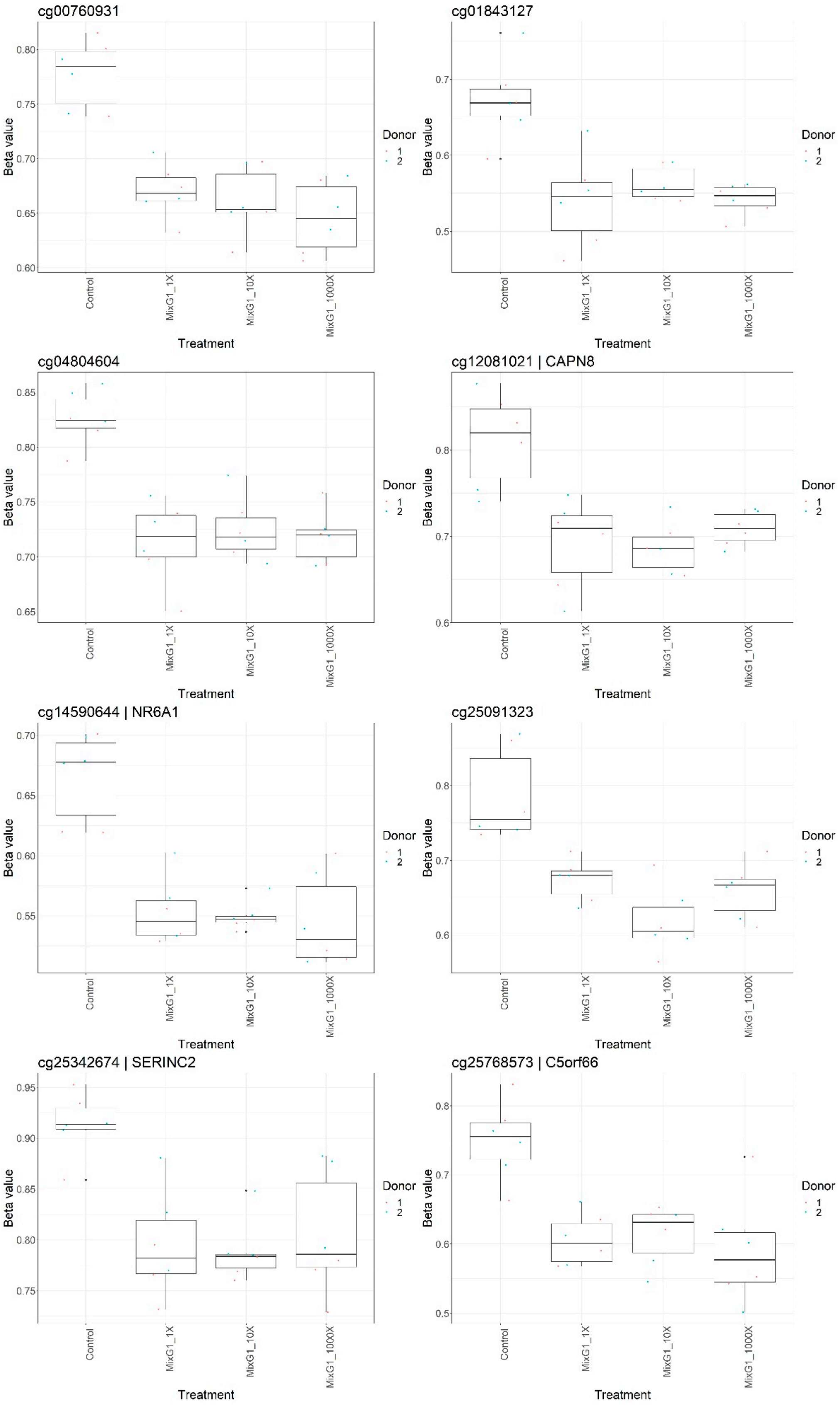
cg00760931
cg01843127
cg04804604
cg12081021 | CAPN8
cg14590644 | NR6A1
cg25091323
cg25342674 | SERINC2
cg25768573 | C5orf66
Beta value
Treatment
Control
MixG1_1X
MixG1_10X
MixG1_1000X
Donor
1
2

Figure S1. Methylation levels of the eight overlapping differentially methylated positions among Mix G1 1X, 10X and 1000X concentrations.

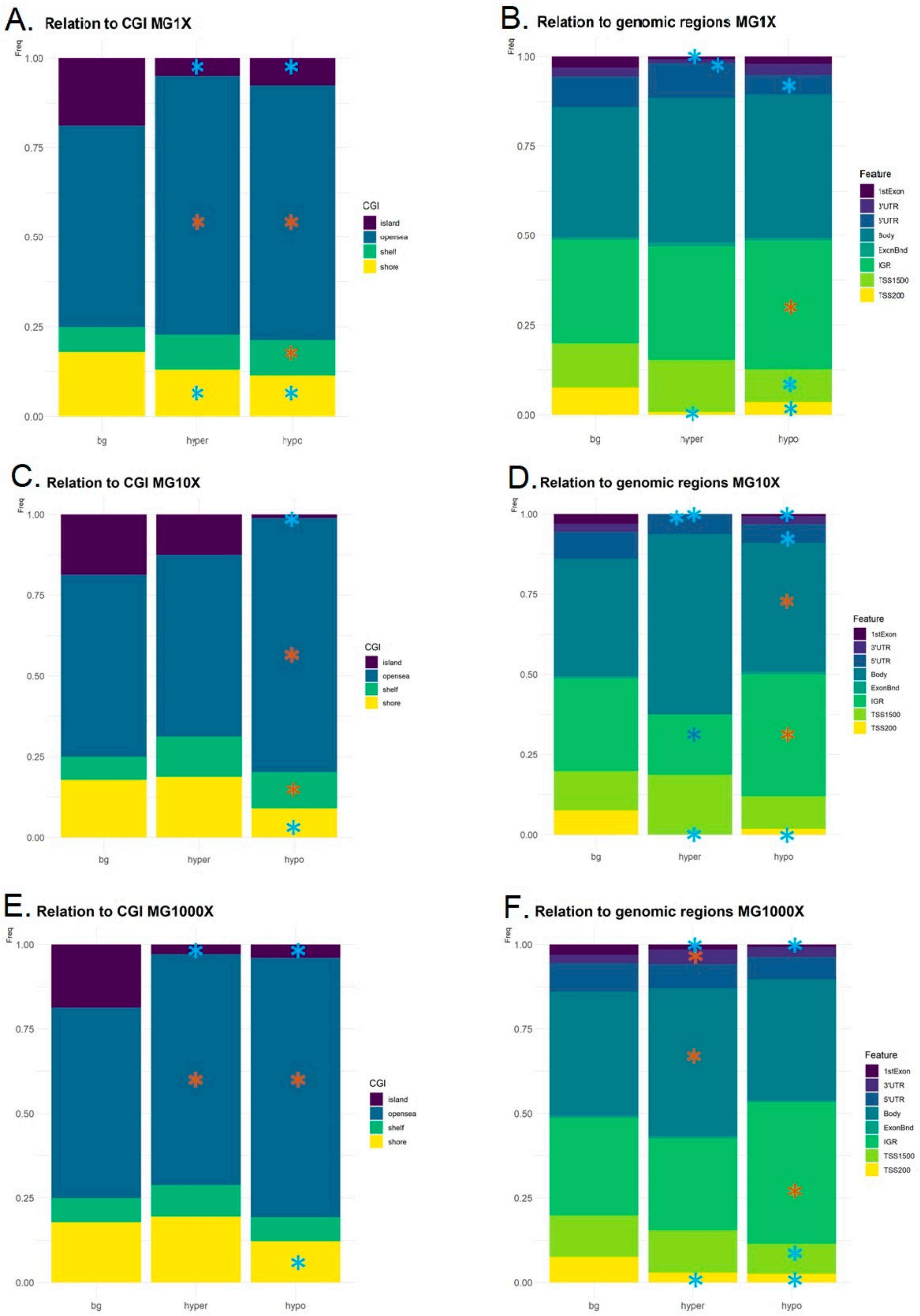
A. Relation to CGI MG1X
B. Relation to genomic regions MG1X
C. Relation to CGI MG10X
D. Relation to genomic regions MG10X
E. Relation to CGI MG1000X
F. Relation to genomic regions MG1000X
Freq
1.00
0.75
0.50
0.25
0.00
bg
hyper
hypo
CGI
island
opensea
shelf
shore
Feature
1stExon
3'UTR
5'UTR
Body
ExonBnd
IGR
TSS1500
TSS200

Figure S2. Genomic distribution of differentially methylated positions (DMPs). The distribution of DMP ($\Delta\beta$ > 10 %, FDR < 0.05) in relation to: CpG islands (CGI) location in **A)**. Mix G1 1X, **C).** 10X, and **E).** 1000X; as genomic location in **B).** Mix G1 1X, **D).** 10X, and **E).** 1000X respectively. The red stars indicate significant enrichment while the blue stars indicate significant depletion (p value < 0.05), relative to the background genome (bg). Hypo- indicates hypomethylated DMPs, hyper- indicates hypermethylated DMPs.

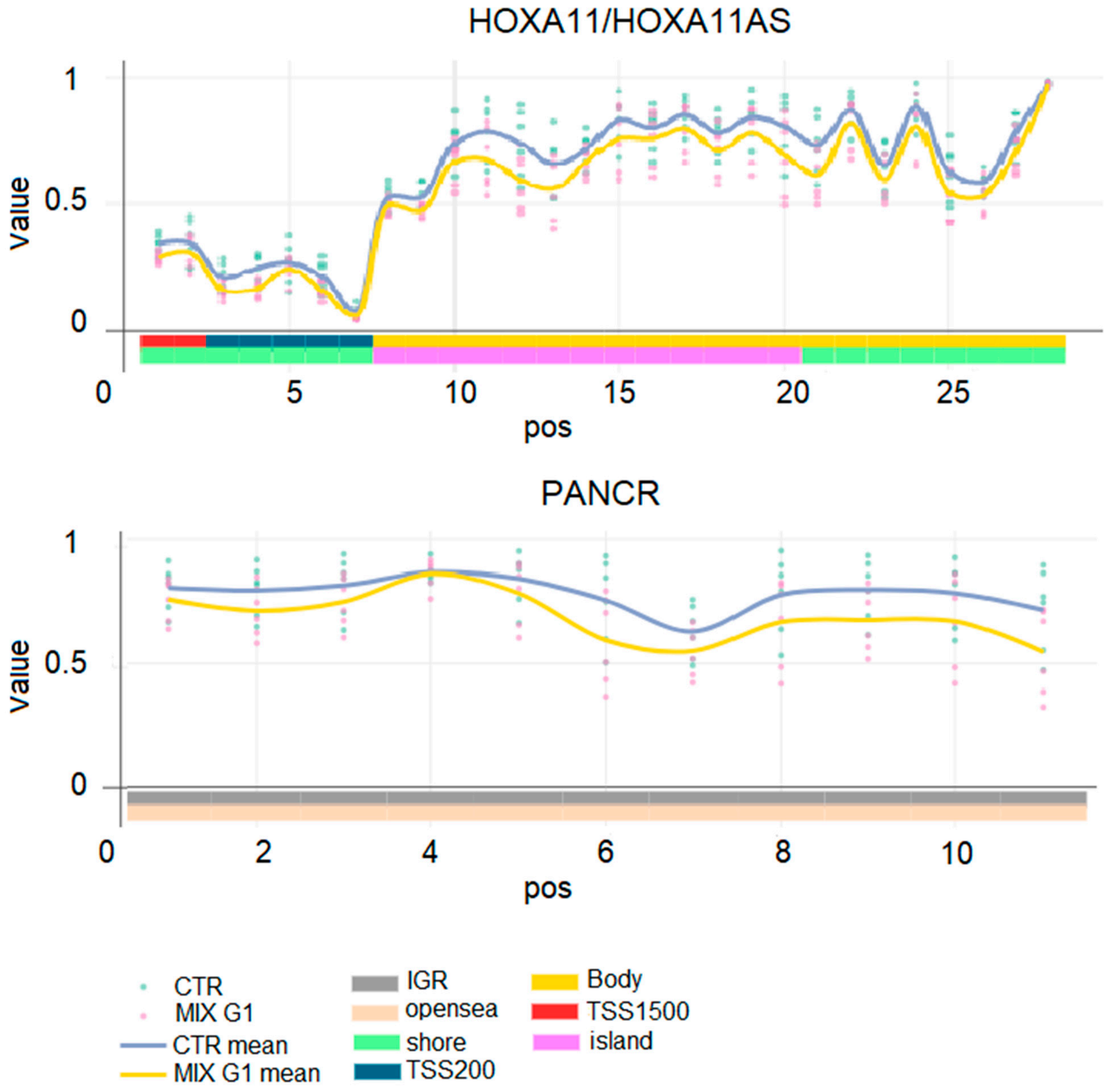
HOXA11/HOXA11AS
value
1
0.5
0
0
5
10
15
20
25
pos
PANCR
value
1
0.5
0
0
2
4
6
8
10
pos
CTR
MIX G1
CTR mean
MIX G1 mean
IGR
opensea
shore
TSS200
Body
TSS1500
island

Figure S3. Identified DMRs upon exposure of hMSCs to Mix G1 1000X. DMR analysis was conducted using the bumphunter method embedded in the ChAMP analysis package. The plots show beta methylation values (Y axis) of each consecutive CpG site (X axis) within each DMR region (not their real MAPINFO distance).

Tables

Table S1. Chemicals of concern (*) for a lower birth weight [Mix G1] identified by WQS regression, results for 22 urine compounds.

**Compound**	**Metabolite**	**Chemicals of concern**	**Weights (%)**					
			**WQS in separate analysis including 22 urine compounds**			**WQS in total analysis including 22 urine and 19 serum compounds (cont. in Table 1b)**		
			All	Boys	Girls	All	Boys	Girls
**Beta**			-24.1	-18.5	-38.7	-60.6	-44.6	-93.6
**SE**			9.0	14.5	12.3	11.0	16.7	15.5
**p-value**			**.0072**	.2025	**.0016**	**<.0001**	**.0078**	**<.0001**
**N**			1697	893	804	1476	773	703
**Threshold**			5			2		
DEP	MEP	*	3,8	NA	**10,1**	1,6	1,3	**5,5**
DBP	MBP	*	**22,9**	NA	**16,1**	**11,6**	**6,4**	**11,2**
BBzP	MBzP	*	**11,5**	NA	**22,4**	**12,9**	**6,5**	**12,8**
DEHP	MCMHP		0,0	NA	1,9	0,0	0,0	1,1

DEHP	MEHP	*	1,6	NA	3,7	**3,1**	1,1	**2,8**
DEHP	MEHHP		0,0	NA	3,3	0,0	0,0	0,7
DEHP	MEOHP		**5,8**	NA	4,9	1,3	0,2	0,9
DEHP	MECPP		0,5	NA	0,4	0,1	0,2	0,8
DINP	MHiNP		0,0	NA	0,0	0,0	0,4	0,0
DINP	MOiNP		0,0	NA	0,0	0,0	0,0	0,0
DINP	MCiOP		0,0	NA	0,0	0,0	0,2	0,0
DINCH	MOiNCH	*	3,0	NA	1,0	**3,8**	**15,4**	0,3
DiDP/DPHP	MHiDP		0,9	NA	0,1	0,7	**5,5**	0,0
DiDP/DPHP	MCiNP		0,3	NA	0,2	0,8	0,3	0,4
	Triclosan	*	**21,1**	NA	**17,3**	**3,5**	**5,2**	**3,6**
	BPA		3,0	NA	2,6	1,5	**3,5**	0,3
	4_4BPF		0,0	NA	0,0	0,0	0,1	0,0
	BPS		0,4	NA	1,8	0,1	0,2	0,5
	2_OH_PH	*	**14,0**	NA	2,9	**6,6**	**4,9**	**4,0**
	DPP	*	**7,0**	NA	4,5	**4,8**	**4,3**	**3,1**
	TCP		0,2	NA	0,0	0,0	1,8	0,0
	3_PBA	*	3,8	NA	**6,9**	1,5	0,6	**4,1**

NA = Not available due to a non-significant WQS index.

Table S2. Chemicals of concern (*) for a lower birth weight [Mix G1] identified by WQS regression, results for 19 serum compounds.

Compound	Meta-bolite	Chemicals of concern	Weights (%)					
			WQS in separate analysis including 19 serum compounds			WQS in total analysis including 22 urine and 19 serum compounds (cont. from Table 1a)		
			All	Boys	Girls	All	Boys	Girls
**Beta**			-29.7	-18.2	-47.7	-60.6	-44.6	-93.6
**SE**			6.9	9.6	10.2	11.0	16.7	15.5
**p-value**			**<.0001**	.0595	**<.0001**	**<.0001**	**.0078**	**<.0001**
**N**			1504	789	715	1476	773	703
**Threshold**			5			2		
PFOA		*	**31,2**	NA	**29,3**	**16,7**	**7,8**	**16,1**
PFOS		*	**14,6**	NA	**21,9**	**3,2**	0,7	**8,3**
PFNA			0,6	NA	2,6	0,3	1,1	0,5
PFDA			0,6	NA	0,7	0,3	0,9	0,3
PFUnDA			0,2	NA	0,5	0,1	0,6	0,2
PFHxS		*	4,0	NA	**6,7**	2,0	**3,6**	**3,9**

HCB		*	**28,5**	NA	**9,9**	**15,9**	**15,2**	**5,2**
T_nanochl.			0,2	NA	0,0	0,4	**4,2**	0,0
p_p_DDE		*	**11,4**	NA	**10,0**	**5,2**	**4,4**	**5,7**
PCB_74			**6,9**	NA	4,1	1,4	**3,0**	1,7
PCB_99			0,1	NA	1,6	0,2	0,0	0,9
PCB_118[1]			1,6	NA	**7,0**	0,4	0,2	**3,8**
PCB_138			0,0	NA	0,6	0,0	0,0	0,1
PCB_153			0,0	NA	0,3	0,0	0,0	0,2
PCB_156			0,0	NA	0,0	0,0	0,0	0,0
PCB_170			0,0	NA	0,4	0,0	0,0	0,1
PCB_180			0,0	NA	0,1	0,0	0,0	0,0
PCB_183			0,0	NA	3,8	0,0	0,0	0,9
PCB_187			0,0	NA	0,4	0,0	0,0	0,1

NA = Not available due to a non-significant WQS index.

[1]PCB_118 was identified as a chemical of concern but not included in Mix G1 because its molar concentration was orders of magnitude below the others.

Table S3. Differentially methylated positions upon exposure of hMSCs to Mix G1 1X, 10X, and 1000X. See PDF file named Supplementary Table S3-4.

Table S4. Overlapping differentially methylated positions upon exposure of hMSCs to Mix G1 1X and 1000X. See PDF file named Supplementary Table S3-4.

Table S5. Differentially methylated regions (DMRs) upon exposure of hMSCs to Mix G1 1X.

**Gene**	**Illumina ID**	**CHR**	**Feature**	**CGI**
HOXA11AS/HOXA11	cg17950095	7	TSS1500	shore
	cg08479590	7	TSS1500	shore
	cg01326836	7	TSS200	shore
	cg00608008	7	TSS200	shore
	cg21447117	7	TSS200	shore
	cg18977999	7	TSS200	shore
	cg24932686	7	TSS200	shore
	cg05977669	7	Body	island
	cg24446586	7	Body	island

	cg13352750	7	Body	island
	cg15916646	7	Body	island
	cg10767141	7	Body	island
	cg15760840	7	Body	island
	cg09661370	7	Body	island
	cg12810084	7	Body	island
	cg09495769	7	Body	island
	cg24988255	7	Body	island
	cg25901381	7	Body	island
	cg17466857	7	Body	island
	cg10657141	7	Body	island
	cg07116997	7	Body	shore
	cg01729491	7	Body	shore
	cg04340874	7	Body	shore
	cg18978493	7	Body	shore
	cg16608407	7	Body	shore
	cg04822748	7	Body	shore

	cg16038003	7	Body	shore
	cg00705992	7	Body	shore
PM20D1	cg03461704	1	Body	shore
	cg06815965	1	Body	shore
	cg17178900	1	Body	island
	cg14159672	1	1stExon	island
	cg14893161	1	5'UTR	shore
	cg07533224	1	TSS200	shore
	cg12898220	1	TSS200	shore
	cg05841700	1	TSS200	shore
	cg11965913	1	TSS200	shore
	cg07167872	1	TSS200	shore
	cg24503407	1	TSS1500	shore
	cg16334093	1	TSS1500	shore
	cg07157834	1	TSS1500	shore
PANCR	cg06164973	16	IGR	opensea
	cg08317243	16	IGR	opensea

	cg06050193	16	IGR	opensea
	cg05547279	16	IGR	opensea
	cg06887778	16	IGR	opensea
	cg26866349	16	IGR	opensea
	cg09610558	16	IGR	opensea
	cg04920214	16	IGR	opensea
	cg01953615	16	IGR	opensea
	cg00275103	16	IGR	opensea
	cg26994894	16	IGR	opensea
HOXA5	cg17569124	7	TSS1500	island
	cg02005600	7	TSS1500	island
	cg25307665	7	TSS1500	island
	cg14014955	7	TSS1500	island
	cg02646423	7	TSS1500	island
	cg23204968	7	TSS1500	island
	cg14058329	7	TSS1500	island
	cg03207666	7	TSS1500	island

	cg23454797	7	TSS1500	island
	cg12015737	7	TSS1500	island
	cg08070327	7	TSS1500	island
	cg25506432	7	TSS1500	island
	cg16997642	7	TSS1500	island
	cg14013695	7	TSS1500	island
	cg25390165	7	TSS1500	island
	cg05774699	7	TSS1500	island
	cg26023912	7	TSS1500	island
	cg14882265	7	TSS1500	island
	cg17432857	7	TSS1500	island
	cg00969405	7	TSS1500	island
	cg07049592	7	TSS1500	island
	cg02106682	7	TSS1500	island
	cg03368099	7	TSS1500	island
	cg01748892	7	TSS1500	island
	cg13694927	7	TSS1500	island

	cg03744763	7	TSS1500	island
	cg27151303	7	IGR	island
	cg05579037	7	IGR	island
RP11-134D3.2	cg10699857	4	IGR	island
	cg23404248	4	IGR	island
	cg12652641	4	IGR	island
	cg05722981	4	IGR	shore
	cg00126148	4	IGR	shore
	cg05103917	4	IGR	shore
	cg02719154	4	IGR	shore
	cg11930400	4	IGR	shore
	cg04355159	4	IGR	shore
RPL28	cg12520919	19	TSS1500	island
	cg17387916	19	TSS1500	island
	cg15162827	19	TSS200	island
	cg27067379	19	TSS200	island
	cg18355869	19	TSS200	island

	cg23656673	19	TSS200	island
	cg21109867	19	5'UTR	island
	cg02608292	19	5'UTR	island
	cg07577439	19	5'UTR	island
	cg12122109	19	5'UTR	island
	cg09724295	19	5'UTR	island
	cg01863335	19	5'UTR	island
	cg26519799	19	Body	island

Table S6. Differentially methylated regions upon exposure of hMSCs to Mix G1 1000X.

Gene	IlluminaID	CHR	Feature	Cgi
HOXA11	cg17950095	7	TSS1500	shore
	cg08479590	7	TSS1500	shore
	cg01326836	7	TSS200	shore
	cg00608008	7	TSS200	shore
	cg21447117	7	TSS200	shore
	cg18977999	7	TSS200	shore
	cg24932686	7	TSS200	shore
	cg05977669	7	Body	island
	cg24446586	7	Body	island
	cg13352750	7	Body	island
	cg15916646	7	Body	island
	cg10767141	7	Body	island
	cg15760840	7	Body	island
	cg09661370	7	Body	island

	cg12810084	7	Body	island
	cg09495769	7	Body	island
	cg24988255	7	Body	island
	cg25901381	7	Body	island
	cg17466857	7	Body	island
	cg10657141	7	Body	island
	cg07116997	7	Body	shore
	cg01729491	7	Body	shore
	cg04340874	7	Body	shore
	cg18978493	7	Body	shore
	cg16608407	7	Body	shore
	cg04822748	7	Body	shore
	cg16038003	7	Body	shore
	cg00705992	7	Body	shore
PANCR	cg06164973	16	IGR	opensea
	cg08317243	16	IGR	opensea
	cg06050193	16	IGR	opensea

	cg05547279	16	IGR	opensea
	cg06887778	16	IGR	opensea
	cg26866349	16	IGR	opensea
	cg09610558	16	IGR	opensea
	cg04920214	16	IGR	opensea
	cg01953615	16	IGR	opensea
	cg00275103	16	IGR	opensea
	cg26994894	16	IGR	opensea

Table S7. Gene Ontology enrichment analysis of genes containing DMRs upon exposure to Mix G1 1X.

ONTOLOGY	TERM	Count*	FDR**
BP	nucleic acid metabolic process	163	0.002
BP	nucleobase-containing compound metabolic process	173	0.004
BP	mRNA metabolic process	42	0.007
BP	organic cyclic compound metabolic process	182	0.008
BP	RNA catabolic process	26	0.008
BP	cellular aromatic compound metabolic process	176	0.008
BP	macromolecule metabolic process	262	0.008
BP	mRNA catabolic process	24	0.009
BP	gene expression	176	0.009
BP	cellular nitrogen compound metabolic process	187	0.009
BP	heterocycle metabolic process	174	0.009
BP	RNA metabolic process	144	0.013
BP	protein targeting to ER	11	0.013

BP	protein targeting	25	0.014
BP	SRP-dependent cotranslational protein targeting to membrane	10	0.014
BP	regulation of gene expression	150	0.014
BP	establishment of protein localization to endoplasmic reticulum	11	0.016
BP	nucleobase-containing compound catabolic process	29	0.016
BP	negative regulation of metabolic process	111	0.017
BP	nuclear division	25	0.017
BP	cotranslational protein targeting to membrane	10	0.017
BP	negative regulation of gene expression	83	0.017
BP	regulation of metabolic process	200	0.017
BP	endocrine system development	13	0.017
BP	cellular macromolecule metabolic process	227	0.017
BP	nuclear-transcribed mRNA catabolic process, nonsense-mediated decay	11	0.018
BP	negative regulation of macromolecule metabolic process	104	0.018
BP	metabolic process	300	0.020

BP	cellular nitrogen compound catabolic process	30	0.020
BP	cellular nitrogen compound biosynthetic process	145	0.022
BP	regulation of macromolecule metabolic process	186	0.022
BP	nuclear-transcribed mRNA catabolic process	15	0.026
BP	organic cyclic compound catabolic process	31	0.026
BP	aromatic compound catabolic process	30	0.027
BP	organelle fission	26	0.029
BP	cellular metabolic process	279	0.029
BP	regulation of RNA metabolic process	117	0.032
BP	heterocycle catabolic process	29	0.036
BP	protein targeting to membrane	14	0.036
BP	cellular macromolecule catabolic process	48	0.037
BP	macromolecule catabolic process	55	0.037
BP	regulation of nucleobase-containing compound metabolic process	123	0.037
BP	organic substance metabolic process	286	0.037

BP	translational initiation	13	0.040
BP	amide biosynthetic process	36	0.046
BP	peptide biosynthetic process	31	0.049
CC	nucleus	219	0.002
CC	intracellular membrane-bounded organelle	288	0.009
CC	nuclear lumen	156	0.009
CC	intracellular organelle	324	0.014
CC	ribonucleoprotein complex	33	0.016
CC	cytosolic ribosome	10	0.016
CC	ribosome	15	0.020
CC	ribosomal subunit	13	0.021
CC	nucleoplasm	127	0.023
CC	membrane-enclosed lumen	177	0.029
CC	organelle lumen	177	0.029
CC	intracellular organelle lumen	177	0.029
CC	membrane-bounded organelle	318	0.036

CC	protein-containing complex	153	0.037
MF	nucleic acid binding	128	0.008
MF	structural constituent of ribosome	13	0.012
MF	RNA binding	62	0.042

* count indicates the number of enriched genes in a pathway

** FDR is an adjusted p value.
